# Systematic and Evolutionary Insights Derived from mtDNA COI Barcode Diversity in the Decapoda (Crustacea: Malacostraca)

**DOI:** 10.1371/journal.pone.0019449

**Published:** 2011-05-12

**Authors:** Joana Matzen da Silva, Simon Creer, Antonina dos Santos, Ana C. Costa, Marina R. Cunha, Filipe O. Costa, Gary R. Carvalho

**Affiliations:** 1 Molecular Ecology and Fisheries Genetics Laboratory, School of Biological Sciences, Environment Centre for Wales, Bangor University, Bangor, Wales, United Kingdom; 2 Centro de Estudos do Ambiente e do Mar, Departamento de Biologia, Universidade de Aveiro, Aveiro, Portugal; 3 Instituto Nacional de Recursos Biológicos - L- IPIMAR, Lisboa, Portugal; 4 Departamento de Biologia, Universidade dos Açores, São Miguel, Portugal; 5 Centro de Biologia Molecular e Ambiental (CBMA), Departamento de Biologia, Universidade do Minho, Braga, Portugal; Biodiversity Insitute of Ontario - University of Guelph, Canada

## Abstract

**Background:**

Decapods are the most recognizable of all crustaceans and comprise a dominant group of benthic invertebrates of the continental shelf and slope, including many species of economic importance. Of the 17635 morphologically described Decapoda species, only 5.4% are represented by COI barcode region sequences. It therefore remains a challenge to compile regional databases that identify and analyse the extent and patterns of decapod diversity throughout the world.

**Methodology/Principal Findings:**

We contributed 101 decapod species from the North East Atlantic, the Gulf of Cadiz and the Mediterranean Sea, of which 81 species represent novel COI records. Within the newly-generated dataset, 3.6% of the species barcodes conflicted with the assigned morphological taxonomic identification, highlighting both the apparent taxonomic ambiguity among certain groups, and the need for an accelerated and independent taxonomic approach. Using the combined COI barcode projects from the Barcode of Life Database, we provide the most comprehensive COI data set so far examined for the Order (1572 sequences of 528 species, 213 genera, and 67 families). Patterns within families show a general predicted molecular hierarchy, but the scale of divergence at each taxonomic level appears to vary extensively between families. The range values of mean K2P distance observed were: within species 0.285% to 1.375%, within genus 6.376% to 20.924% and within family 11.392% to 25.617%. Nucleotide composition varied greatly across decapods, ranging from 30.8 % to 49.4 % GC content.

**Conclusions/Significance:**

Decapod biological diversity was quantified by identifying putative cryptic species allowing a rapid assessment of taxon diversity in groups that have until now received limited morphological and systematic examination. We highlight taxonomic groups or species with unusual nucleotide composition or evolutionary rates. Such data are relevant to strategies for conservation of existing decapod biodiversity, as well as elucidating the mechanisms and constraints shaping the patterns observed.

## Introduction

In recent decades, the loss of biodiversity has been recognized as a major global environmental problem, with much effort being targeted at biodiversity conservation [1]–[5]. Yet, a major obstacle in studying the human impact on the biosphere is what has often been referred to as the 'taxonomic impediment': a lack of taxonomic expertise in many groups of living organisms [6] and also the morphological variability associated with such phenotypic plasticity [7], [8] or dimorphism [9]. Biodiversity assessments that are based primarily on morphological characters not only are labour intensive, but risk also under – or over-estimation of biodiversity [10]. To overcome such problems, a short, standardized 650 bp sequence of the cytochrome *c* oxidase subunit 1 (COI) mitochondrial DNA (mtDNA) has been proposed as a barcoding tool, or at least to confirm species delimitation for taxonomic, ecological and evolutionary studies [11]–[17]. The NCBI GenBank molecular database demonstrates that, amongst others (e.g. 16 S, with >7000 entries), COI is one of the most frequently used genes (>10 000 nucleotides entries) for ecological and evolutionary studies of Decapoda, and augmenting these records will enhance the comparative value of such standardised approaches. Specifically, COI as a barcoding tool helps to identify an organism based on DNA sequence variability and assignment to a certain species previously described [10]. Also the DNA barcode sequences can be used as a DNA taxonomy tool to perform prediction and classification of potentially new species. Although the approach remains controversial, [14], [18]–[22], barcoding datasets are rapidly accumulating as part of the worldwide campaign for inventories of global biodiversity [12], [23]–[27]. The impacts of DNA barcoding is extended well beyond biodiversity science. By assembling sequence information for a single gene region from all species, in contrast to the usual focus of large scale genomics projects which acquire sequence information for all genes in single taxa, DNA barcodes can provide a quick preview of recent evolutionary history [28]. For example, data have revealed key features of the mitochondrial genome with implications on the role of selection, as well as highlighting taxonomic groups or species with unusual nucleotide composition or evolutionary rates [28], [29]. The growing volume of barcode records has revealed that sequence variability within species is generally much lower than divergence among species, commonly referred to as the “barcoding gap”, a pattern that occurs in diverse lineages, suggesting a pervasive evolutionary process [12], [17], [23]. The barcode region is a genomic sentinel; shifts in the nucleotide composition of the barcode region in the animal kingdom closely mirror those in the rest of the mitochondrial genome. The classical pairwise distance method such as Neighbour Joining (NJ) based on Kimura 2-parameter distance (K2P) is currently the predominant approach used to analyse patterns of diversity with COI barcode region. It has been informative at the species-level discrimination across a variety of groups from terrestrial, marine and freshwater environments [30]–[35]. The accuracy of such results depends especially on the delineation between intraspecific variation and interspecific DNA sequence divergence, [36], [37]. A threshold barcoding gap was proposed to define species boundaries of around 10 times the mean value for within species variation for the focal group [37]–[39]. More specifically, the proposed threshold value of 2% COI sequence divergence [12], and 0.16 patristic distances for species delimitation in Crustacea [10] may, however, be problematic in some cases (i.e., heteroplasmy, hybridization, incomplete lineage sorting, nuclear introgression of mtDNA [38]–[42]) because DNA barcoding follows the typological species approach and species are entities continue to evolve. To cope with such limitations, DNA barcode sequences have been analysed based on other species concepts [38], and referred to as Recognizable Taxonomic Units [43], or Molecular Operational Taxonomic Units [44].

Barcode sequences can be used to flag species whose mitochondrial genomes show unusual nucleotide composition and rates of amino acid change, thereby identifying lineages that merit more investigation. Other approaches of diversity assessment involve the examination of variation of nucleotide GC content across taxonomic groups to detect unusual variation in mitochondrial GC content [28], [29], [35]. However, the question of the functional significance of this GC variation remains controversial. It is not clear if it has adaptive significance , a by-product of neutral evolutionary processes or if it has actually any significant impact on the phenotype [45].

Decapods are the most recognizable of all crustaceans [46], [47], and include the “true” crabs (Brachyura), hermit crabs and their relatives (Anomura), shrimps (Dendrobranchiata, Caridea and Stenopodidea), and lobsters (Astacidae, Thalassinidea), among other lesser known groups [47]. Establishing a robust DNA barcoding framework for decapods is particularly relevant because the order contains over 17,000 species [46], some of which support seafood and marine industries worth billions of dollars each year to the global economy. Estimates by the Food and Agriculture Organization of the United Nations (FAO), indicated that landings of crustaceans represented about 7% of the total marine fish production in 2007, of which 83% were marine decapods.

Conservation and management of decapods have long been entirely focused on crustacean fisheries [48], [49], but they also form a dominant functional group of megabenthic invertebrates on the Atlantic continental shelf and slope [50]–[54], encompassing a wide range of trophic levels [55] and a variety of feeding habits [56]. In view of their collective ecological importance and potential community interactions, the unambiguous delimitation of species becomes even more urgent.

Of the 17635 morphologically described freshwater and marine extant species [46], only 5.4% are represented by COI barcode region sequences. There is no global campaign yet to barcode crustaceans or decapods, as exists for other animal groups (e.g., fish, birds and lepidopterans). It therefore remains a challenge to compile regional databases that enable analysis of the extent and patterns of decapod diversity throughout the world. Here, using the most comprehensive COI data set for decapods so far examined, we analyse patterns of COI variability partitioning within and among species, genera and families. The combined dataset includes GenBank published sequences, COI barcode projects from the Barcode of Life Database (BOLD), [57] and new data generated herein (Table 1). Collectively, the combined dataset provide barcoding coverage for 1572 sequences of 528 species, 213 genera, and 67 families. Our molecular systematic assessment affords an opportunity to examine the utility of COI DNA barcodes for species recognition in a taxonomically complex and ecologically important group of organisms. We encompass in our study specimens with a range of different shapes (shrimp, lobsters, crayfish and crabs) and sizes (e.g., small crab (Porcellanidae: *Petrolisthes* spp) and big crab (Majidae: *Hyas* spp). Comprehensive biogeographic representation of species was achieved by including species from continental freshwater (e.g., Atyidae and Parastacidae family), brackish (e.g., Palaemonidae and Panopeidae) and marine realms with a high range of latitudinal distribution. On the basis of their latitudinal distribution, decapods from temperate or cold (e.g., Lithodidae: *Lithodes* spp) to tropical waters (e.g., Xiphocarididae: *Xiphocaris* spp) across a range of depth distribution (e.g., Galatheidae) were compared. Species with diverse ecological habits, including such sex reversal (e.g., Palaemonidae), association of shrimps (e.g., Palaemonidae) and crabs (e.g., Pagurus) with other animals and dispersal behaviour (e.g., Pandalidae and Portunidae) were also represented in our analysis. Despite the relatively small proportion of decapods that are considered here, the samples analysed collectively encompass the breadth of morphological and ecological diversity of the order.

**Table 1:** Combined data set derived from new data generated herein and publicly available DNA barcoding projects from the Barcode of Life Database.

Projects	Code	No. of sequences	Species	Citation
**BOLD public projects title**				
Genbank Crustacea Malac - Decapoda	GBCMD	894	349	GenBank
Genbank Crustacea Malac - Decapoda - Atyidae	GBCDA	85	23	GenBank
Genbank Crustacea Malac - Decapoda - Palaemonidae	GBCDP	89	39	GenBank
Genbank Crustacea Malac - Decapoda - Parastacidae	GBCPA	161	59	GenBank
Crustaceans of the St. Lawrence Gulf	WWGSL	130	30	[132]
Decapods of Pacific and Atlantic	FCDPA	118	57	[35]
**Campaign Marine Life (MarBOL)**				
Decapods of Norway, Svalbard, U.K (Scotland), U.K (Wales and England), Mediterranean Sea	JSDN; JSDSV; JSDSC; JSDUK; JSDME	159	52	This study
**Campaign Portugal – Aquatic Life**				
Decapods of Portugal (Hermes, Ipimar, IpimarX, Azores)	FCDPH; FCDOP; JSDPX; JSDAZ	270	82	This study

## Results

### Data acquisition: new sequencing

Here we created new COI sequences of 497 specimens from a total of 101 species, 72 genera and 46 families (Table S1), of which 81 species, 48 genera and 13 families are exclusive of our generated data. The number of sequences per species varied between 1–32, with a mean of 5, and an average length of 620 base pairs (bp). Within this newly-generated dataset, 3.6% of the species barcodes conflicted with the assigned morphological taxonomic identification. Such cases were distributed throughout the Decapoda, including the long legged crabs *Macropodia longipes* (A. Milne Edwards & Bouvier, 1899) and *M. tenuirostris* (Leach, 1814) (Brachyura:Majidae) and the marbled rock crabs *Pachygrapsus maurus* (Lucas, 1846) and *P. marmoratus* (Fabricius, 1787) (Brachyura:Grapsidae) are represented by two “mixed” clades in a NJ tree (Figure S1).

### Data validation

**Figure 1:**
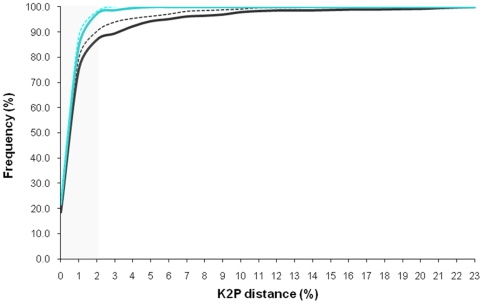
Intraspecific diversity assessment: the effect of sampling bias, non-monophyletic clades, putative cryptic species and congeneric species with low genetic distance. Solid lines represent the raw data for the total data set (A_R_, black lines) and for the dataset in which non-monophyletic clades, putative cryptic species and congeneric species with low genetic distance were removed (B_R_, blue lines). The dashed lines represent results for the data in which all taxa have the same weight (mean values of genetic distance), for the total (black, A_M_) and trimmed (blue, B_M_) datasets respectively.

From a theoretical point of view, two main factors may bias our intraspecific assessment of COI divergence: disequilibrium in the representation of some taxa or incorrect taxonomic classification (i.e., cryptic (morphological indistinguishable, but genetically distinct), or non-monophyletic species). The analysis of the combined GenBank and our novel data (Table S2) indicated the existence of sample bias (*p*<0.05) as shown in Figure 1 (case A_R_, for the entire symbols definition see Methods section). However when putative cryptic (16 species), non-monophyletic (47 species) and con-generic species with unusually low genetic distance (13 species under 2% K2P) were removed from the dataset (case B_R_) (Table S3), the sample bias was lost (p>0.05). Assuming a intraspecific barcode threshold of maximum 2% (K2P), the success of achieving congruent species assignments (Additional File 5) was 97.3% and 98% when mean intraspecific divergences values were compared (in case B_R_ vs B_M_, Figure 1).

In order to reduce the impact of artefacts in our divergence assessment, statistical tests were performed among: raw data A_R_ vs B_R_, mean data A_M_ vs B_M_ and between different data A_R_ vs A_M_ and B_R_ vs B_M_ proposed by Lefébure *et al*., [10]. The first three comparisons revealed sampling bias due to incorrect taxonomic classification and non-monophyletic taxa (p>0.05) and B_R_ vs B_M_ showed no sample bias (p<0.05) indicating a balanced design.

### COI divergence assessment

**Figure 2:**
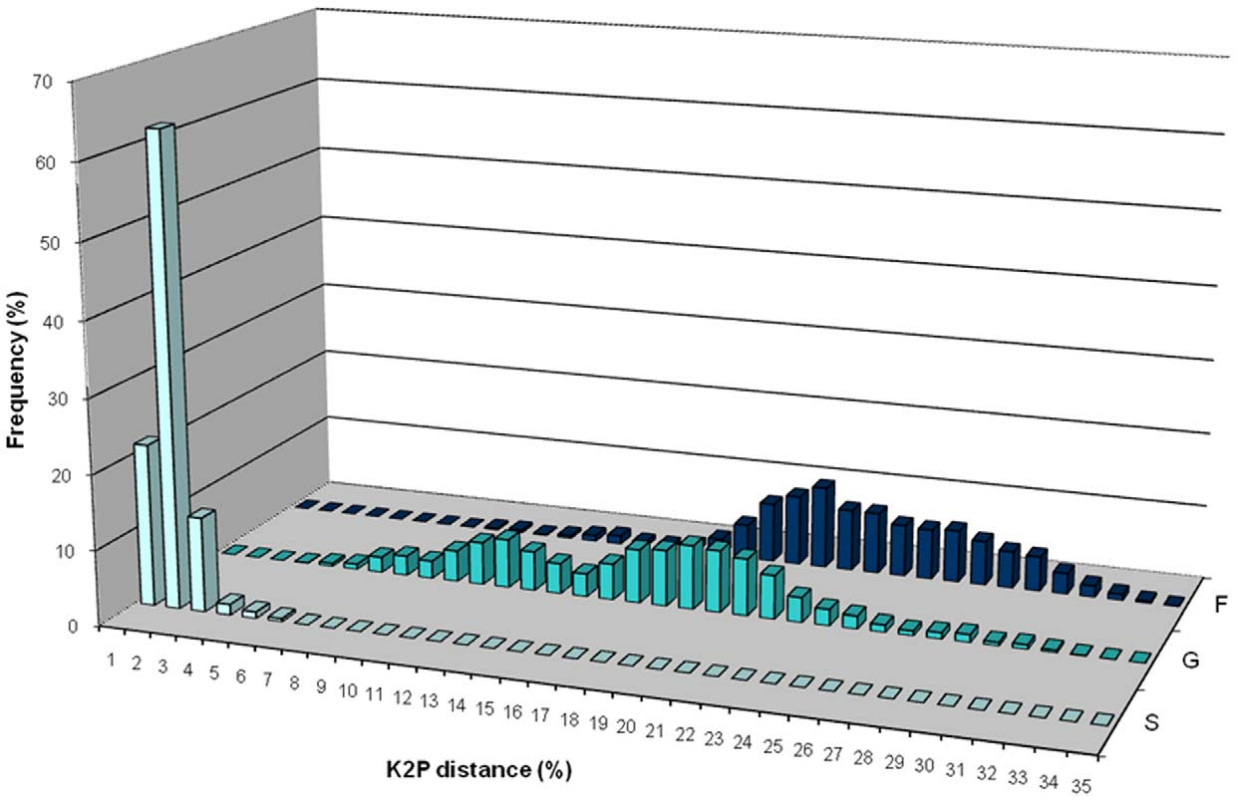
Frequency distribution of COI K2P distances (%) intraspecies (S), intragenus (G), and intrafamily (F) from 302 species, 154 genera, and 58 families.

**Figure 3:**
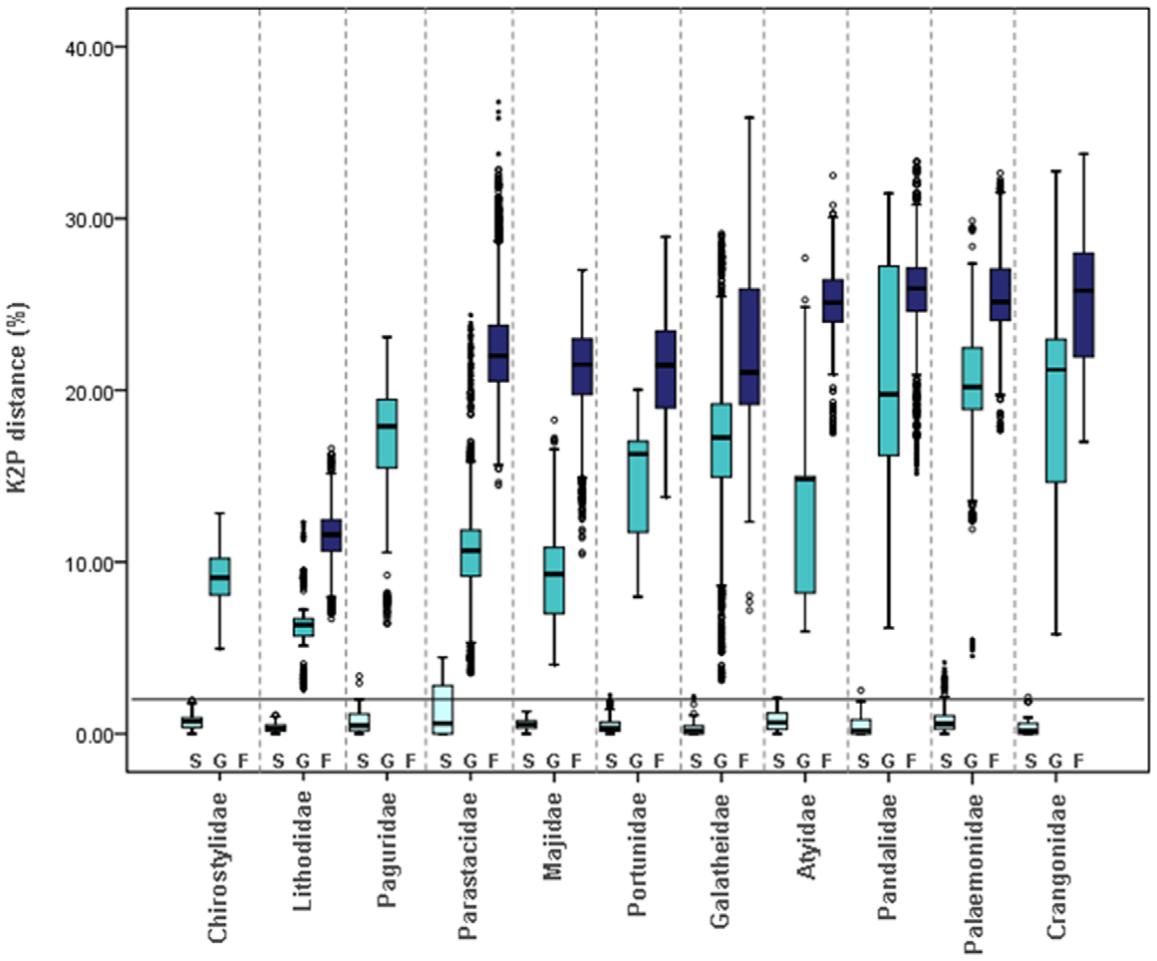
Boxplot distribution of 11 selected families of the Decapoda order intraspecies (S), intragenus (G), and intrafamily (F) COI K2P distances (%). The plot summarises median (central bar), position of the upper and lower quartiles (called Q1 and Q3, central box), extremes of the data (dots) and very extreme points of the distribution that can be considered as outliers (stars). Points are considered as outliers when they exceed Q3+1.5(Q3-Q1) for the lower part, where (Q3-Q1) is the inter quartile range. The number of sequences, species, and genera per family are given in Table 3. Mean K2P distance (%) ± SE within taxa are: Chirostylidae S = 0.701±0.028 and G = 8.999±.039; Lithodidae S = 0.416±0.021, G = 6.376±.137 and F = 11.392±0.063; Paguridae S = 0.686±.045 and G = 17.173±0.084; Parastacidae S = 1.375±0.131, G = 11.017±0.078 and F = 22.681±0.064; Majidae S = 0.547±0.028, G = 9.643±0.214 and F = 21.084±0.061; Portunidae S = 0.453±0.024, G = 14.826±0.311 and F = 28.929±0.047; Galatheidae S = 0.285±0.017, G = 16.839±0.04 and F = 22.355±0.033; Atyidae S = 0.758±0.041, G = 13.475±0.352 and F = 25.218±0.056; Pandalidae S = 0.49±0.042, G = 20.924±0.213 and F = 25.617±0.07; Palaemonidae S = 0.812±0.055, G = 20.157±0.108 and F = 25.398±0.048; Crangonidae S = 0.344, G = 19.991±0.514 and F = 25.241±0.103.

COI barcode nucleotide divergences were calculated for the validated dataset from 1572 sequences of 528 species, 213 genera, and 67 families (B_R_) (Table S4) to reduce the impact of artefacts in our divergence assessment. Sample sizes and mean divergences at various taxonomic levels are given in Table 2. As expected, genetic divergence increased with higher taxonomic rank: 0% to 4.6% within species, 2.5% to 32.7% within genera, and 6.6% to 48.3% within families. Although these ranges overlap, intraspecific (S), intragenus (G) and intrafamily (F) distances (Figures 2 and 3), were significantly different (*p*<0.001). Patterns within families (Table 3 and Figure 3) show a general predicted molecular hierarchy, but the scale of divergence at each taxonomic level appears to vary extensively between families. The range values of mean K2P distance observed were: within species 0.285% to 1.375%, within genus 6.376% to 20.924% and within family 11.392% to 25.617%. The Galatheidae showed the lowest divergence within species (0.285 %), and Lithodidae showed the lowest divergence within genus (6.376%) and within family (11.392%) distances: the highest values were observed within the Pandalidae (within genus: 20.924%) and Parastacidae (within species: 1.375% and within family: 25.617%). The Crangonidae showed the highest range of divergences within a family, the Pandalidae within genus and Parastacidae within species (Figure 3). No sample bias was detected in the within family analysis (*p*>0.05). The Parastacidae was the only family exhibiting sample bias (*p*<0.05), arising from the unbalanced distribution of data with 53% of the sequences being derived from the *Euastacus*, and 21% from the *Cherax* genera.

**Table 2:** Pairwise COI barcode nucleotide divergences for the Decapoda using K2P distances (%).

Decapoda^a^		No. of comparisons	Min Dist	Mean Dist^b^	Max Dist
(1572 seq ,528 sp, 213 gen, 67 fam)					
	Intraspecies	3577	0	0.541±0.01	4.605
	Intragenus	18077	2.509	15.49±0.04	32.75
	Intrafamily	35422	6.694	22.325±0.023	48.348
	Intraorder	1176159	8.509	26.07±0.003	54.994

a Number of sequences, species (sp), genera (gen) and families (fam) are shown in parentheses.

b Data reported as K2P distances (%) ± SE.

**Table 3:** Number of Decapoda sequences, species, genera and families analyzed in the present study.

Family	Species	Genus	Sequences
**Atyidae**	16	9	59
**Chirostylidae**	13	1	66
**Crangonidae**	16	7	58
**Galatheidae**	84	10	220
**Lithodidae**	12	6	52
**Majidae**	24	14	67
**Paguridae**	11	1	57
**Palaemonidae**	32	5	87
**Pandalidae**	19	5	74
**Parastacidae**	43	8	98
**Portunidae**	22	10	90

The majority (97%) of mean distance values within species were less than 2%, though the scale of divergence appears to vary extensively between species: all 10 specimens of *Goneplax rhomboides* (Linnaeus, 1758) (Brachyura: Goneplacidae) share the same haplotype, and *Cherax preissii* (Erichson, 1846) (Astacidea: Parastacidae) exhibited the highest mean intraspecific value of 2.61±0.193 K2P distance.

### GC content divergence assessment

Our second line of inquiry involved assessing the GC content in diverse lineages as a measure of nucleotide diversity. The frequency of the occurrence of GC-content can be a useful metric for understanding species diversity and evolutionary processes [58]. Nucleotide composition varied greatly, ranging from 30.8% to 49.4% of GC content (Table 4). In all cases, GC content decreased from the first to the third codon position with mean values of 50.90%, 42.92% to 21.73% respectively. The pattern of variance (standard error) confirmed that the highest range in GC content was observed in the third codon position: the second position displayed the least variation (Table 4). The proportion of nucleotides throughout 1572 sequences in case B_R_ was T = 34.7%, C = 20.1%, A = 26.9%, and G = 18.3%, respectively. Nucleotide bias did not occur at the first codon position (1^st^), though at the second codon position (2^nd^ ), there was marked bias in T and C, and favouring A against C at the third codon position (3^rd^ ). The average frequency (R) of transitional (A/C and C/T) and transversional (A/T; A/C; C/G; G/T) rates are: COI barcode region R = 1.02; for 1^st^ codon R = 2.7, for 2^nd^ codon R = 1 for and for 3^rd^ codon position R = 0.8.

**Table 4:** Variation of GC content in the COI barcode region and codon position among the Decapoda and from 11 selected families.

				Codon position		
Taxon	Min.	Mean	Max.	1st	2nd	3rd
**Order**						
**Decapoda**	30.8	39.39±0.085	49.4	50.90±0.062	42.92±0.021	21.73±0.213
(1572 seq, 528 sp, 213 gen, 67 fam)						
**Families**						
**Atyidae**	35.30	42.40±0.346	47.00	52.76±0.170	43.51±0.099	30.83±0.958
(59 seq, 16 sp, 9 gen)						
**Chirostylidae**	31.50	33.05±0.139	35.60	49.54±0.129	42.78±0.508	6.81±0.328
(66 seq, 13 sp, 1 gen)						
**Crangonidae**	34.60	39.33±0.361	47.60	50.46±0.227	43.17±0.083	24.36±0.904
(58 seq, 16 sp, 7gen)						
**Galatheidae**	33.20	37.24±0.169	45.50	50.56±0.148	43.03±0.037	18.11±0.493
(220 seq, 84 sp, 10 gen)						
**Lithodidae**	34.40	36.31±0.226	40.40	48.82±0.107	43.76±0.097	16.36±0.621
(52 seq, 12 sp, 6 gen)						
**Majidae**	30.80	35.37±0.253	38.70	48.29±0.244	42.22±0.066	15.61±0.574
(67 seq, 24 sp, 14 gen)						
**Paguridae**	32.40	36.34±0.197	41.00	49.80±0.262	43.07±0.051	16.14±0.608
(57 seq, 24 sp, 14 gen)						
**Palaemonidae**	36.40	41.01±0.404	48.60	52.70±0.311	43.83±0.094	26.51±0.884
(87 seq, 32 sp, 5 gen)						
**Pandalidae**	35.20	41.93±0.274	49.40	51.39±0.163	43.32±0.066	31.06±0.740
(74 seq, 19 sp, 5 gen)						
**Parastacidae**	37.30	40.39±0.186	48.60	51.25±0.110	43.34±0.063	26.53±0.550
(98 seq, 43 sp, 8 gen)						
**Portunidae**	31.90	38.31±0.334	44.20	50.44±0.243	41.87±0.056	22.60±0.866
(90 seq, 22 sp, 10 gen)						

**Figure 4:**
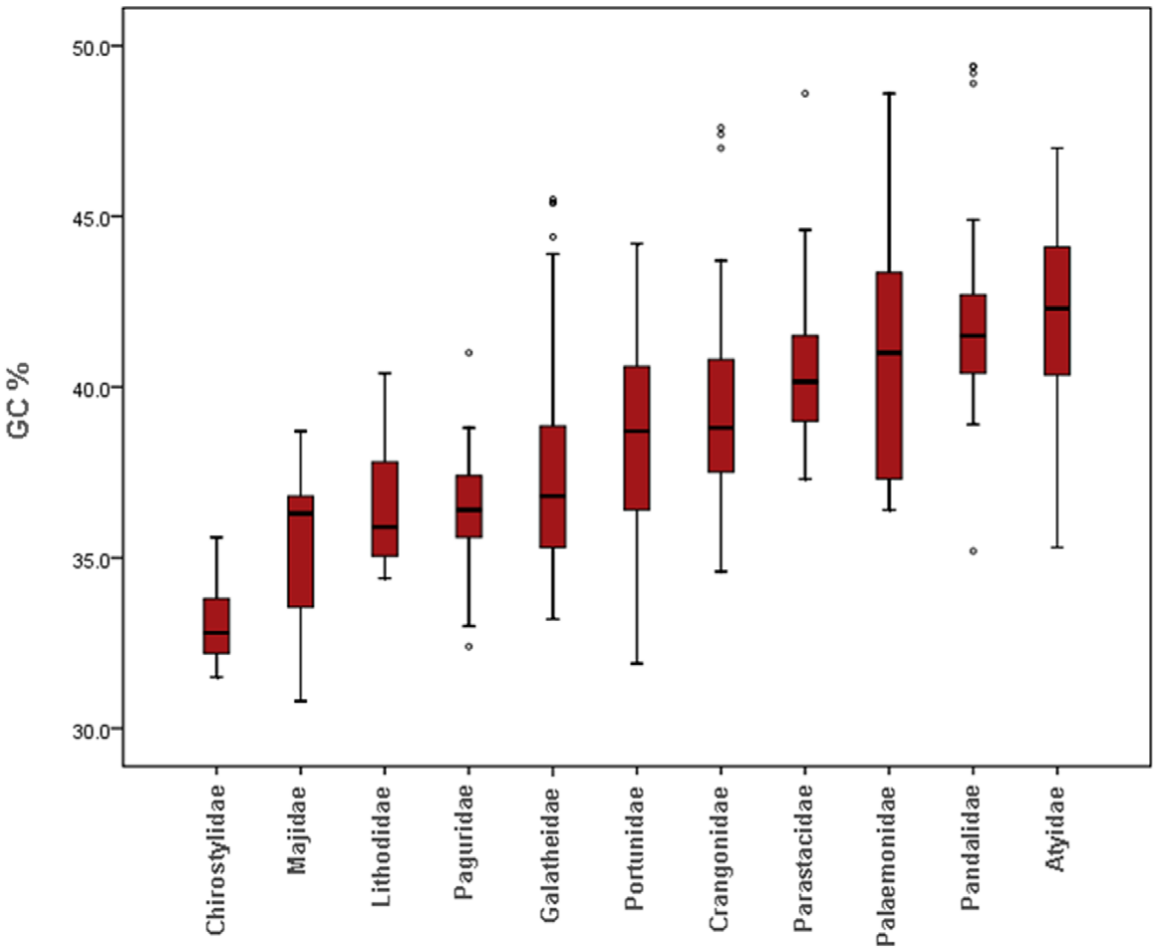
Boxplot distribution of ascending GC content (%) from 11 selected families. The number of sequences, species, and genera per family is indicated in Table 3 and statistic values in Table 4.

Our observations reveal considerable variation in the range of GC values within and among decapod families (Figure 4). Such variation leads to a zone of overlap covering even the most GC rich values in Pandalidae (49.4 % GC), and the lowest values in Chirostylidae (35.6% GC). The highest GC% content was observed in the Atyidae with a mean value of 42.40±0.3465, and the lowest in Chirostylidae of 33.05±0.1392 (Figure 4), mostly reflecting a marked difference at the third codon base with 30.83±0.9582 and 6.811±0.3289. All 11 families examined were significantly different (*p*<0.05), but with considerable overlap (Figure 4). No sample bias effect was observed (*p>*0.05), except for the Palaemonidae (*p<*0.05), which also exhibited the highest standard error variation (SE) value.

## Discussion

The COI gene appears to be an informative molecular marker at several taxonomic scales, but particularly at the species level. Our analysis shows a general increase in the molecular divergence of COI with taxonomic rank, a trend that suggests that morphological taxonomy is roughly in agreement with DNA evolution. Yet, this relationship is not entirely consistent, and the distribution of divergences at different taxonomic scales sometimes overlaps. The COI gene tree was used in this study to present our results and to allow comparison with previously defined species groups within decapods. However other genes and phylogenetic methods are required to evaluate the evolution information contained in the barcode region of COI [59]. It is worthwhile emphasizing that it was not within the scope here to generate new insights into decapods species evolutionary relationships, but rather to analyse patterns of COI variability among decapods.

### New data acquisition

Our data further supports the validity of DNA barcoding for species identification in marine decapods. The ratio of within species to between species variation (21X) was much higher than the threshold (10X) proposed by Hebert *et al*., [37] as a potential species' boundary. Therefore, assigning specimens to species was usually straightforward with no overlap between within species – and between species distance (95% of the cases).

### COI divergence assessment

It has been discussed whether COI barcoding sequence variation will defer yield new insights into the evolutionary relationships among different taxonomic metazoan groups, once complete barcode data are available. Whereas each family apparently coincides with the expected molecular hierarchy, the scale of divergence at each taxonomic level appears to vary extensively between and within families.

The highest values of F (Figure 3) belong to families of infraorder Caridea (Atyidae, Pandalidae, Palaemonidae, Crangonidae), representative of the currently recognized natant lineages of the suborder Pleocymata [60]. Such high values of genetic distance reflect possibly the remarkable range of adaptation and biological diversity within the infraorder Caridea [46], [61]–[63]. Many caridean families inhabit both shallow and deep water marine environments [62], hythrothermal vents [64], freshwater lakes and mountain streams [63], caves [65], and commonly establish temporary or lifelong associations with other taxa [66]–[68]. The phylogeny of the infraorder Caridea based on mitochondrial and nuclear genes has suggested that the Caridea is monophyletic [61], underpinned by a possible radiation in the Triassic period [60]. Apparent polyphyletic and paraphyletic compositions of some Caridean families have, however, been reported by morphological and molecular studies [61]. Also multi-locus genes, including both mitochondrial and nuclear genomes and additional taxa, will need to be analysed to provide informative characters to resolve the phylogeny among Caridean groups.

The economically important Lithodidae and Pandalidae exhibit markedly contrasting patterns of intrafamily divergence (Figure 3). The typically cold-water Lithodidae king crab comprises weakly divergent species, suggesting either that the family represents an extreme situation of rapid morphological diversification, and/or slow molecular evolution, reflecting a slow metabolism found in organisms that inhabit cold environments [69], [70], or possessing larger body sizes [71], [72] or both [73]–[75]. Moreover, distribution, and therefore opportunities for population differentiation, in these groups remains constrained by the stressful effects of temperature extremes on early life-history stages [76]. However, the phylogeny of the family Lithodidae is controversial [77], [78], and molecular and adult and larval morphological data remain equivocal [79], [80].

The Oregoniidae also exhibits very low mean divergences within taxa (S = 0.66%; G = 5.56%; F = 12.96%), here represented by five deep water species from two genera. Nucleotide substitution rate is the ultimate source of genetic variation and it is the substrate for molecular evolution. The metabolic rate hypothesis [81], [82] has been proposed to explain mtDNA substitution rate variations in animals. Correlation between metabolic rate and nucleotide substitution may be mediated by (i) the mutagenic effects of oxygen radicals that are abundant by-products of aerobic respiration, and (ii) increased rates of DNA synthesis and nucleotide replacement in organisms with higher metabolic rates [82]. The general hypothesis assumes that deep-sea animals exhibit hypo-metabolism [83]–[85], which is characterised by abnormally low level metabolic rates. The theory holds that limited light with depth reduces visual predation pressure and selects for reduced locomotory ability and metabolic capacity [86]. Although this theory applies predominantly to pelagic animals, deep-sea benthic animals (including crustaceans) exhibit metabolic rates also typically an order of magnitude lower than their shallow-water counterparts [70], [86]. While this phenomenon in deep-sea benthic crustaceans may simply be a function of very low temperatures at depth in areas of steep thermal gradients [86], reduced metabolic rates observed in deep-sea benthic crustaceans may still be ecologically relevant to their rate of molecular evolution.

The Pandalidae is one of the most species-rich families due to extensive diversification in the genus *Plesionika*. Our data set showed the highest nucleotide divergences within the genus, represented by the genera *Plesionika*, *Pandalus*, *Pandalopsis* and the monospecific *Dichelopandalus* and *Stylopandalus*. The genus *Pandalus* (Leach, 1814) is retained as a possible paraphyletic group [87], and the phylogeny of *Pandalopsis* remains to be described. The phylogenetic relationship between members of the genus *Plesionika* is still to be established and in spite of recent taxonomic revisions [88]–[90], our data endorse the need for additional effort.

### Taxonomic classification

One of the factors that may bias our divergence assessment is the possibility of incorrect or uncertain taxonomic classification.The COI barcodes grouped together the two spider crab specimens (0% distance) *Macropodia longipes* and *M. tenuirostris* (Leach, 1814). Such genetic similarity, if generally supported, emphasizes the idea that these two species should be considered as one based on similar morphological characteristics of adults and larval stages [91]–[95]. Combined data presented herein suggests that *M. longipes* is in fact a synonym of *M. tenuirostris*.

Low divergence levels were observed (0.065%) also between *Pachygrapsus maurus* and *P. marmoratus*. *Pachygrapsus marmoratus* can be distinguished from related species *P. maurus* by the presence of two lateral post-orbital teeth, whereas *P. maurus* possesses one [96]. *Pachygrapsus marmoratus* and *P. maurus* are considered sister species and are genetically clearly distinct to other species of the genus [97]. Ecologically, these two species share the some rocky intertidal area and were collected from Flores Island in the Azores Archipelago. Our data might indicate hybridization or a misidentification. In our study *P. maurus* was represented by two juvenile specimens, and in spite of the evident differences in adult morphology [98] the diagnostic features can be hard to distinguish in juvenile specimens. Further molecular (e.g. AFLP or microsatellites [99]) and morphological analyses should be combined to identify species and between species hybrids within the *Pachygrapsus* species.

### Cryptic and young species

For decapods, COI resolves relationships among the more closely related species within genus, and can be used to address the question of whether species groups based on morphological, ecological and biogeographical characters represent evolutionary lineages. The described levels of intraspecific variation must be considered preliminary, since several species were characterized based on only up to ten specimens – sufficient for a valid barcode, but not sufficient to accurately capture genetic diversity of the species. However pairwise sequence differences derived from 10 specimens per species reflected differences in the range of diversity. DNA sequences for additional specimens collected across the geographic ranges of additional species are needed to test and validate this result. In some cases, higher levels of intraspecific variation may reflect underlying population structure. For example, the freshwater crayfish *Cherax preissii* (Erichson, 1846) (Astacidea: Parastacidae) showed highest divergence values with a maximum of 4.45% genetic distance (0.5 patristic distance) between two main populations from the North and South of Australia. A recent systematic study of the genus *Cherax* suggested that the taxonomy of *C. preissii* should be re-examined [88], even if the diversity between Australian populations reveals evidence of contemporary, but not ongoing, gene flow during pluvial Pleistocene periods [89]. However, extensive cryptic species have been documented in freshwater crayfish taxa, concurring with the increased discovery of diversification in freshwater taxa [66], [100]–[104]. Another example is the species *Macrobrachium nipponense* (De Haan, 1849) (Caridea:Palaemonidae) with a maximum distance of 4.15% (0.4 patristic distance). The genus *Macrobrachium* has more than 100 species described, distributed exclusively in freshwater and brackish habitats (except *M. intermedium* (Stimpson, 1860)) [105], [106]. The species of this genus exhibits significant intra-population and intra-individual variation in egg size [107] and larval characters [108]. *Macrobrachium nipponense* exhibits high tolerance of variation in water parameters, having the ability to change in three generations to full freshwater [109], and together with its popularity in the aquarium trade, renders it an effective invasive species [110], [111]. Taxonomic complexity is associated with morphological plasticity of taxonomically important (e.g.,the rostrum and/or the second periopod) changes in relation to growth [112] and environmental variation [113]. The morphological characters are extremely conservative and molecular systematic data from the genus *Macrobrachium* suggests that the uses of traditional morphological characters and molecular data are essential to diagnose accurately natural species groups [100].

It seems likely that cryptic species will be discovered among geographically widespread decapods species. Here, two shrimp species *Palaemon elegans* (Rathke, 1837) and *Pasiphaea tarda* (Kreyer, 1845) from the Northeast Atlantic Ocean showed non-monophyletic patterns when compared with their con-specifics from other oceanographic regions. For the first example, *P. elegans*, the mean distance within species was 5.296% (0.530 patristic distances). Previously, three morphological types for the cosmopolitan species *P. elegans*, have been suggested (see for review [93]), supported by high allozymic divergence within the Mediterranean Sea [114]. This species is adapted to extremely variable salinities, temperatures and oxygen [115], [116]. A surprisingly complex population structure within *P. elegans* has been recently discovered comprising three haplogroups [117] [142] from: Atlantic and Alboran Sea, Mediterranean Sea and the Black Sea, Caspian and Baltic Sea. The Baltic Sea population revealed high levels of nucleotide divergence suggesting the existence of a cryptic species that originated in the late Miocene period when ancestral Baltic populations of *P. elegans* were isolated from Atlantic populations [117] [142]. It is likely, however, that the occurrence of this species in the Baltic Sea represents an introduced invasive species rather than an effect of natural expansion [117], [118]. Based on such a scenario, it is possible that specimens from the Baltic Sea from Costa *et al*. [35] represent a cryptic species, or that hybridization is taking place between *P.elegans* and *P. intermedius* [117]. Interestingly we had only found difference in one amino acid positions between Northeast Atlantic Ocean and Baltic populations. Although this difference cannot be considered as indicating species separation, it does suggest the need for a re-examination of specimens [119].

Most marine species the preponderance of pelagic larval stages and the absence of obvious distribution barriers suggests a high level of gene flow with populations predicted to be genetically homogeneous[120]. However high levels of genetic differentiation between populations over small spatial scales were described [121], [122] suggesting that marine ecosystems may not be as interconnected as they seem [123], [124]. *Pasiphae tarda* revealed a maximum intraspecific distance of 4.913 % (0.509 patristic distance). In relation to the data presented here for the cosmopolitan species *P. tarda,* it is possible that limited larval dispersal/gene flow is associated with deep genetic breaks between populations between the North Pacific Ocean and the North Atlantic. Several comparative phylogeography studies in marine taxa, including corals, decapods and bryozoans, have suggested various ages of the genetic discontinuities, ranging from the Miocene to the Pliocene during episodic marine regressions [125]–[129]. These authors showed concordance of genetic structure across multiple taxa combined with temporal discordance suggesting that regional genetic structures have arisen from common physical processes operating over extended time periods. The presence of intraspecific genetic structure, as well as deeply divergent lineages, strongly suggests that such overarching processes promote lineage diversification [125]–[129].

The presence of intraspecific genetic structure is furthermore supported by high amino acid diversity within species showing variation in four amino acid positions between Pacific and Atlantic populations.

Whether *C. preissii* , *M. nipponense*, *P. elegans* and *P. tarda* exhibit taxonomically significant geographic variation and/ or comprise cryptic species should be reviewed with additional morphological, as well as population genetic and molecular systematic studies with multi-locus genes. Based on the taxonomic incongruence identified here, such approaches can explore further the levels of cryptic speciation and reproductive isolation across putative species [130].

The utility of COI as a tool for rapid identification depends on the genetic variation among species exceeds intraspecific variation to such an extent that a clear “bacording gap” exits. However, the gap might be absent in younger species (incomplete lineage sorting) and species with hybrid zones because of the insufficient variation to be determined as distinctly different using only barcodes [36], [131]. Our data further support the incomplete lineage sorting of the genus *Hyas* reported between *H. araneus* (Linnaeus, 1758) and *H. coarctatus* (Leach, 1815), [132]. These species are morphologically distinct from larval stages to adulthood [133], indicating that misidentification is highly unlikely, and incomplete lineage sorting is more plausible. We found low levels of divergence (0.778%) between one specimen of *Hyas lyratus* (Dana, 1851) from Costa *et al*., [35] and *H. coarctatus* supporting the recent evolution of the genus. However additional analyses among nuclear rDNA genes will be necessary to confirm the hypothesis of recent evolution and identification or delineation at to species of the genus *Hyas*.

### Nuclear mitochondrial pseudogenes (numts)

COI has been the preference for species identification/delineation due the traditionally accepted advantages of mtDNA. However, it is also well recognised that analysis of mtDNA sequence variation can be distorted by the inclusion of nuclear mitochondrial pseudogenes (numts). Because the DNA barcoding initiative attempts to barcode all life forms, the potential impact of numts issue cannot be ignored [134]–[136]. Numts are non-functional copies of mtDNA in the nucleus that have been found in major clades of eukaryotic organisms, e.g., arthropods [134], [137], crustaceans [136] and decapods [135], [138], [139]. Their proportion varies greatly depending on the organism, life style, and on the genome properties (i.e., rates duplication, mutation, deletion, and retrotransposition, see [140], [141] for review). Numt sequence can be highly divergent from the orthogous COI sequences. Additionally, high genetic divergences are used to indicate possible new species that may be nested within species complexes. Buhay [136] reported a list of potential cases of numts in Crustacea when she found reading frame problems without the occurrence of stop codons. Even though the proportion of adenine – thymine (numts have a significantly lower AT% compared with the orthologous mtDNA [134]) did not differ between specimens, there is increasing concern about the potential overestimation of species richness [134] by inclusion of numts. Here, we have discussed the occurrence of high nucleotide divergences within species, e.g., *Cherax preissii* [104]. As an example here, we cannot ignore the possibility of dealing with numts sequences even if our quality controls failed to detect them (see Methods). Also other studies showed that mitochondrial cytrochrome *b* gene fragments in the freshwater crayfish, *Cherax destructor* (Clark 1936) had numts [139]. They reported of four closely related crayfish species (*Orconectes* spp.) the presence of numts of the COI gene and how barcoding methods would incorrectly infer single individuals belonging to multiple, unique species [134]. Moreover, we found high amino acid diversity among *C. preissii* species showing difference from three amino acid positions. More than two amino acid intraspecific changes could represent a radical change [142] at highly conserved COI gene and as they are likely caused by sequencing error [119]. Especially when numts were already reported for this genus or even for members of the family Parastacidae, it is worthwhile for the scientific community to analyse additional morphological characters and molecular markers other than mitochondrial genes. Characterization of numts is important to understand genome dynamics and evolution, and their significant increases when several genomes of related organisms can be compared. It is thereby important to ensure that numts sequences are not discarded, but recognized, labelled, and submitted as such [136], [138].

### GC content divergence assessment

For decapods, substantially more nucleotide changes were observed at the 3^rd^ codon position than the 1^st^, and more at the 1^st^ than the 2^nd^: the SE of the GC % of the 3^rd^, 1^st^ and 2^nd^ bases of Decapoda were 0.213, 0.062 and 0.021, respectively (Table 4). Such values indicate the fact that most synonymous mutations occur at the 3^rd^ position, with a few at the 1^st^ position and none at the 2^nd^ as also observed in Australian fish [143].

Despite the commonly held view that invertebrate mitochondria are AT-rich, while chordate mitochondria are GC-rich [12], [29], [144] with a mean value up to 45% GC content [29], our observations reveal considerable variation in the range of GC values (31–50% GC) within decapods (Figure 4), with a mean value of 38%. Similar values have been reported in independent Decapoda COI assessments, but also for total mtDNA diversity within the order [29]. Appraising a wider taxonomic breadth, Clare *et al.*, [29] also detected large shifts in GC content (up to 8%) even at the generic level in the Insecta, highlighting that heterogeneity in mtDNA GC content is not restricted to our current observations.

The wide range of GC content in some families in our analysis is intriguing, though observations here must be treated cautiously as most sequences originated from GenBank, a source where sequencing error and misidentifications have been well documented [136]. Nevertheless, the wide range among families was largely due to 3^rd^ codon positions as also observed in fish species [143]. Several explanations for genome shifts in nucleotide composition exist, which can be categorized into theories of mutational bias (observation that purine to purine or pyrimidine to pyrimidine changes -transitional- occur with greater frequency than purine to pyrimidine or vice versa - transversional [145] and natural selection [144]). There remains a strong interest in exploring the environmental context of such shifts, including fluctuations in temperature, salinity, pressure [75], [146]–[152], and biological factors such as population size, generation time, body size, larval dispersal, mutation rate and parasite behaviour [74], [153]–[160]. It is important to underline that the families with higher GC values belong to the oldest Pleocymata lineage Caridea [60]. It is known that DNA sequences with similar GC content may be grouped together if phylogenetic analysis is performed on DNA sequences [161]. GC-rich DNA is assumed to produce a more heat-stable helix [162] and thus can be selectively advantageous in animals with high metabolic regulation induced by environmental drivers such as light, temperature, salinity, oxygen, and pH. Recently a study [145] showed the existence of a strong positive correlation between hydrophobicity and genomic GC content in prokaryotic organisms. Although the importance of hydrophobicity on the stability of proteins has been observed in most of the protein families [163], GC increment may be related to the structural and functional changes of the encoded proteins [145] in Caridea, suggesting that natural selection is the main force influencing mutation patterns.

### Sample size and geographical coverage for species diversity assessment

Early in the DNA barcode initiative the question of how many specimens are needed to create a reliable reference for specimen identification and diversity assessment remained largely unresolved. A sample size of 12 individuals per species was proposed by [164], but it has been correctly asserted that a reference sequence sample for all species seems pointless without taking the evolutionary characteristics of each species into account [165]. Zang *et al.*, [165] showed that there is no significant correlation between samples size and the percentage of the total number of haplotypes observed, and the effort of finding new haplotypes varies considerably over different species/populations. In our data the pattern of diversity found among species is very diverse, but it remains unclear how representative it is as an estimate of genetic/variation diversity based on a sample of 10 individuals. As an example we have the species *G. rhomboides* represented by 7 individuals from the Portuguese west coast and three from Great Britain sharing a unique haplotype. Such data suggest that we should have better randomized sampling from the whole geographical distribution of a species in DNA barcoding projects to better encompass the diversity of the species. Nevertheless, the trends disclosed, together with the high levels of concordance overall between previous indications of taxonomic anomalies and links to coarse environmental features, does suggest that data presented here are broadly representative of contemporary biodiversity patterns. Indeed, examination of diversity at the COI region yields an informative framework to identify and explore priority issues, demanding in turn a fully integrative approach utilising additional molecular, distributional and ecological information.

### Conclusions

Although our study is limited to decapods, and the sampling is limited to a small proportion of the entire order (5.4% of the 17635 extant species described), it is unlikely that the general patterns observed have been biased by our sampling or taxonomic coverage. Here with our range of molecular data we have contributed to the assessment of decapods biodiversity in several ways, including: revealing putative cryptic species (e.g., *Palaemon elegans*); assigning correct species names of taxa with different life history stages (*Pachygrapsus marmoratus*); confirming the existence of the synonymy names (*Macropodia tenuirostris*); facilitatating a rapid assessment of taxon diversity in groups that have until now received limited morphological and systematic examination (*Macropodia*), and we also flag taxonomic groups (Caridea; Lithodidae and Pandalidae) with unusual nucleotide composition or evolutionary rates. Intraspecific genetic diversity has a fundamental role in delimiting species boundaries. The burgeoning record of barcode records, in conjunction with additional ecological and molecular approaches, is likely to enhance understanding of the history and evolutionary trajectory of decapod species. It has become essential that species are accurately delineated, cryptic species are identified and/or conservation units are proposed on the basis of sound phylogenetic and phylogeographic variation in space and time. Efforts to conserve biodiversity should work to preserve both existing biodiversity as well as the evolutionary processes shaping genetic diversity, the core determinant evolutionary potential for adaptation to changing environments.

## Materials and Methods

### Data sampling

We collected 516 decapods specimens from the North East of the Atlantic, the Gulf of Cadiz and the Mediterranean Sea between 2005 and 2008. The specimens encompassed 101 species in 74 genera from 42 families of the order Decapoda. Deep-water specimens were collected by the National Institute of Biological Resources (INRB-IPIMAR) with nets and by the IOC-UNESCO Training through Research programme and the EU funded project Hotspot Ecosystem Research on the Margins of European Seas (HERMES) with two dredges and three box-cores. Littoral specimens were collected at low tide using dip nets, baited traps and scuba diving. Samples were stored in 70% ethanol (2001–2005) and in 100% ethanol (2006–2008). Morphological identifications were undertaken and confirmed by taxonomists. Scientific names followed the Integrated Taxonomic Information System (www.itis.gov). In most cases, the whole specimen was stored as a morphological voucher for future reference (Table S1). For some large decapod species, only tissue (legs or abdominal muscle) was obtained for barcoding and the samples were stored as tissue vouchers, accompanied by photographs taken prior to DNA extraction. All details regarding taxonomy, vouchers and collection sites with geographical coordinates can be found in the Barcode of Life Data System website (BOLD, www.barcodinglife.org) under two campaigns, Marine Life (MarBOL) and Portugal – Aquatic Life (Table 1). In order to ensure adequate geographical coverage, multiple specimens (at least two per site) from different geographical areas of target species were examined.

Total genomic DNA was extracted from small amounts of tissue (1 mm^3^ muscle tissue or whole legs for small specimens) using the Chelex dry release [166] or QIAGEN DNeasy tissue extraction kits (QIAGEN) for older or less well preserved samples. Prior to DNA extraction, the sample was washed overnight in 50 µl of QIAGEN Buffer AE (10 mM Tris-Cl; 0.5 mM EDTA; pH 9.0) in order to rehydrate the tissue. For the Chelex dry release extraction method tissue samples were added to 120 µl of a 10∶2 mixture of Chelex buffer with Proteinase K (Sigma), incubated at 55°C for 8–12 hours and subsequently heated to 95°C for 20 minutes. The barcode region was amplified with alternative sets of primers depending on PCR reaction success. The primers used with forward direction were LCOI490 [167], CrustDF1 [132], CrustF1 [35], CrustF2 [35], and COL6 [138] and with the reverse primers HCO2198 [167]; CrustDR1 [132]; CrustR2 (5′- GGT AGA ATT AGA ATA TAC ACT T – 3′, designed within the context of the BOLD- FCDPA project), COH6 [138]. A cocktail of primers with M13 tails [168] was used with two forward and two reverse primers LCOI490; CrustF1; HCO2198; and CrustR2. All PCRs were performed in a 25 µl volume containing 1 X PCR buffer, 3 mM MgCl2, 0.1–0.2 mM dNTP, 1U TAQ polymerase (Promega), 5–10 pmol of each primer, and 2–10 ng of DNA template. The thermal cycling conditions consisted of 94°C for 60 s; 35–40 cycles of 94°C for 30 s, 48–56°C for 90 s, and 72°C for 60 s; followed by a final extension of 72°C for 5 mins. Alternative thermal cycling conditions was consisted of 94°C for 60 s; 5 cycles of 94°C for 30 s, 45°C for 90 s, and 72°C for 60 s; 35 cycles of 94°C for 30 s, 50–56°C for 90 s, and 72°C for 60 s; followed by a final extension of 72°C for 5 mins. The thermal cycling was identical for all primer except the CrustF2/HCO primer set, which was as follows: one cycle of 94°C for 60 s; 35 cycles of 94°C for 30 s, 42°C for 90 s, and 72°C for 60 s; followed by a final extension of 5 min at 72°C. PCR products were visualized on precast 1% agarose gels using the E-gel 96 system (Invitrogen). Prior to sequencing 15 µl PCR products were cleaned with 1U shrimp alkaline phophatase (Promega) to dephosphorylate residual deoxynucleotides and 0.5 U Exonuclease I (Promega) to degrade excess primers [169]. The purification thermal conditions consisted of 37°C for 45 min and 80°C for 15 min. Bidirectional sequencing was performed using BigDye Termation chemistry on an Applied Biosystems® 3730 sequencer by Macrogen Inc. (www.macrogen.com, South Korea). Sequences were manually checked for ambiguities and assembled in CodonCode Aligner version 1.3.0 (http://www.codoncode.com/). Sequences were aligned using CLUSTAL W [170] implemented in MEGA4 [171] and the amino acid translation was examined to ensure that no gaps or stop codons were present in the alignment. BLAST searches were performed for all sequences via interrogating GenBank's online nucleotide database using the megablast algorithm.

### Genbank data set

To provide a comprehensive sister-species coverage and survey of intraspecific variation, our data set was complemented by COI sequences from GenBank, as available on 4^th^ June 2009. Additional sequences were included from the Barcode of Life Data Systems website (http://www.barcdoinglife.org/, as accessed on 4^th^ June 2009). The BOLD platform allows us in our Project List page to have access not just to our full list of personal projects, but also all publicly accessible projects on BOLD, e.g., GenBank Animals (COI) and MarBOL compains. The BOLD system archives sequences located in COI barcode region from samples identified only to genus and species level being less than half of the COI entries in NCBI GenBank database. Sequences were omitted in our study if they were not allocated to a species, were from taxa with multiple denominations or taxonomic ranks, and suspected of being derived from misidentified, mislabelled species or putative pseudogenes (when found intraspecific distances >10%, aberrant nucleotide composition, unusually long branches in our NJ tree and nonsensical systematic relationships [134], [136]), exhibited stop codons or indels, were less than 500 bp in length within the COI barcode region and finally sequences that were not reported in scientific journals to avoid potential misidentifications that could possibly be derived from GenBank [172], we submitted these to a rigorous quality control. From public projects [57] we downloaded 5052 comprising 856 species from 249 genera and 83 families only 3187 COI barcode region sequences were selected from 520 species, 178 genera and 53 families with sufficient length and quality according to our stringent criteria.

### Combined data set: sequence selection and data validation

Two main factors may bias divergence assessments. First, disequilibrium in the representation of some taxa could skew divergence distributions. Here we standardized taxon comparisons to maximum of 10 individuals per species [173] randomly selected reducing to 1906 sequences from 603 species, 225 genera and 68 families were included in the total data analyses. To test how patterns of genetic divergence at COI correspond to morphological species concepts, species diversity was estimated based on the similarity and clustering pattern in their COI barcodes independent of taxonomic assignments. A threshold of 2% sequence divergence was employed to draw boundaries for barcode haplotype clusters. This arbitrary threshold was selected based on the observation that intraspecific divergences observed in a variety of groups rarely exceed this value [12]–[16].

Secondly, the taxonomic classification may be incorrect or uncertain. Most common problems will result from cryptic species, and paraphyletic or polyphyletic taxa [10], [174]. All sequences were aligned and a Neighbour Joining tree produced using BOLD platform. We identified, in this tree, all sequences clustering far from their known taxonomic or phylogenetic position, and removed the non-monophyletic, putative cryptic species and congeneric species with distance values lower than 2% evaluated from the literature. After such selective removal, we proceeded to analyse 1572 sequences from 528 species, 213 genera, and 67 families. Additionally we tested the possible artefact attributable to biased species representation by computing within mean species divergence and the influence of presumably non-monophyletic taxa on the divergence distribution. An assumption-free statistical test was proposed by Lefébure *et al.*, [10] to directly measure the overlap between raw data (highly represented taxa have more impact than weakly represented ones) and a second set of data where each taxa was given the same weight by computing mean divergence through distance values. Comparing the frequency of intraspecific distances values (<3%) between the raw data and the mean data will indicate whether or not the divergence assessment is a result of a strong disequilibrium in the representation of some taxa. The divergence distribution was tested within species diversity between the initially dataset with 1906 sequences (case A) and the validate data set with 1572 sequences (case B). To obtain the first statistical indication of the overlap between divergence distributions, Mann-Whitney U Test were performed (Figure 1) among [10]: raw data A_R_ vs B_R_, mean data A_M_ vs B_M_; and between different data A_R_ vs A_M_ and B_R_ vs B_M_ with the SPSS software version 16.0.2 [175].

### Decapoda diversity assessment

The diversity assessments for the decapods and for the most represented families were analysed from the data set with 1572 sequences from 528 species, 213 genera, and 67 families (B_R_). For statistical purposes only, families containing at least 50 sequences [10], [174] with more than 5 species were compared [10]. Nucleotide divergences of COI and variation in GC content were analysed between the 11 most representative families (Table 2).

The K2P has become the metric most widely used in barcoding studies and is deployed here. Genetic distances between specimens were calculated for each intraspecies (S), intragenus (G) and intrafamily (F) with the 'Distance Summary' command implemented by BOLD. Although distance distributions within families are not independent from each other, we performed the Kruskal–Wallis one-way analysis of variance between S, G, and F distributions to obtain a first statistical indication of the overlap between divergence distributions with GenStat [176].

In order to investigate the sensitivity of results to variations in matrices distances methods, Patristic distances were computed using the program PATRISTIC [177].

Our second line of investigation examined the diversity in GC content across multiple taxonomic groups. To ensure homology with the BOLD data (because the sequences are heterogeneous in length), all sequences (1572) were trimmed to 500 bases and GC content and nucleotide composition were calculated for 11 families using MEGA 4 [171].

## Supporting Information

Figure S1 **Taxon ID Tree of Decapoda generated by BOLD.** Neighbour Joining tree (Kimura 2-parameter, uniform rates among sites, pairwise deletion) combining COI data from public BOLD projects and present study. A total number of 1906 sequences from 603 species, 225 genera and 71 families were used.
(PDF)

Table S1 **Novel COI decapod barcodes generated by the present study.**
(XLS)

Table S2 **Accession numbers for the sequences used in this study.** Specimens' list of 1906 COI sequences from 603 species, 225 genera and 71 families.
(XLS)

Table S3 **Accession numbers for the sequences removed from the decapod diversity assessment analysis.** Specimens' list of 340 COI sequences from 79 species, 30 genera and 19 families.
(XLS)

Table S4 **Accession numbers for the sequences used for assessment of decapod diversity.** Specimens' list of 1572 COI sequences from 528 species, 213 genera and 67 families.
(XLS)

## References

[1] Wilson EO (2003). On global biodiversity estimates.. Paleobiology.

[2] Blaxter M (2003). Molecular systematics - Counting angels with DNA.. Nature.

[3] Wilson EO (2003). Biodiversity in the information age.. Issues in Science and Technology.

[4] Wilson EO (2003). The encyclopedia of life.. Trends in Ecology & Evolution.

[5] Butchart SHM, Walpole M, Collen B, van Strien A, Scharlemann JPW (2010). Global Biodiversity: Indicators of Recent Declines.. Science.

[6] Minelli A (2003). The status of taxonomic literature.. Trends in Ecology & Evolution.

[7] Jong Gd (2004). Evolution of phenotypic plasticity: patterns of plasticity and the emergence of ecotypes.. New Phytologist.

[8] Sánchez JA, Aguilar C, Dorado D, Manrique N (2007). Phenotypic plasticity and morphological integration in a marine modular invertebrate.. Bmc Evolutionary Biology.

[9] Pérez-Barros P, d'Amato ME, Guzmán NV, Lovrich GA (2008). Taxonomic status of two South American sympatric squat lobsters, Munida gregaria and Munida subrugosa (Crustacea: Decapoda: Galatheidae), challenged by DNA sequence information.. Biological Journal of the Linnean Society.

[10] Lefébure T, Douady CJ, Gouy M, Gibert J (2006). Relationship between morphological taxonomy and molecular divergence within Crustacea: Proposal of a molecular threshold to help species delimitation.. Molecular Phylogenetics and Evolution.

[11] Costa FO, Carvalho GR (2007). The Barcode of Life Initiative: synopsis and prospective societal impacts of DNA barcoding of Fish.. Genomics, Society and Policy.

[12] Hebert PDN, Cywinska A, Ball SL, DeWaard JR (2003). Biological identifications through DNA barcodes.. Proceedings of the Royal Society of London Series B-Biological Sciences.

[13] Gregory TR (2005). DNA barcoding does not compete with taxonomy.. Nature.

[14] Ebach MC, Holdrege C (2005). DNA barcoding is no substitute for taxonomy.. Nature.

[15] Schindel DE, Miller SE (2005). DNA barcoding a useful tool for taxonomists.. Nature.

[16] Miller SE (2007). DNA barcoding and the renaissance of taxonomy.. Proceedings of the National Academy of Sciences.

[17] Radulovici AE, Archambault P, Dufresne F (2010). DNA Barcodes for Marine Biodiversity: Moving Fast Forward?. Diversity.

[18] Roe AD, Sperling FAH (2007). Patterns of evolution of mitochondrial cytochrome c oxidase I and II DNA and implications for DNA barcoding.. Molecular Phylogenetics and Evolution.

[19] Will KW, Mishler BD, Wheeler QD (2005). The Perils of DNA Barcoding and the Need for Integrative Taxonomy.. Syst Biol.

[20] Meier R, Zhang G, Ali F (2008). The use of mean instead of smallest interspecific distances exaggerates the size of the "Barcoding gap" and leads to misidentification.. Syst Biol.

[21] Hickerson MJ, Meyer CP, Moritz C (2006). DNA Barcoding Will Often Fail to Discover New Animal Species over Broad Parameter Space.. Syst Biol.

[22] Boero F (2010). The Study of Species in the Era of Biodiversity: A Tale of Stupidity.. Diversity.

[23] Hebert PDN, Ratnasingham S, deWaard JR (2003). Barcoding animal life: cytochrome c oxidase subunit 1 divergences among closely related species.. Proceedings of the Royal Society of London Series B-Biological Sciences.

[24] Savolainen V, Cowan RS, Vogler AP, Roderick GK, Lane R (2005). Towards writing the encyclopaedia of life: an introduction to DNA barcoding. Phil. Trans. R. Soc.. B.

[25] Hajibabaei M, Singer GAC, Hebert PDN, Hickey DA (2007). DNA barcoding: how it complements taxonomy, molecular phylogenetics and population genetics.. Trends in Genetics.

[26] Hajibabaei M, Singer GAC, Clare EL, Hebert PDN (2007). Design and applicability of DNA arrays and DNA barcodes in biodiversity monitoring.. BMC Biology.

[27] Meusnier I, Singer GAC, Landry J-F, Hickey DA, Hebert PDN (2008). A universal DNA mini-barcode for biodiversity analysis.. BMC Genomics.

[28] Min XJ, Hickey DA (2007). DNA barcodes provide a quick preview of mitochondrial genome composition.. PLoS ONE.

[29] Clare EL, Kerr KCR, von Konigslow TE, Wilson JJ, Hebert PDN (2008). Diagnosing Mitochondrial DNA Diversity: Applications of a Sentinel Gene Approach.. J Mol Evol.

[30] Hajibabaei M, Janzen DH, Burns JM, Hallwachs W, Hebert PDN (2006). DNA barcodes distinguish species of tropical Lepidoptera.. Proceedings of the National Academy of Sciences of the United States of America.

[31] Puillandre N, Strong EE, Bouchet P, Boissellier MC, Couloux A (2009). Identifying gastropod spawn from DNA barcodes: possible but not yet practicable.. Molecular Ecology Resources.

[32] Rock J, Costa FO, Walker DI, North AW, Hutchinson WF (2008). DNA barcodes of fish of the Scotia Sea, Antarctica indicate priority groups for taxonomic and systematics focus.. Antarctic Science.

[33] Smith MA, Poyarkov NA, Hebert PDN (2008). CO1 DNA barcoding amphibians: take the chance, meet the challenge.. Molecular Ecology Resources.

[34] Summerbell RC, Lévesque CA, Seifert KA, Bovers M, Fell JW (2005). Microcoding: the second step in DNA barcoding.. Phil Trans R Soc B.

[35] Costa FO, deWaard JR, Boutillier J, Ratnasingham S, Dooh RT (2007). Biological identifications through DNA barcodes: the case of the Crustacea.. Canadian Journal of Fisheries and Aquatic Sciences.

[36] Meyer CP, Paulay G (2005). DNA barcoding: Error rates based on comprehensive sampling.. Plos Biology.

[37] Hebert PDN, Stoeckle MY, Zemlak TS, Francis CM (2004). Identification of birds through DNA barcodes.. Plos Biology.

[38] Casiraghi M, Labra M, Ferri E, Galimberti A, De Mattia F (2010). DNA barcoding: a six-question tour to improve users' awareness about the method.. Briefings in Bioinformatics.

[39] Frézal L, Lebloids R (2008). Four years of DNA barcoding: Current advances and prospects.. Infection, Genetics and Evolution.

[40] Bachtrog D, Thornton K, Clark A, Andolfatto P (2006). Extensive introgression of mitochondrial DNA relative to nuclear genes in the Drosophila yakuba species group.. Evolution.

[41] Mallet J (2005). Hybridization as an invasion of the genome.. Trends in Ecology & Evolution.

[42] Mallet J, Willmott K (2007). Taxonomy: renaissance or Tower of Babel?. Trends in Ecology & Evolution.

[43] Oliver L, Beattie AJ (1993). A possible method for the rapid assessment of biodiversity.. Conservation Biology.

[44] Floyd R, Abebe E, Papert A, Black M (2002). Molecular barcodes for soil nematode identification.. Molecular Ecology.

[45] Sémon M, Mouchiroud D, Duret L (2005). Relationship between gene expression and GC-content in mammals: statistical significance and biological relevance.. Human Molecular Genetics.

[46] De Grave S, Pentcheff ND, Ahyong ST, Chan T-Y, Crandall KA (2009). A Classification of Living and Fossil Genera of Decapod Crustaceans.. Raffles Bulletin of Zoology.

[47] Martin JW, Crandall KA, Felder DL (2009). Decapod Crustacean Phylogenetics..

[48] Jones GP, Srinivasan M, Almany G (2007). Population Connectivity and Conservation of Marine Biodiversity.. Oceanography.

[49] Calado R (2006). Marine ornamental species from European waters: a valuable overlooked resource or a future threat for the conservation of marine ecosystems?. Scientia Marina.

[50] Markle DF, Dadswell MJ, Halliday RG (1988). Demersal fish and decapod crustacean fauna of the upper continental slope off Nova Scotia from La Have to Sr. Pierre Banks.. Can J Zool.

[51] Macpherson E, Duarte CM (1991). Bathymetric trends in demersal fish size: is there a general relationship?. Marine Ecology Progress Series.

[52] García-Castrillo G, Olaso I (1995). Composition and structure of the invertebrate megabenthos on the shelf of the Cantabrian Sea.. ICES Mar Sci Symp (Act Symp).

[53] Olaso I, Rodriguez-Marin E (1995). Decapod crustaceans in the diets of demersal fish in the Cantabrian Sea.. ICES Mar Sci Symp (Act Symp).

[54] Cartes JE, Carrasson M (2007). Influence of trophic variables on the depth-range distributions and zonation rates of deep-sea megafauna: the case of the Western Mediterranean assemblages.. Deep-Sea Research.

[55] Polunin NVC, Morales-Nin B, Herod W, Cartes JE, Pinnegar JK (2001). Feeding relationships in Mediterranean bathyal assemblages elucidated by carbon and nitrogen stable-isotope data.. Marine Ecology Progress Series.

[56] Cartes JE, Abelló P, Lloris D, Carbonell A, Torres P (2002). Analysis of feeding guilds of fish and decapod crustaceans during the MEDITS-99 cruise along the Iberian Peninsula Mediterranean coasts.. Scientia Marina.

[57] Ratnasingham S, Hebert PDN (2007). Barcoding BOLD: The Barcode of Life Data System. Molecular Ecology Notes.

[58] Romiguier J, Ranwez V, Douzery EJP, Galtier N (2010). Contrasting GC-content dynamics across 33 mammalian genomes: Relationship with life-history traits and chromosome sizes.. Genome Research.

[59] DeSalle R, Egan MG, Siddall M (2005). The unholy trinity: taxonomy, species delimitation and DNA barcoding. Phil.. Trans. R. Soc.B.

[60] Porter ML, Pérez-Losada M, Crandall KA (2005). Model-based multi-locus estimation of decapod phylogeny and divergence times.. Molecular Phylogenetics and Evolution.

[61] Bracken HD, De Grave S, Felder DL, Martin JW, Crandall KA, Felder DL (2009). Phylogeny of the infraorder Caridea based on mitochondrial and nuclear genes (Crustacea: Decapoda).. Decapod Crustacean Phylogenetics Crustacean Issues.

[62] Martin JW, Davis GE (2001). Un updated classification of the Recent Crustacea.. Nat Hist Mus of Los Angeles.

[63] De Grave S, Cai Y, Anker A (2008). Global diversity of shrimps (Crustacea: Decapoda: Caridea) in freshwater.. Hydrobiology.

[64] Herring PJ, Dixon DR (1998). Extensive deep-sea dispersal of postlarval shrimp from a hydrothermal vent.. Deep-Sea Research.

[65] Zakšek V, Sket B, Gottstein S, Franjevic D, Trontelj P (2009). The limits of cryptic diversity in groundwater: phylogeography of the cave shrimp *Troglocaris anophthalmus* (Crustacea: Decapoda: Atyidae).. Molecular Ecology.

[66] Marin IN, Anker A, Britayev TA, Palmer AR (2005). Symbiosis between the Alpheid Shrimp, *Athanas ornithorhynchus* Banner and Banner, 1973 (Crustacea: Decapoda), and the Brittle Star, *Macrophiothrix longipeda* (Lamarck, 1816) (Echinodermata: Ophiuroidea).. Zoological Studies.

[67] Silliman BR, Layman CA, Altieri AH (2003). Symbiose between and alpheid shrimp and xanthoid crab in salt marshes of mid-atlantic states, U.S.A.. Journal of Crustacean Biology.

[68] Stevens BG, Anderson PJ (2000). An association between the anemone, Cribrinopsis fernaldi, and shrimps of the families Hippolytidae and Pandalidae.. J Northw Atl Fish Sci.

[69] Bucciarelli G, Bernardi G, Bernardi G (2002). An ultracentrifugation analysis of fish genomes.. Gene, (Special issues 3rd Anton Dohrn Workshop “Fish Genomics”).

[70] Childress JJ (1995). Are the physiological and biochemical adaptations of metabolism in deep-sea animals?. TREE.

[71] Martin AP, Palumbi SR (1993). Body Size, Metabolic-Rate, Generation Time, and the Molecular Clock.. Proceedings of the National Academy of Sciences of the United States of America.

[72] Ostrow D, Phillips N, Avalos A, Blanton D, Boggs A (2007). Mutational Bias for body Size in Rhabditid nematodes.. Genetics.

[73] Gillooly JF, Brown JH, West GB, Savage VM, Charnov EL (2001). Effects of Size and Temperature on Metabolic Rate.. Science.

[74] Gillooly JF, Allen AP, West GB, Brown JH (2005). The rate of DNA evolution: Effects of body size and temperature on the molecular clock.. Proceedings of the National Academy of Sciences of the United States of America.

[75] Gillooly JF, Allen AP (2007). Linking global patterns in biodiversity to evolutionary dynamics using metabolic theory.. Ecology.

[76] Hall S, Thatje S (2009). Global bottlenecks in the distribution of marine Crustacea: temperature constraints in the family Lithodidae.. Journal of Biogeography.

[77] Tsang LM, Ma KY, Ahyong ST, Chan T-Y, Chu KH (2008). Phylogeny of Decapoda using two nuclear protein-coding genes: Origin and evolution of the Reptantia.. Molecular Phylogenetics and Evolution.

[78] Tsang LM, Chan T-Y, Cheung MK, Chu KH (2009). Molecular evidence for the Southern Hemisphere origin and deep sea diversification of spiny lobsters (Crustacea: Decapoda: Palinuridae).. Molecular Phylogentics and Evolution.

[79] Cunningham CW, Blackstone NW, Buss LW (1992). Evolution of king crabs from hermit crab ancestors.. Nature.

[80] Zaklan SD, MacIntosh RA (2002). Review of the family Lithodidae (Crustacea: Anomura: Paguroidea): distribution, biology, and fisheries.. Crabs in cold water regions: biology, management, and economics.

[81] Martin AP, Naylor GJP, Palumbi SR (1992). Rates of mitochondrial DNA evolution in sharks are slow compared with mammals.. Nature.

[82] Martin AP, Palumbi SR (1993). Body size, metabolic rate, generation time, and the molecular clock.. Proc Natl Acad Sci USA.

[83] Seibel BA, Childress JJ (2000). Metabolism of benthic octopods (Cephalopoda) as a function of habitat depth and oxygen concentration.. Deep-Sea Research.

[84] Seibel BA, Thuesen EV, Childress JJ (1997). Decline in pelagic cephalopod metabolism with habitat depth reflects differnces in locomotory efficiency.. Biolocical Bulletin.

[85] Company JB, Sardà F (1998). Metabolic rates and energy content of deep-sea benthic decapod crustaceans in the western Mediterranean Sea.. Deep-Sea Research.

[86] Childress JJ, Cowles DL, Favuzzi JA, Mickel TJ (1990). Metabolic rates of benthic deep-sea decapod crustaceans decline with increasing depth primarily due to the decline in temperature.. Deep-Sea Research.

[87] Komai T (1999). A revision of the genus Pandalus (Crustacea: Decapoda: Caridea: Pandalidae).. Journal of Natural History.

[88] Chan T-Y, B M, B RdF (2004). The "*Plesionika rostricrescentis* (Bate, 1888)" and "*P. lophotes* Chace, 1985" species groups of *Plesionika* Bate, 1888, with descriptions of five new species (Crustacea: Decapoda: Pandalidae).. Tropical Deep-Sea Benthos.

[89] Chan T-Y, Yu H-P A new deep-sea shrimp of the genus Plesionika Bate,1888 (Crustacea: Decapoda: Pandalidae) from Taiwan; 1998 March 2000; Taiwan. pp.

[90] Chan T-Y, Yu H-P (1991). Two similar species: *Plesionika edwardsii* (Brandt, 1851) and *Plesionika crosnieri*, new species (Crustacea: Decapoda: Pandalidae).. Proceedings of the Biological Society of Washington.

[91] Gonzalez-Gorillo JI, Rodriguez A (2001). The complete larval development of the spider, *Macropodia parva* (Crustacea, Decapoda, Majidae) from laboratory culture.. Invertebrate Reproduction and Development.

[92] Noël PY (1992). Clé préliminaire d'identification des Crustacea Decapoda de France et des principales autres espèces d'Europe. In: Naturelle MNdH, ed.. Collection Patrimoines Naturels. pp.

[93] d'Udekem d'Acoz C (1999). Inventaire et distribution des crustacés décapodes de l'Atlantique nord - oriental, de la Méditerranée et des eaux continentales adjacentes au nord de 25°N.. Collection Patrimoines Naturels.

[94] Guerao G, Abelló P (1997). Larval development of the spider crab Macropodia.. J Crust Biol.

[95] Garcia-Raso JE (1987). Carideos ibéricos (Crustacea, Decapoda): sintesis.. Misc Zool.

[96] Zariquiey-Alvarez R (1968). Crustáceos Decápodos Ibéricos.. Inv Pesq.

[97] Cuesta JA, Rodríguez A (2000). Zoeal stages of the intertidal crab *Pachygrapsus marmoratus* (Fabricius,1787) (Brachyura, Grapsidae) reared in the laboratory.. Hydrobiology.

[98] Schubart CD, Cuesta JA Phylogeny of North Atlantic and Mediterranean species of Pachygrapsus (Brachyura:Grapsidae) and intraspecific variation among localities;.

[99] Arif IA, Hkhan HA (2009). Molecular markers for biodiversity analysis of wildlife animals: a brief review.. Animal Biodiversity and Conservation.

[100] Liu M-Y, Cai Y-X, Tzeng C-S (2007). Molecular Systematics of the Freshwater Prawn Genus *Macrobrachium* Bate, 1868 (Crustacea: Decapoda: Palaemonidae) Inferred from mtDNA Sequences, with Emphasis on East Asian Species.. Zoological Studies.

[101] Apte S, Smith PJ, Wallis GP (2007). Mitochondrial phylogeography of New Zealand freshwater crayfishes, *Paranephrops* spp.. Molecular Ecology.

[102] Shih H-T, Ng PKL, Schubart CD, Chang H-W (2007). Phylogeny and Phylogeography of the Genus *Geothelphusa* (Crustacea: Decapoda, Brachyura, Potamidae) in Southwestern Taiwan Basedon.. Zoological Science.

[103] Munasinghe DHN, Murphy NP, Austin CM (2003). Utility of mitochondrial DNA sequences from four gene regions for systematic studies of Australian freshwater crayfish of the genus *Cherax* (Decapoda:Parastacidae).. Journal of Crustacean Biology.

[104] Gouws G, Stewart BA, Daniels SR (2006). Phylogeographic structure of a freshwater crayfish (Decapoda: Parastacidae: *Cherax preissii*) in south-western Australia.. Marine and Freshwater Research.

[105] Williamson DI (1972). Larval Development in a Marine and a Freshwater Species of *Macrobrachium* (Decapoda, Palaemonidae).. Crustaceana.

[106] Murphy NP, Austin CM (2004). Phylogenetic relationships of the globally distributed freshwater prawn genus Macrobrachium (Crustacea: Decapoda: Palaemonidae): biogeography, taxonomy and the convergent evolution of abbreviated larval development.. Zoologica Scripta.

[107] Mashiko K, Numachi K (2000). Derivation of populations with different - aized eggs in the Palaemonid prawn *Macrobrachium nipponense*.. Journal of Crustacean Biology.

[108] Alekhnovich AV, Kulesh VF (2001). Variation in the parameters of the life cycle in prawns of the genus *Macrobrachium* Bate (Crustacea, Palaemonidae).. Russian Journal of Ecology.

[109] Wong JTY, McAndrew BJ (1990). Selection for the larval freshwater tolerance in *Macrobrachium nipponense* (de Hann).. Aquaculture.

[110] De Grave S, Ghane A (2006). The establishment of the Oriental River Prawn, *Macrobrachium nipponense* (de Haan, 1849) in Anzali Lagoon, Iran.. Aquatic Invasions.

[111] Salman DS, TJ P, MD N, Ama'al GY (2006). The invasion of *Macrobrachium nipponense* (De Haan, 1849) (Caridea: Palaemonidae) into the Southern Iraqi Marshes.. Aquatic invasions.

[112] Holthuis LB (1950). Subfamily Palaemoninae.. The Palaemonidae collected by the Siboga Snellius Expeditions with remarks on other species.The Decapoda of the Siboga Expedition. Part 10.

[113] Dimmock A, Willamson I, Mather PB (2004). The influence of environment on the morphology of *Macrobrachium australiense* (Decapoda: Palaemnidae).. Aquacult Int.

[114] Fortunato C, Sbordoni V (1998). Allozyme variation in the Mediterranean rockpool prawn (*Palaemon elegans*): environmental vs. historical determinants.. Proceedings and Abstracts of Fourth International Crustacean Congress.

[115] Kirkpatrick K, Jones MB (1985). Salinity tolerance and osmoregulation of a prawn, *Palaemon affinis* Milne Edwards (Caridea: Palaemonidae).. J Exp Mar Biol Ecol.

[116] Taylor AC, Spicer JI (1987). Metabolic responses of the prawns *Palaemon elegans* and *P. serratus* (Crustacea: Decapoda) to acute hypoxia and anoxia.. Marine Biology.

[117] Reuschel S, Cuesta JA, Schubart CD (2010). Marine biogeographic boundaries and human introduction along the European coast revealed by phylogeography of the prawn Palaemon elegans.. Molecula Phylogenetics and Evolution.

[118] Grabowski M (2006). Rapid colonization of the Polish Baltic coast by an Atlantic palaemonid shrimp *Palaemon elegans* Rathke, 1837.. Aquatic invasions.

[119] Chang C-H, Rougerie R, Chen J-H (2009). Identifying earthworms through DNA barcodes: Pitfalls and promise.. Pedobiologia.

[120] Palumbi SR (2003). Population genetics, demographic connectivity, and the design of marine reserves.. Ecological Applications.

[121] Mathews LM (2007). Evidence for restricted gene flow over small spatial scales in a marine snapping shrimp *Alpheus angulosus*.. Marine Biology.

[122] Baratti M, Goti E, Messana G (2005). High level of genetic differentiation in the marine isopod Sphaeroma terebrans (Crustacea: Isopoda: Sphaeromatidae) as inferred by mitochondrial DNA analysis.. J Exp Mar Biol Ecol.

[123] Mathews LM, Anker A (2009). Molecular phylogeny reveals extensive ancient and ongoing radiations in a snapping shrimp species complex (Crustacea, Alpheidae, *Alpheus armillatus*).. Molecular Phylogenetics and Evolution.

[124] Bierne N, Bonhomme F, David P (2003). Habitat preference and the marine - speciation paradox.. Proc R Soc Lond B.

[125] Cuesta JA, Schubart CD (1998). Morphological and molecular differentiation between three allopatric populations of the littoral crab *Pachygrapsus transversus* (Gibbes, 1850) (Brachyura: Grapsidae).. Journal of Natural History.

[126] Barber PH, Erdmann MV, Palumbi SR (2006). Comparative phylogeography of three codistributed stomatopods: origins and timing of regional lineage diversification in the coral triangle.. Evolution.

[127] Harrison JS (2004). Evolution, biogeography, and the utility of mitochondrial 16 S and COI genes in phylogenetic analysis of the crab genus Austinixa (Decapoda: Pinnotheridae).. Molecular Phylogenetics and Evolution 30.

[128] Pfeiler E, Hurtado LA, Knowles LL, Torre-Cosío J, Bourillón-Moreno L (2005). Population genetics of the swimming crab Callinectes bellicosus (Brachyura: Portunidae) from the eastern Pacific Ocean.. Marine Biology.

[129] Gomez A, Wright PJ, Lunt DH, Cancino JM, Carvalho GR (2007). Mating trials validate the use of DNA barcoding to reveal cryptic speciation of a marine bryozoan taxon.. Proceedings of the Royal Society B-Biological Sciences.

[130] Brökeland W, Raupach MJ (2008). A species complex within the isopod genus Haploniscus (Crustacea: Malacostraca: Peracarida) from the Southern Ocean deep sea: a morphological and molecular approach.. Zoological Journal of the Linnean Society.

[131] Meier R, Shiyang K, Vaidya G, Ng PKL (2006). DNA Barcoding and Taxonomy in Diptera: A Tale of High Intraspecific Variability and Low Identification Success.. Syst Biol.

[132] Radulovici AE, Sainte-Marie B, Dufresne F (2009). DNA barcoding of marine crustaceans from the Estuary and Gulf of St Lawrence: a regional-scale approach.. Molecular Ecology Resources.

[133] Hultgren KM, Stachowicz JJ (2008). Molecular phylogeny of the brachyuran crab superfamily Majoidea indicates close congruence with trees based on larval morphology.. Molecula Phylogenetics and Evolution.

[134] Song H, Buhay JE, Whiting MF, Crandall KA (2008). Many species in one: DNA barcoding overestimates the number of species when nuclear mitochondrial pseudogenes are coamplified.. PNAS.

[135] Williams ST, Knowlton N (2001). Mitochondrial pseudogenes are pervasive and often insidious in the snapping Shrimp genus Alpheus.. Mol Biol Evol.

[136] Buhay JE (2009). "COI-like" sequences are becoming problematic in molecular systematics and DNA barcoding studies.. Journal of Crustacean Biology.

[137] Bensasson D, Zhang D-X, Hartl DL, Hewitt GM (2001). Mitochondrial pseudogenes: evolution's misplaced witnesses.. Trends Ecol Evol.

[138] Schubart CD, Martin JW, Crandall, K.A, Felder, D.L (2009). Mitochondrial DNA and decapod phylogenies; the importance of pseudogenes and primer optimization.. Decapod Crustacean Phylogenetics.

[139] Nguyen TTT, Murphy NP, Austin CM (2002). Amplification of multiple copies of mitochondrial cytochrome *b* gene fragments in the Australian freshwater crayfish, *Cherax destructor* Clark (Parastacidae; Decapoda) Anim Genet.

[140] Balakirev ES, Ayala FJ (2003). PSEUDOGENES: Are They “Junk” or Functional DNA?. Annu Rev Genet.

[141] Gerstein M, Zheng D (2006). The real life of pseudogenes.. Sci Am.

[142] Taylor WR (1986). The Classification of Amino Acid Conservation.. J Theor Biol.

[143] Ward RD, Zemlak TS, Innes BH, Last PR, Hebert PDN (2005). DNA barcoding Australia's fish species.. Phil. Trans. R. Soc.B.

[144] Mooers AO, Holmes EC (2000). The evolution of base composition and phylogenetc inference.. Trends Ecol. Evol..

[145] Banerjee T, Gupta SK, Ghosh TC (2005). Role of mutational bias and natural selection on genome-wide nucleotide bias in prokaryotic organisms.. BioSystems.

[146] Somero GN (2003). Protein adaptations to temperature and pressure: complementary roles of adaptive changes in amino acid sequence and internal milieu.. Comparative Biochemistry and Physiology B-Biochemistry & Molecular Biology.

[147] Friedberg EC (1995). Out of the Shadows and into the Light - the Emergence of DNA-Repair.. Trends in Biochemical Sciences.

[148] Sicot FX, Mesnage M, Masselot M, Exposito JY, Garrone R (2000). Molecular Adaptation to an Extreme Environment: Origin of the Thermal Stability of the Pompeii Worm Collagen.. J Mol Biol.

[149] Hebert PDN, Remigio EA, Colbourne JK, Taylor DJ, Wilson CC (2002). Accelerated molecular evolution in halophilic crustaceans.. Evolution.

[150] Bjedov I, Olivier Tenaillon, Benedicte Gerard, Valeria Souza, Erick Denamur (2003). Stress-Induced Mutagenesis in Bacteria.. Science.

[151] Cinzia V, Vergara A, Giordano D, Mazzarella L, Prisco G (2006). he Root effect- a structural and evolutionary perspective.. Antarctic Science.

[152] Chandor A, Douki T, Gasparutto D, Gambarelli S, Sanakis Y (2007). Characterization of the DNA repair spore photoproduct lyase enzyme from Clostridium acetobutylicum: A radical-SAM enzyme.. Comptes Rendus Chimie.

[153] Hassanin A (2006). Phylogeny of Arthropoda inferred from mitochondrial sequences: Strategies for limiting the misleading effects of multiple changes in pattern and rates of substitution.. Molecular Phylogenetics and Evolution.

[154] Drake JW (2006). Chaos and order in spontaneous mutation.. Genetics.

[155] Ohta T (1992). The nearly neutral theory of molecular evolution.. Annu Rev Ecol Syst.

[156] Drake JW, Charlesworth B, Charlesworth D, Crow JF (1998). Rates of spontaneous mutation.. Genetics.

[157] Page RDM, Lee PLM, Becher SA, Griffiths R, Clayton DH (1998). A different tempo of mitochondrial DNA evoltution in Birds and their parasitic life.. Biology Letters.

[158] Maki H (2002). Origins of spontaneous mutations: Specificity and directionality of base-substitution, frameshift, and sequence-substitution mutageneses.. Annual Review of Genetics.

[159] Ho SYW, J Phillips M, Cooper A, Drummond AJ (2005). Time Dependency of Molecular Rate Estimates and Systematic Overestimation of Recent Divergence Times.. Mol Biol Evol.

[160] Baer CF, Miyamoto MM, Denver DR (2007). Mutation rate variation in multicellular eukaryotes: causes and consequences.. Nature Reviews Genetics.

[161] Foster PG, Jermin LS, Hickey DA (1997). Nucleotide composition bias affects amino acid content in proteins coded by animal mitochondria.. J Mol Evol.

[162] Bernardi G (1995). The human genome: organization and evolutionary history.. Annu Rev Genet.

[163] Dill KA (1990). Dominant forces in protein folding.. Biochemistry.

[164] Matz MV, Nielsen R (2005). Taxonomy - Will DNA barcodes breathe life into classification?.

[165] Zang AB, He LJ, Crozier RH, Muster C, Zhu C–D (2010). Estimating sample sizes for DNA barcoding.. Molecular Phylogenetics and Evolution.

[166] Hajibabaei M, DeWaard JR, Ivanova NV, Ratnasingham S, Dooh RT (2005). Critical factors for assembling a high volume of DNA barcodes.. Phil. Trans. R. Soc.B.

[167] Folmer O, Black M, Hoeh W, Lutz R, Vrijenhoek R (1994). DNA primers for amplification of mitochondrial cytrocrome c oxidase subunit I from diverse metazoan invertebrates.. Molecular Marine Biology and Biotechnology.

[168] Ivanova NV, Zemlak TS, Hanner RH, Hebert PDN (2007). Universal primer cocktails for fish DNA barcoding.. Molecular Ecology Notes.

[169] Werle E, Schneider C, Renner M, Volker M, Fiehn W (1994). Convenient single-step, one tube purification of PCR products for direct sequencing.. Nucl Acids Res.

[170] Thompson JD, Higgins DG, Gibson TJ (1994). CLUSTAL W: improving the sensitivity of progressive multiple sequence alignment through sequence weighting, position-specific gap penalties and weight matrix choice.. Nucleic Acids Res.

[171] Tamura K DJ, Nei M, Kumar S (2007). MEGA4: Molecular Evolutionary Genetics Analysis (MEGA) software version 4.0.. Molecular Biology and Evolution.

[172] Harris DJ (2003). Can you bank on GenBank? Trends Ecol. Evol..

[173] Matz MV, Nielsen R (2005). A likelihood ratio test for species membership based on DNA sequence data.. Phil. Trans. R. Soc.B.

[174] Meyer CP, Paulay G (2005). DNA barcodes perform best with well-characterized ta\xa.. PLOS Biology.

[175] Levesque R (2008). SPSS Programming and Data Management for SPSS 16.0: A Guide for SPSS and SAS Users..

[176] Payne RW (2009). GenStat.. Computational Statistics.

[177] Fourment M, Gibbs MJ (2006). PATRISTIC: a program for calculation patristic distances and graphically comparing the components of genetic change.. BMC Evolutionary Biology.

