# Supplementary material for: Systematic and Evolutionary Insights Derived from mtDNA COI Barcode Diversity in the Decapoda (Crustacea: Malacostraca)
Source: PLoS One. 2011 May 12;6(5):e19449. doi: 10.1371/journal.pone.0019449 (PMC3093375; doi:10.1371/journal.pone.0019449)
Supplement: Figure S1 — Taxon ID Tree of Decapoda generated by BOLD. Neighbour Joining tree (Kimura 2-parameter, uniform rates among sites, pairwise deletion) combining COI data from public BOLD projects and present study. A total number of 1906 sequences from 603 species, 225 genera and 71 families were used. (PDF) [file pone.0019449.s001.pdf]

BOLD TaxonID Tree

Project	:	SEARCH: Processids(1906)
Date	:	7-November-2010
Data Type	:	Nucleotide
Distance Model	:	Kimura 2 Parameter
Marker	:	COI-5P
Codon Positions	:	1st, 2nd, 3rd
Labels	:	SampleID, Family,
Filters	:	Length > 200

Sequence Count	:	1906
Species count	:	603
Genus count	:	225
Family count	:	71
Unidentified	:	0

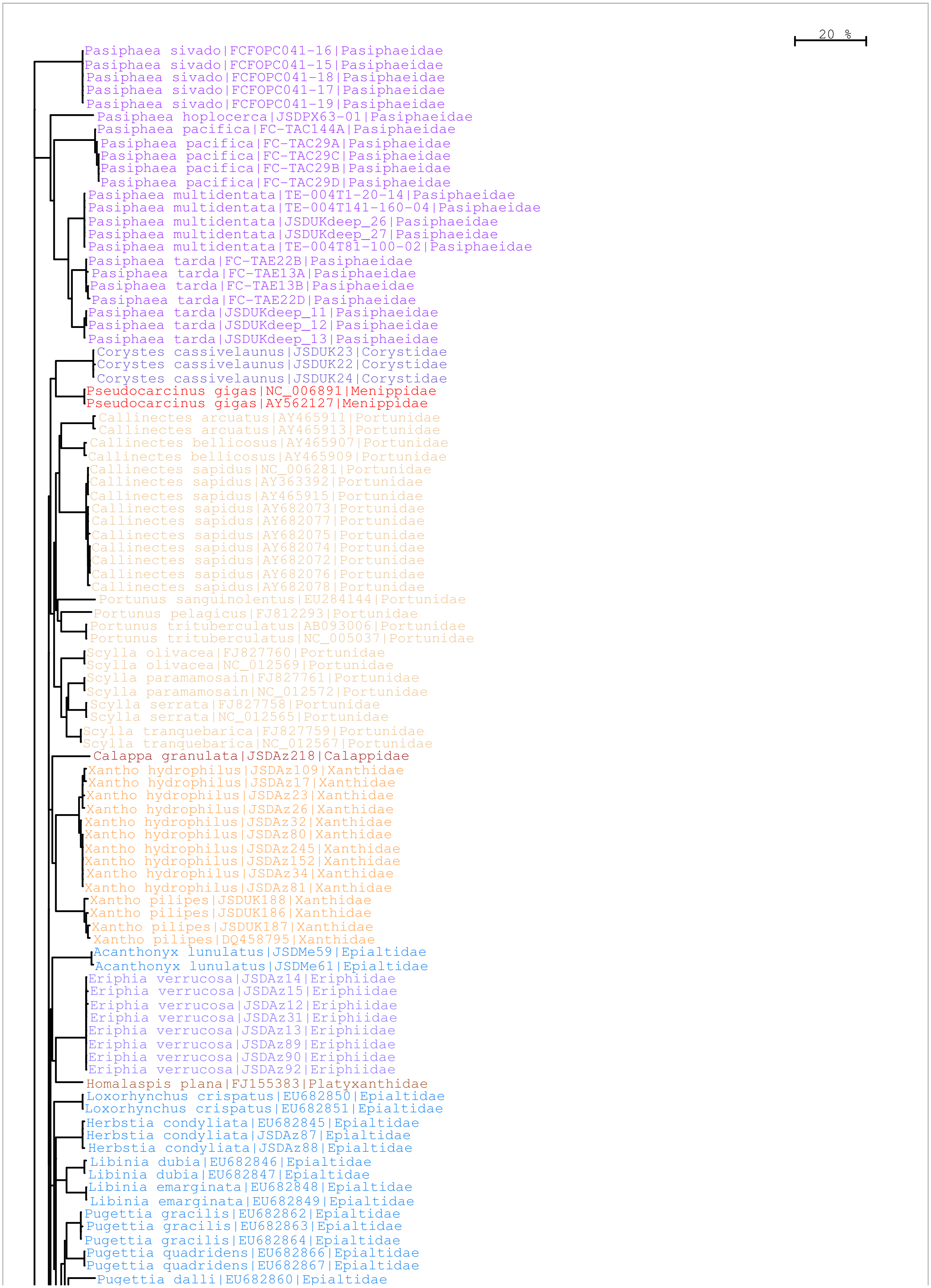
20 %
Pasiphaea sivado|FCFOPC041-16|Pasiphaeidae
Pasiphaea sivado|FCFOPC041-15|Pasiphaeidae
Pasiphaea sivado|FCFOPC041-18|Pasiphaeidae
Pasiphaea sivado|FCFOPC041-17|Pasiphaeidae
Pasiphaea sivado|FCFOPC041-19|Pasiphaeidae
Pasiphaea hoplocerca|JSDPX63-01|Pasiphaeidae
Pasiphaea pacifica|FC-TAC144A|Pasiphaeidae
Pasiphaea pacifica|FC-TAC29A|Pasiphaeidae
Pasiphaea pacifica|FC-TAC29C|Pasiphaeidae
Pasiphaea pacifica|FC-TAC29B|Pasiphaeidae
Pasiphaea pacifica|FC-TAC29D|Pasiphaeidae
Pasiphaea multidentata|TE-004T1-20-14|Pasiphaeidae
Pasiphaea multidentata|TE-004T141-160-04|Pasiphaeidae
Pasiphaea multidentata|JSDUKdeep_26|Pasiphaeidae
Pasiphaea multidentata|JSDUKdeep_27|Pasiphaeidae
Pasiphaea multidentata|TE-004T81-100-02|Pasiphaeidae
Pasiphaea tarda|FC-TAE22B|Pasiphaeidae
Pasiphaea tarda|FC-TAE13A|Pasiphaeidae
Pasiphaea tarda|FC-TAE13B|Pasiphaeidae
Pasiphaea tarda|FC-TAE22D|Pasiphaeidae
Pasiphaea tarda|JSDUKdeep_11|Pasiphaeidae
Pasiphaea tarda|JSDUKdeep_12|Pasiphaeidae
Pasiphaea tarda|JSDUKdeep_13|Pasiphaeidae
Corystes cassivelaunus|JSDUK23|Corystidae
Corystes cassivelaunus|JSDUK22|Corystidae
Corystes cassivelaunus|JSDUK24|Corystidae
Pseudocarcinus gigas|NC_006891|Menippidae
Pseudocarcinus gigas|AY562127|Menippidae
Callinectes arcuatus|AY465911|Portunidae
Callinectes arcuatus|AY465913|Portunidae
Callinectes bellicosus|AY465907|Portunidae
Callinectes bellicosus|AY465909|Portunidae
Callinectes sapidus|NC_006281|Portunidae
Callinectes sapidus|AY363392|Portunidae
Callinectes sapidus|AY465915|Portunidae
Callinectes sapidus|AY682073|Portunidae
Callinectes sapidus|AY682077|Portunidae
Callinectes sapidus|AY682075|Portunidae
Callinectes sapidus|AY682074|Portunidae
Callinectes sapidus|AY682072|Portunidae
Callinectes sapidus|AY682076|Portunidae
Callinectes sapidus|AY682078|Portunidae
Portunus sanguinolentus|EU284144|Portunidae
Portunus pelagicus|FJ812293|Portunidae
Portunus trituberculatus|AB093006|Portunidae
Portunus trituberculatus|NC_005037|Portunidae
Scylla olivacea|FJ827760|Portunidae
Scylla olivacea|NC_012569|Portunidae
Scylla paramamosain|FJ827761|Portunidae
Scylla paramamosain|NC_012572|Portunidae
Scylla serrata|FJ827758|Portunidae
Scylla serrata|NC_012565|Portunidae
Scylla tranquebarica|FJ827759|Portunidae
Scylla tranquebarica|NC_012567|Portunidae
Calappa granulata|JSDAz218|Calappidae
Xantho hydrophilus|JSDAz109|Xanthidae
Xantho hydrophilus|JSDAz17|Xanthidae
Xantho hydrophilus|JSDAz23|Xanthidae
Xantho hydrophilus|JSDAz26|Xanthidae
Xantho hydrophilus|JSDAz32|Xanthidae
Xantho hydrophilus|JSDAz80|Xanthidae
Xantho hydrophilus|JSDAz245|Xanthidae
Xantho hydrophilus|JSDAz152|Xanthidae
Xantho hydrophilus|JSDAz34|Xanthidae
Xantho hydrophilus|JSDAz81|Xanthidae
Xantho pilipes|JSDUK188|Xanthidae
Xantho pilipes|JSDUK186|Xanthidae
Xantho pilipes|JSDUK187|Xanthidae
Xantho pilipes|DQ458795|Xanthidae
Acanthonyx lunulatus|JSDMe59|Epialtidae
Acanthonyx lunulatus|JSDMe61|Epialtidae
Eriphia verrucosa|JSDAz14|Eriphiidae
Eriphia verrucosa|JSDAz15|Eriphiidae
Eriphia verrucosa|JSDAz12|Eriphiidae
Eriphia verrucosa|JSDAz31|Eriphiidae
Eriphia verrucosa|JSDAz13|Eriphiidae
Eriphia verrucosa|JSDAz89|Eriphiidae
Eriphia verrucosa|JSDAz90|Eriphiidae
Eriphia verrucosa|JSDAz92|Eriphiidae
Homalaspis plana|FJ155383|Platyxanthidae
Loxorhynchus crispatus|EU682850|Epialtidae
Loxorhynchus crispatus|EU682851|Epialtidae
Herbstia condyliata|EU682845|Epialtidae
Herbstia condyliata|JSDAz87|Epialtidae
Herbstia condyliata|JSDAz88|Epialtidae
Libinia dubia|EU682846|Epialtidae
Libinia dubia|EU682847|Epialtidae
Libinia emarginata|EU682848|Epialtidae
Libinia emarginata|EU682849|Epialtidae
Pugettia gracilis|EU682862|Epialtidae
Pugettia gracilis|EU682863|Epialtidae
Pugettia gracilis|EU682864|Epialtidae
Pugettia quadridens|EU682866|Epialtidae
Pugettia quadridens|EU682867|Epialtidae
Pugettia dalli|EU682860|Epialtidae

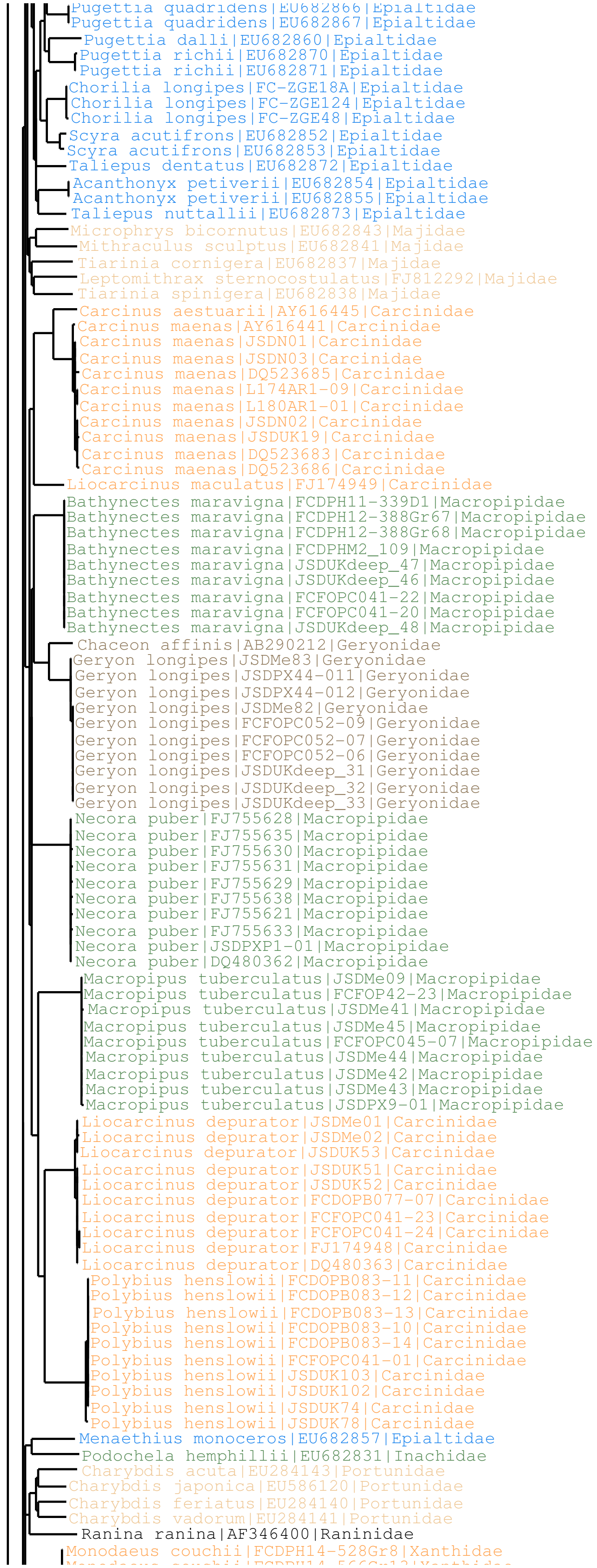
Pugettia quadridens|EU682866|Epialtidae
Pugettia quadridens|EU682867|Epialtidae
Pugettia dalli|EU682860|Epialtidae
Pugettia richii|EU682870|Epialtidae
Pugettia richii|EU682871|Epialtidae
Chorilia longipes|FC-ZGE18A|Epialtidae
Chorilia longipes|FC-ZGE124|Epialtidae
Chorilia longipes|FC-ZGE48|Epialtidae
Scyra acutifrons|EU682852|Epialtidae
Scyra acutifrons|EU682853|Epialtidae
Taliepus dentatus|EU682872|Epialtidae
Acanthonyx petiverii|EU682854|Epialtidae
Acanthonyx petiverii|EU682855|Epialtidae
Taliepus nuttallii|EU682873|Epialtidae
Microphrys bicornutus|EU682843|Majidae
Mithraculus sculptus|EU682841|Majidae
Tiarinia cornigera|EU682837|Majidae
Leptomithrax sternocostulatus|FJ812292|Majidae
Tiarinia spinigera|EU682838|Majidae
Carcinus aestuarii|AY616445|Carcinidae
Carcinus maenas|AY616441|Carcinidae
Carcinus maenas|JSDN01|Carcinidae
Carcinus maenas|JSDN03|Carcinidae
Carcinus maenas|DQ523685|Carcinidae
Carcinus maenas|L174AR1-09|Carcinidae
Carcinus maenas|L180AR1-01|Carcinidae
Carcinus maenas|JSDN02|Carcinidae
Carcinus maenas|JSDUK19|Carcinidae
Carcinus maenas|DQ523683|Carcinidae
Carcinus maenas|DQ523686|Carcinidae
Liocarcinus maculatus|FJ174949|Carcinidae
Bathynectes maravigna|FCDPH11-339D1|Macropipidae
Bathynectes maravigna|FCDPH12-388Gr67|Macropipidae
Bathynectes maravigna|FCDPH12-388Gr68|Macropipidae
Bathynectes maravigna|FCDPHM2_109|Macropipidae
Bathynectes maravigna|JSDUKdeep_47|Macropipidae
Bathynectes maravigna|JSDUKdeep_46|Macropipidae
Bathynectes maravigna|FCFOPC041-22|Macropipidae
Bathynectes maravigna|FCFOPC041-20|Macropipidae
Bathynectes maravigna|JSDUKdeep_48|Macropipidae
Chaceon affinis|AB290212|Geryonidae
Geryon longipes|JSDMe83|Geryonidae
Geryon longipes|JSDPX44-011|Geryonidae
Geryon longipes|JSDPX44-012|Geryonidae
Geryon longipes|JSDMe82|Geryonidae
Geryon longipes|FCFOPC052-09|Geryonidae
Geryon longipes|FCFOPC052-07|Geryonidae
Geryon longipes|FCFOPC052-06|Geryonidae
Geryon longipes|JSDUKdeep_31|Geryonidae
Geryon longipes|JSDUKdeep_32|Geryonidae
Geryon longipes|JSDUKdeep_33|Geryonidae
Necora puber|FJ755628|Macropipidae
Necora puber|FJ755635|Macropipidae
Necora puber|FJ755630|Macropipidae
Necora puber|FJ755631|Macropipidae
Necora puber|FJ755629|Macropipidae
Necora puber|FJ755638|Macropipidae
Necora puber|FJ755621|Macropipidae
Necora puber|FJ755633|Macropipidae
Necora puber|JSDPXP1-01|Macropipidae
Necora puber|DQ480362|Macropipidae
Macropipus tuberculatus|JSDMe09|Macropipidae
Macropipus tuberculatus|FCFOP42-23|Macropipidae
Macropipus tuberculatus|JSDMe41|Macropipidae
Macropipus tuberculatus|JSDMe45|Macropipidae
Macropipus tuberculatus|FCFOPC045-07|Macropipidae
Macropipus tuberculatus|JSDMe44|Macropipidae
Macropipus tuberculatus|JSDMe42|Macropipidae
Macropipus tuberculatus|JSDMe43|Macropipidae
Macropipus tuberculatus|JSDPX9-01|Macropipidae
Liocarcinus depurator|JSDMe01|Carcinidae
Liocarcinus depurator|JSDMe02|Carcinidae
Liocarcinus depurator|JSDUK53|Carcinidae
Liocarcinus depurator|JSDUK51|Carcinidae
Liocarcinus depurator|JSDUK52|Carcinidae
Liocarcinus depurator|FCDOPB077-07|Carcinidae
Liocarcinus depurator|FCFOPC041-23|Carcinidae
Liocarcinus depurator|FCFOPC041-24|Carcinidae
Liocarcinus depurator|FJ174948|Carcinidae
Liocarcinus depurator|DQ480363|Carcinidae
Polybius henslowii|FCDOPB083-11|Carcinidae
Polybius henslowii|FCDOPB083-12|Carcinidae
Polybius henslowii|FCDOPB083-13|Carcinidae
Polybius henslowii|FCDOPB083-10|Carcinidae
Polybius henslowii|FCDOPB083-14|Carcinidae
Polybius henslowii|FCFOPC041-01|Carcinidae
Polybius henslowii|JSDUK103|Carcinidae
Polybius henslowii|JSDUK102|Carcinidae
Polybius henslowii|JSDUK74|Carcinidae
Polybius henslowii|JSDUK78|Carcinidae
Menaethius monoceros|EU682857|Epialtidae
Podochela hemphillii|EU682831|Inachidae
Charybdis acuta|EU284143|Portunidae
Charybdis japonica|EU586120|Portunidae
Charybdis feriatus|EU284140|Portunidae
Charybdis vadorum|EU284141|Portunidae
Ranina ranina|AF346400|Raninidae
Monodaeus couchii|FCDPH14-528Gr8|Xanthidae

[illegible]
Ranina ranina|AF346400|Raninidae
Monodaeus couchii|FCDPH14-528Gr8|Xanthidae
Monodaeus couchii|FCDPH14-566Gr13|Xanthidae
Monodaeus couchii|FCDPH14-528Gr75|Xanthidae
Monodaeus couchii|JSDPX24-01|Xanthidae
Monodaeus couchii|FCDPH14-528Gr79|Xanthidae
Monodaeus couchii|FCDPH14-551D81|Xanthidae
Monodaeus couchii|FCDPH14-559B83|Xanthidae
Monodaeus couchii|FCDPHM21Bx_110|Xanthidae
Monodaeus couchii|FCDPHMSM242_98|Xanthidae
Monodaeus couchii|JSDPX24-02|Xanthidae
Chionoecetes angulatus|FC-ZAE15A|Oregoniidae
Chionoecetes angulatus|FC-ZAE15B|Oregoniidae
Chionoecetes opilio|EU266370|Oregoniidae
Chionoecetes opilio|GSL31-01|Oregoniidae
Chionoecetes opilio|BSM08-L03|Oregoniidae
Chionoecetes opilio|EU682832|Oregoniidae
Chionoecetes opilio|EU682833|Oregoniidae
Chionoecetes opilio|BSM08-L01|Oregoniidae
Chionoecetes opilio|BSM08-L02|Oregoniidae
Chionoecetes opilio|CO01CN0706|Oregoniidae
Chionoecetes opilio|CO02CN0706|Oregoniidae
Chionoecetes bairdi|AB211158|Oregoniidae
Chionoecetes bairdi|AB211157|Oregoniidae
Chionoecetes bairdi|FC-ZAF34|Oregoniidae
Chionoecetes bairdi|AB211155|Oregoniidae
Chionoecetes bairdi|FC-ZAF166|Oregoniidae
Chionoecetes bairdi|FC-ZAF29|Oregoniidae
Chionoecetes bairdi|AB211156|Oregoniidae
Chionoecetes bairdi|AB211159|Oregoniidae
Chionoecetes tanneri|FC-ZAG11A|Oregoniidae
Chionoecetes tanneri|FC-ZAG11B|Oregoniidae
Hyas araneus|JSDSv08|Oregoniidae
Hyas araneus|GSL31-18|Oregoniidae
Hyas araneus|GSL31-19|Oregoniidae
Hyas araneus|HAR02CN0806|Oregoniidae
Hyas araneus|TE-004T196-01|Oregoniidae
Hyas araneus|HAR01CN0806|Oregoniidae
Hyas araneus|JSDSv07|Oregoniidae
Hyas araneus|EU682834|Oregoniidae
Hyas araneus|BSM07T1-24|Oregoniidae
Hyas araneus|BSM07T1-29|Oregoniidae
Hyas coarctatus|JSDUK176|Oregoniidae
Hyas coarctatus|HC01CN0806|Oregoniidae
Hyas coarctatus|HC02CN0806|Oregoniidae
Hyas coarctatus|BSM07T9-01|Oregoniidae
Hyas coarctatus|GSL31-23|Oregoniidae
Hyas coarctatus|GSL31-21|Oregoniidae
Hyas coarctatus|BSM07T1-28|Oregoniidae
Hyas coarctatus|BSM07T9-02|Oregoniidae
Hyas lyratus|FC-ZBA23|Oregoniidae
Macroregonia macrochira|AB478047|Oregoniidae
Macroregonia macrochira|AB478050|Oregoniidae
Macroregonia macrochira|AB478065|Oregoniidae
Macroregonia macrochira|AB478070|Oregoniidae
Macroregonia macrochira|AB478071|Oregoniidae
Macroregonia macrochira|AB478051|Oregoniidae
Macroregonia macrochira|AB478048|Oregoniidae
Macroregonia macrochira|AB478046|Oregoniidae
Macroregonia macrochira|AB478061|Oregoniidae
Macroregonia macrochira|AB478074|Oregoniidae
Oregonia gracilis|EU682836|Oregoniidae
Atelecyclus rotundatus|JSDUK01|Atelecyclidae
Atelecyclus rotundatus|JSDPX22-01|Atelecyclidae
Atelecyclus rotundatus|FCDOPB077-04|Atelecyclidae
Atelecyclus rotundatus|JSDUK02|Atelecyclidae
Cancer irroratus|BSM07T1-26|Cancridae
Cancer irroratus|GSL31-09|Cancridae
Cancer irroratus|GSL31-20|Cancridae
Cancer irroratus|L195AR2-01|Cancridae
Cancer irroratus|L210AR4-01|Cancridae
Cancer irroratus|CI01MD0204|Cancridae
Cancer irroratus|CI02MD0204|Cancridae
Cancer irroratus|CI04MD0306|Cancridae
Cancer magister|FC-XKG73D|Cancridae
Cancer magister|FC-XKG73E|Cancridae
Cancer bellianus|JSDPX44-10|Cancridae
Cancer pagurus|JSDUK10|Cancridae
Cancer pagurus|JSDUK11|Cancridae
Cancer porteri|FJ155371|Cancridae
Cancer productus|FC-XLA|Cancridae
Cancer productus|FC-XLA51|Cancridae
Cancer plebejus|FJ155376|Cancridae
Cancer plebejus|FJ155375|Cancridae
Cancer plebejus|FJ155377|Cancridae
Metacarcinus edwardsii|FJ155372|Cancridae
Metacarcinus edwardsii|FJ155373|Cancridae
Metacarcinus edwardsii|FJ155374|Cancridae
Cancer oregonensis|FC-XKI 48|Cancridae
Cancer oregonensis|FC-XKI|Cancridae
Romaleon polyodon|FJ155378|Cancridae
Romaleon polyodon|FJ155379|Cancridae
Romaleon polyodon|FJ155380|Cancridae
Romaleon polyodon|FJ155381|Cancridae
Romaleon polyodon|FJ155382|Cancridae
Dyspanopeus sayi|L154AR1-06|Panopeidae
Dyspanopeus sayi|L183AR1-01|Panopeidae
Dyspanopeus sayi|L228AR1-02|Panopeidae

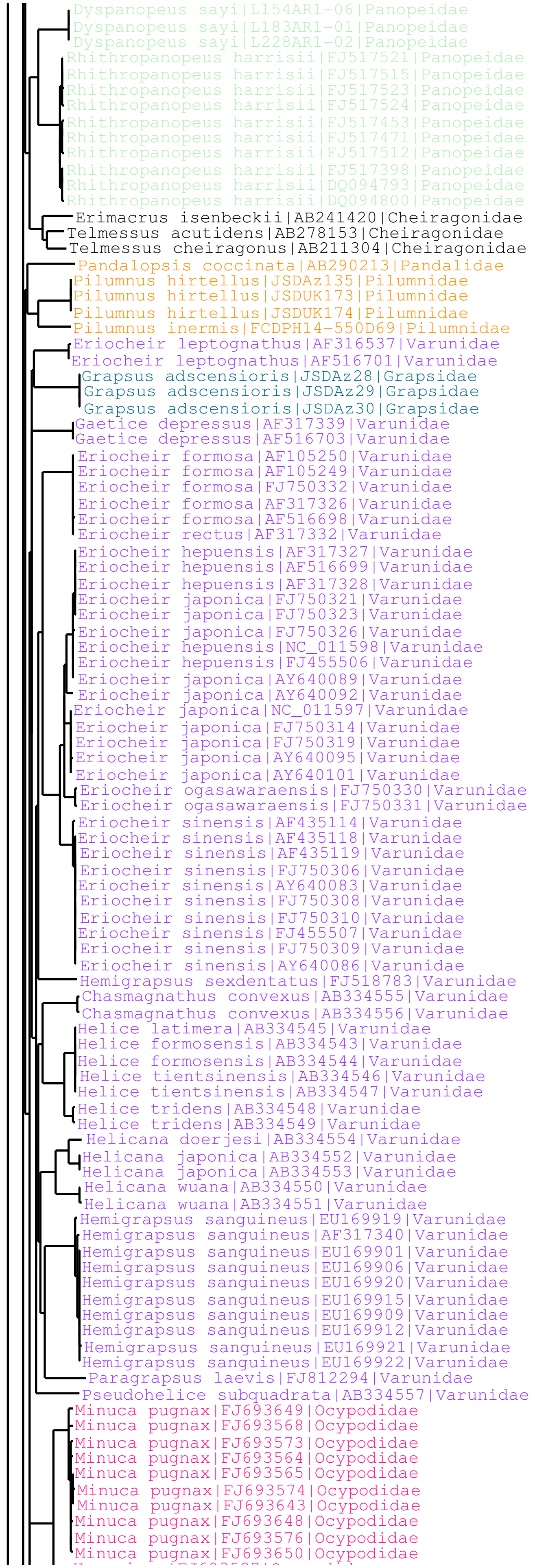
Dyspanopeus sayi|L154AR1-06|Panopeidae
Dyspanopeus sayi|L183AR1-01|Panopeidae
Dyspanopeus sayi|L228AR1-02|Panopeidae
Rhithropanopeus harrisii|FJ517521|Panopeidae
Rhithropanopeus harrisii|FJ517515|Panopeidae
Rhithropanopeus harrisii|FJ517523|Panopeidae
Rhithropanopeus harrisii|FJ517524|Panopeidae
Rhithropanopeus harrisii|FJ517453|Panopeidae
Rhithropanopeus harrisii|FJ517471|Panopeidae
Rhithropanopeus harrisii|FJ517512|Panopeidae
Rhithropanopeus harrisii|FJ517398|Panopeidae
Rhithropanopeus harrisii|DQ094793|Panopeidae
Rhithropanopeus harrisii|DQ094800|Panopeidae
Erimacrus isenbeckii|AB241420|Cheiragonidae
Telmessus acutidens|AB278153|Cheiragonidae
Telmessus cheiragonus|AB211304|Cheiragonidae
Pandalopsis coccinata|AB290213|Pandalidae
Pilumnus hirtellus|JSDAz135|Pilumnidae
Pilumnus hirtellus|JSDUK173|Pilumnidae
Pilumnus hirtellus|JSDUK174|Pilumnidae
Pilumnus inermis|FCDPH14-550D69|Pilumnidae
Eriocheir leptognathus|AF316537|Varunidae
Eriocheir leptognathus|AF516701|Varunidae
Grapsus adscensioris|JSDAz28|Grapsidae
Grapsus adscensioris|JSDAz29|Grapsidae
Grapsus adscensioris|JSDAz30|Grapsidae
Gaetice depressus|AF317339|Varunidae
Gaetice depressus|AF516703|Varunidae
Eriocheir formosa|AF105250|Varunidae
Eriocheir formosa|AF105249|Varunidae
Eriocheir formosa|FJ750332|Varunidae
Eriocheir formosa|AF317326|Varunidae
Eriocheir formosa|AF516698|Varunidae
Eriocheir rectus|AF317332|Varunidae
Eriocheir hepuensis|AF317327|Varunidae
Eriocheir hepuensis|AF516699|Varunidae
Eriocheir hepuensis|AF317328|Varunidae
Eriocheir japonica|FJ750321|Varunidae
Eriocheir japonica|FJ750323|Varunidae
Eriocheir japonica|FJ750326|Varunidae
Eriocheir hepuensis|NC_011598|Varunidae
Eriocheir hepuensis|FJ455506|Varunidae
Eriocheir japonica|AY640089|Varunidae
Eriocheir japonica|AY640092|Varunidae
Eriocheir japonica|NC_011597|Varunidae
Eriocheir japonica|FJ750314|Varunidae
Eriocheir japonica|FJ750319|Varunidae
Eriocheir japonica|AY640095|Varunidae
Eriocheir japonica|AY640101|Varunidae
Eriocheir ogasawaraensis|FJ750330|Varunidae
Eriocheir ogasawaraensis|FJ750331|Varunidae
Eriocheir sinensis|AF435114|Varunidae
Eriocheir sinensis|AF435118|Varunidae
Eriocheir sinensis|AF435119|Varunidae
Eriocheir sinensis|FJ750306|Varunidae
Eriocheir sinensis|AY640083|Varunidae
Eriocheir sinensis|FJ750308|Varunidae
Eriocheir sinensis|FJ750310|Varunidae
Eriocheir sinensis|FJ455507|Varunidae
Eriocheir sinensis|FJ750309|Varunidae
Eriocheir sinensis|AY640086|Varunidae
Hemigrapsus sexdentatus|FJ518783|Varunidae
Chasmagnathus convexus|AB334555|Varunidae
Chasmagnathus convexus|AB334556|Varunidae
Helice latimera|AB334545|Varunidae
Helice formosensis|AB334543|Varunidae
Helice formosensis|AB334544|Varunidae
Helice tientsinensis|AB334546|Varunidae
Helice tientsinensis|AB334547|Varunidae
Helice tridens|AB334548|Varunidae
Helice tridens|AB334549|Varunidae
Helicana doerjesi|AB334554|Varunidae
Helicana japonica|AB334552|Varunidae
Helicana japonica|AB334553|Varunidae
Helicana wuana|AB334550|Varunidae
Helicana wuana|AB334551|Varunidae
Hemigrapsus sanguineus|EU169919|Varunidae
Hemigrapsus sanguineus|AF317340|Varunidae
Hemigrapsus sanguineus|EU169901|Varunidae
Hemigrapsus sanguineus|EU169906|Varunidae
Hemigrapsus sanguineus|EU169920|Varunidae
Hemigrapsus sanguineus|EU169915|Varunidae
Hemigrapsus sanguineus|EU169909|Varunidae
Hemigrapsus sanguineus|EU169912|Varunidae
Hemigrapsus sanguineus|EU169921|Varunidae
Hemigrapsus sanguineus|EU169922|Varunidae
Paragrapsus laevis|FJ812294|Varunidae
Pseudohelice subquadrata|AB334557|Varunidae
Minuca pugnax|FJ693649|Ocypodidae
Minuca pugnax|FJ693568|Ocypodidae
Minuca pugnax|FJ693573|Ocypodidae
Minuca pugnax|FJ693564|Ocypodidae
Minuca pugnax|FJ693565|Ocypodidae
Minuca pugnax|FJ693574|Ocypodidae
Minuca pugnax|FJ693643|Ocypodidae
Minuca pugnax|FJ693648|Ocypodidae
Minuca pugnax|FJ693576|Ocypodidae
Minuca pugnax|FJ693650|Ocypodidae

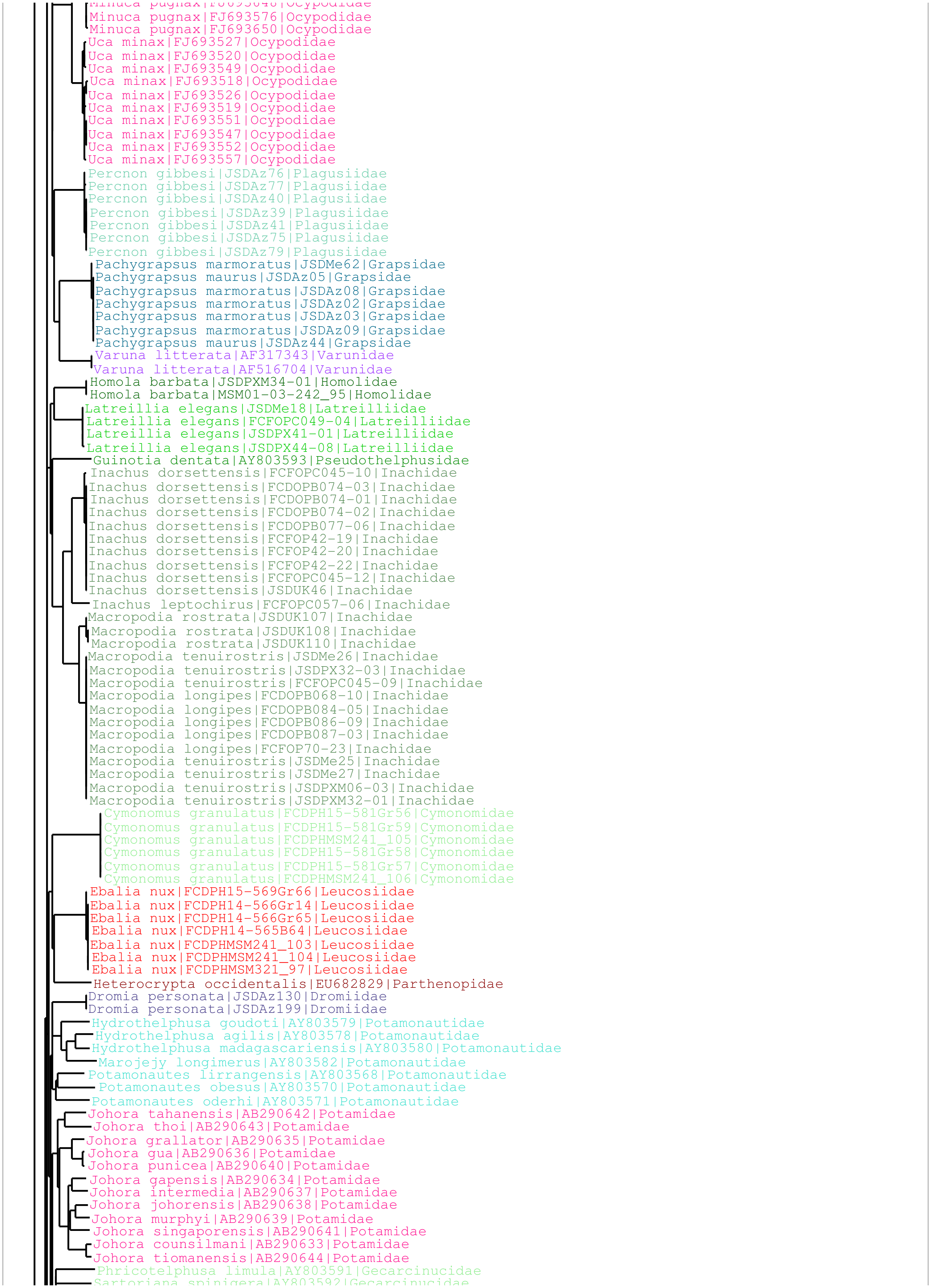
Minuca pugnax|FJ693576|Ocypodidae
Minuca pugnax|FJ693650|Ocypodidae
Uca minax|FJ693527|Ocypodidae
Uca minax|FJ693520|Ocypodidae
Uca minax|FJ693549|Ocypodidae
Uca minax|FJ693518|Ocypodidae
Uca minax|FJ693526|Ocypodidae
Uca minax|FJ693519|Ocypodidae
Uca minax|FJ693551|Ocypodidae
Uca minax|FJ693547|Ocypodidae
Uca minax|FJ693552|Ocypodidae
Uca minax|FJ693557|Ocypodidae
Percnon gibbesi|JSDAz76|Plagusiidae
Percnon gibbesi|JSDAz77|Plagusiidae
Percnon gibbesi|JSDAz40|Plagusiidae
Percnon gibbesi|JSDAz39|Plagusiidae
Percnon gibbesi|JSDAz41|Plagusiidae
Percnon gibbesi|JSDAz75|Plagusiidae
Percnon gibbesi|JSDAz79|Plagusiidae
Pachygrapsus marmoratus|JSDMe62|Grapsidae
Pachygrapsus maurus|JSDAz05|Grapsidae
Pachygrapsus marmoratus|JSDAz08|Grapsidae
Pachygrapsus marmoratus|JSDAz02|Grapsidae
Pachygrapsus marmoratus|JSDAz03|Grapsidae
Pachygrapsus marmoratus|JSDAz09|Grapsidae
Pachygrapsus maurus|JSDAz44|Grapsidae
Varuna litterata|AF317343|Varunidae
Varuna litterata|AF516704|Varunidae
Homola barbata|JSDPXM34-01|Homolidae
Homola barbata|MSM01-03-242_95|Homolidae
Latreillia elegans|JSDMe18|Latreilliidae
Latreillia elegans|FCFOPC049-04|Latreilliidae
Latreillia elegans|JSDPX41-01|Latreilliidae
Latreillia elegans|JSDPX44-08|Latreilliidae
Guinotia dentata|AY803593|Pseudothelphusidae
Inachus dorsettensis|FCFOPC045-10|Inachidae
Inachus dorsettensis|FCDOPB074-03|Inachidae
Inachus dorsettensis|FCDOPB074-01|Inachidae
Inachus dorsettensis|FCDOPB074-02|Inachidae
Inachus dorsettensis|FCDOPB077-06|Inachidae
Inachus dorsettensis|FCFOP42-19|Inachidae
Inachus dorsettensis|FCFOP42-20|Inachidae
Inachus dorsettensis|FCFOP42-22|Inachidae
Inachus dorsettensis|FCFOPC045-12|Inachidae
Inachus dorsettensis|JSDUK46|Inachidae
Inachus leptochirus|FCFOPC057-06|Inachidae
Macropodia rostrata|JSDUK107|Inachidae
Macropodia rostrata|JSDUK108|Inachidae
Macropodia rostrata|JSDUK110|Inachidae
Macropodia tenuirostris|JSDMe26|Inachidae
Macropodia tenuirostris|JSDPX32-03|Inachidae
Macropodia tenuirostris|FCFOPC045-09|Inachidae
Macropodia longipes|FCDOPB068-10|Inachidae
Macropodia longipes|FCDOPB084-05|Inachidae
Macropodia longipes|FCDOPB086-09|Inachidae
Macropodia longipes|FCDOPB087-03|Inachidae
Macropodia longipes|FCFOP70-23|Inachidae
Macropodia tenuirostris|JSDMe25|Inachidae
Macropodia tenuirostris|JSDMe27|Inachidae
Macropodia tenuirostris|JSDPXM06-03|Inachidae
Macropodia tenuirostris|JSDPXM32-01|Inachidae
Cymonomus granulatus|FCDPH15-581Gr56|Cymonomidae
Cymonomus granulatus|FCDPH15-581Gr59|Cymonomidae
Cymonomus granulatus|FCDPHMSM241_105|Cymonomidae
Cymonomus granulatus|FCDPH15-581Gr58|Cymonomidae
Cymonomus granulatus|FCDPH15-581Gr57|Cymonomidae
Cymonomus granulatus|FCDPHMSM241_106|Cymonomidae
Ebalia nux|FCDPH15-569Gr66|Leucosiidae
Ebalia nux|FCDPH14-566Gr14|Leucosiidae
Ebalia nux|FCDPH14-566Gr65|Leucosiidae
Ebalia nux|FCDPH14-565B64|Leucosiidae
Ebalia nux|FCDPHMSM241_103|Leucosiidae
Ebalia nux|FCDPHMSM241_104|Leucosiidae
Ebalia nux|FCDPHMSM321_97|Leucosiidae
Heterocrypta occidentalis|EU682829|Parthenopidae
Dromia personata|JSDAz130|Dromiidae
Dromia personata|JSDAz199|Dromiidae
Hydrothelphusa goudoti|AY803579|Potamonautidae
Hydrothelphusa agilis|AY803578|Potamonautidae
Hydrothelphusa madagascariensis|AY803580|Potamonautidae
Marojejy longimerus|AY803582|Potamonautidae
Potamonautes lirrangensis|AY803568|Potamonautidae
Potamonautes obesus|AY803570|Potamonautidae
Potamonautes oderhi|AY803571|Potamonautidae
Johora tahanensis|AB290642|Potamidae
Johora thoi|AB290643|Potamidae
Johora grallator|AB290635|Potamidae
Johora gua|AB290636|Potamidae
Johora punicea|AB290640|Potamidae
Johora gapensis|AB290634|Potamidae
Johora intermedia|AB290637|Potamidae
Johora johorensis|AB290638|Potamidae
Johora murphyi|AB290639|Potamidae
Johora singaporensis|AB290641|Potamidae
Johora counsilmani|AB290633|Potamidae
Johora tiomanensis|AB290644|Potamidae
Phricotelphusa limula|AY803591|Gecarcinucidae
Sartoriana spinigera|AY803592|Gecarcinucidae

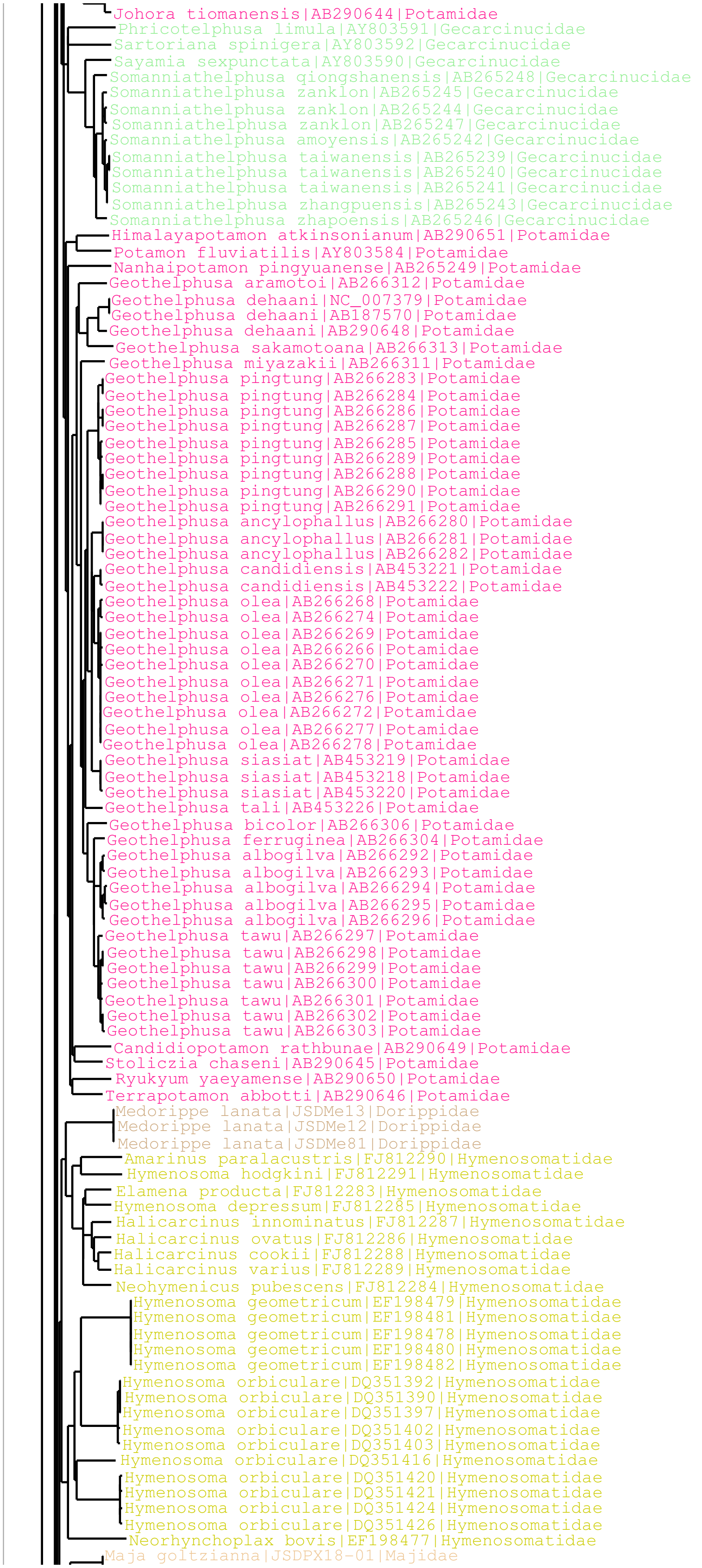
Johora tiomanensis|AB290644|Potamidae
Phricotelphusa limula|AY803591|Gecarcinucidae
Sartoriana spinigera|AY803592|Gecarcinucidae
Sayamia sexpunctata|AY803590|Gecarcinucidae
Somanniathelphusa qiongshanensis|AB265248|Gecarcinucidae
Somanniathelphusa zanklon|AB265245|Gecarcinucidae
Somanniathelphusa zanklon|AB265244|Gecarcinucidae
Somanniathelphusa zanklon|AB265247|Gecarcinucidae
Somanniathelphusa amoyensis|AB265242|Gecarcinucidae
Somanniathelphusa taiwanensis|AB265239|Gecarcinucidae
Somanniathelphusa taiwanensis|AB265240|Gecarcinucidae
Somanniathelphusa taiwanensis|AB265241|Gecarcinucidae
Somanniathelphusa zhangpuensis|AB265243|Gecarcinucidae
Somanniathelphusa zhapoensis|AB265246|Gecarcinucidae
Himalayapotamon atkinsonianum|AB290651|Potamidae
Potamon fluviatilis|AY803584|Potamidae
Nanhaipotamon pingyuanense|AB265249|Potamidae
Geothelphusa aramotoi|AB266312|Potamidae
Geothelphusa dehaani|NC_007379|Potamidae
Geothelphusa dehaani|AB187570|Potamidae
Geothelphusa dehaani|AB290648|Potamidae
Geothelphusa sakamotoana|AB266313|Potamidae
Geothelphusa miyazakii|AB266311|Potamidae
Geothelphusa pingtung|AB266283|Potamidae
Geothelphusa pingtung|AB266284|Potamidae
Geothelphusa pingtung|AB266286|Potamidae
Geothelphusa pingtung|AB266287|Potamidae
Geothelphusa pingtung|AB266285|Potamidae
Geothelphusa pingtung|AB266289|Potamidae
Geothelphusa pingtung|AB266288|Potamidae
Geothelphusa pingtung|AB266290|Potamidae
Geothelphusa pingtung|AB266291|Potamidae
Geothelphusa ancylophallus|AB266280|Potamidae
Geothelphusa ancylophallus|AB266281|Potamidae
Geothelphusa ancylophallus|AB266282|Potamidae
Geothelphusa candidiensis|AB453221|Potamidae
Geothelphusa candidiensis|AB453222|Potamidae
Geothelphusa olea|AB266268|Potamidae
Geothelphusa olea|AB266274|Potamidae
Geothelphusa olea|AB266269|Potamidae
Geothelphusa olea|AB266266|Potamidae
Geothelphusa olea|AB266270|Potamidae
Geothelphusa olea|AB266271|Potamidae
Geothelphusa olea|AB266276|Potamidae
Geothelphusa olea|AB266272|Potamidae
Geothelphusa olea|AB266277|Potamidae
Geothelphusa olea|AB266278|Potamidae
Geothelphusa siasiat|AB453219|Potamidae
Geothelphusa siasiat|AB453218|Potamidae
Geothelphusa siasiat|AB453220|Potamidae
Geothelphusa tali|AB453226|Potamidae
Geothelphusa bicolor|AB266306|Potamidae
Geothelphusa ferruginea|AB266304|Potamidae
Geothelphusa albogilva|AB266292|Potamidae
Geothelphusa albogilva|AB266293|Potamidae
Geothelphusa albogilva|AB266294|Potamidae
Geothelphusa albogilva|AB266295|Potamidae
Geothelphusa albogilva|AB266296|Potamidae
Geothelphusa tawu|AB266297|Potamidae
Geothelphusa tawu|AB266298|Potamidae
Geothelphusa tawu|AB266299|Potamidae
Geothelphusa tawu|AB266300|Potamidae
Geothelphusa tawu|AB266301|Potamidae
Geothelphusa tawu|AB266302|Potamidae
Geothelphusa tawu|AB266303|Potamidae
Candidiopotamon rathbunae|AB290649|Potamidae
Stoliczia chaseni|AB290645|Potamidae
Ryukyum yaeyamense|AB290650|Potamidae
Terrapotamon abbotti|AB290646|Potamidae
Medorippe lanata|JSDMe13|Dorippidae
Medorippe lanata|JSDMe12|Dorippidae
Medorippe lanata|JSDMe81|Dorippidae
Amarinus paralacustris|FJ812290|Hymenosomatidae
Hymenosoma hodgkini|FJ812291|Hymenosomatidae
Elamena producta|FJ812283|Hymenosomatidae
Hymenosoma depressum|FJ812285|Hymenosomatidae
Halicarcinus innominatus|FJ812287|Hymenosomatidae
Halicarcinus ovatus|FJ812286|Hymenosomatidae
Halicarcinus cookii|FJ812288|Hymenosomatidae
Halicarcinus varius|FJ812289|Hymenosomatidae
Neohymenicus pubescens|FJ812284|Hymenosomatidae
Hymenosoma geometricum|EF198479|Hymenosomatidae
Hymenosoma geometricum|EF198481|Hymenosomatidae
Hymenosoma geometricum|EF198478|Hymenosomatidae
Hymenosoma geometricum|EF198480|Hymenosomatidae
Hymenosoma geometricum|EF198482|Hymenosomatidae
Hymenosoma orbiculare|DQ351392|Hymenosomatidae
Hymenosoma orbiculare|DQ351390|Hymenosomatidae
Hymenosoma orbiculare|DQ351397|Hymenosomatidae
Hymenosoma orbiculare|DQ351402|Hymenosomatidae
Hymenosoma orbiculare|DQ351403|Hymenosomatidae
Hymenosoma orbiculare|DQ351416|Hymenosomatidae
Hymenosoma orbiculare|DQ351420|Hymenosomatidae
Hymenosoma orbiculare|DQ351421|Hymenosomatidae
Hymenosoma orbiculare|DQ351424|Hymenosomatidae
Hymenosoma orbiculare|DQ351426|Hymenosomatidae
Neorhynchoplax bovis|EF198477|Hymenosomatidae
Maja goltzianna|JSDPX18-01|Majidae

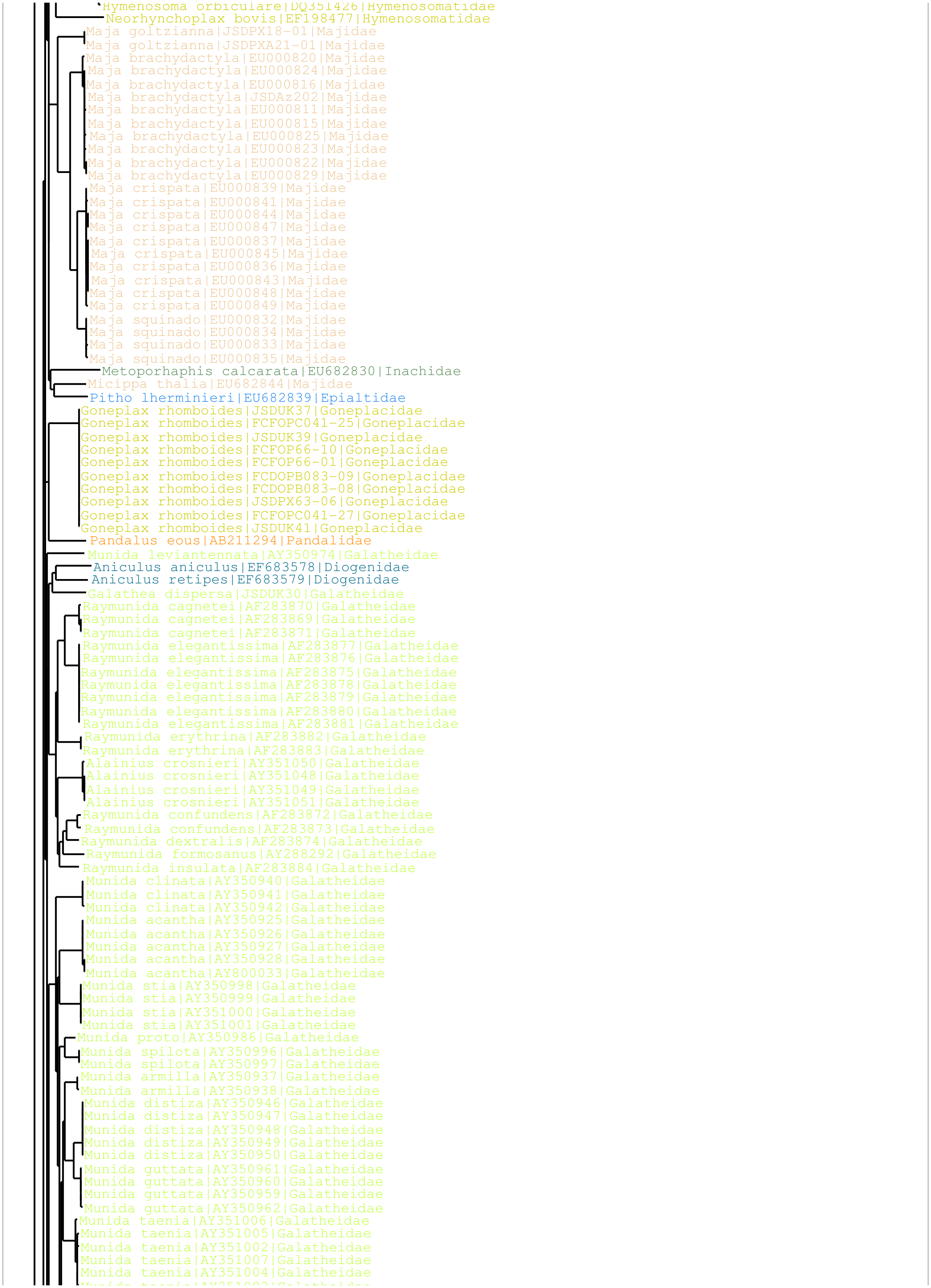
Hymenosoma orbiculare|DQ351426|Hymenosomatidae
Neorhynchoplax bovis|EF198477|Hymenosomatidae
Maja goltzianna|JSDPX18-01|Majidae
Maja goltzianna|JSDPXA21-01|Majidae
Maja brachydactyla|EU000820|Majidae
Maja brachydactyla|EU000824|Majidae
Maja brachydactyla|EU000816|Majidae
Maja brachydactyla|JSDAz202|Majidae
Maja brachydactyla|EU000811|Majidae
Maja brachydactyla|EU000815|Majidae
Maja brachydactyla|EU000825|Majidae
Maja brachydactyla|EU000823|Majidae
Maja brachydactyla|EU000822|Majidae
Maja brachydactyla|EU000829|Majidae
Maja crispata|EU000839|Majidae
Maja crispata|EU000841|Majidae
Maja crispata|EU000844|Majidae
Maja crispata|EU000847|Majidae
Maja crispata|EU000837|Majidae
Maja crispata|EU000845|Majidae
Maja crispata|EU000836|Majidae
Maja crispata|EU000843|Majidae
Maja crispata|EU000848|Majidae
Maja crispata|EU000849|Majidae
Maja squinado|EU000832|Majidae
Maja squinado|EU000834|Majidae
Maja squinado|EU000833|Majidae
Maja squinado|EU000835|Majidae
Metoporhaphis calcarata|EU682830|Inachidae
Micippa thalia|EU682844|Majidae
Pitho lherminieri|EU682839|Epialtidae
Goneplax rhomboides|JSDUK37|Goneplacidae
Goneplax rhomboides|FCFOPC041-25|Goneplacidae
Goneplax rhomboides|JSDUK39|Goneplacidae
Goneplax rhomboides|FCFOP66-10|Goneplacidae
Goneplax rhomboides|FCFOP66-01|Goneplacidae
Goneplax rhomboides|FCDOPB083-09|Goneplacidae
Goneplax rhomboides|FCDOPB083-08|Goneplacidae
Goneplax rhomboides|JSDPX63-06|Goneplacidae
Goneplax rhomboides|FCFOPC041-27|Goneplacidae
Goneplax rhomboides|JSDUK41|Goneplacidae
Pandalus eous|AB211294|Pandalidae
Munida leviantennata|AY350974|Galatheidae
Aniculus aniculus|EF683578|Diogenidae
Aniculus retipes|EF683579|Diogenidae
Galathea dispersa|JSDUK30|Galatheidae
Raymunida cagnetei|AF283870|Galatheidae
Raymunida cagnetei|AF283869|Galatheidae
Raymunida cagnetei|AF283871|Galatheidae
Raymunida elegantissima|AF283877|Galatheidae
Raymunida elegantissima|AF283876|Galatheidae
Raymunida elegantissima|AF283875|Galatheidae
Raymunida elegantissima|AF283878|Galatheidae
Raymunida elegantissima|AF283879|Galatheidae
Raymunida elegantissima|AF283880|Galatheidae
Raymunida elegantissima|AF283881|Galatheidae
Raymunida erythrina|AF283882|Galatheidae
Raymunida erythrina|AF283883|Galatheidae
Alainius crosnieri|AY351050|Galatheidae
Alainius crosnieri|AY351048|Galatheidae
Alainius crosnieri|AY351049|Galatheidae
Alainius crosnieri|AY351051|Galatheidae
Raymunida confundens|AF283872|Galatheidae
Raymunida confundens|AF283873|Galatheidae
Raymunida dextralis|AF283874|Galatheidae
Raymunida formosanus|AY288292|Galatheidae
Raymunida insulata|AF283884|Galatheidae
Munida clinata|AY350940|Galatheidae
Munida clinata|AY350941|Galatheidae
Munida clinata|AY350942|Galatheidae
Munida acantha|AY350925|Galatheidae
Munida acantha|AY350926|Galatheidae
Munida acantha|AY350927|Galatheidae
Munida acantha|AY350928|Galatheidae
Munida acantha|AY800033|Galatheidae
Munida stia|AY350998|Galatheidae
Munida stia|AY350999|Galatheidae
Munida stia|AY351000|Galatheidae
Munida stia|AY351001|Galatheidae
Munida proto|AY350986|Galatheidae
Munida spilota|AY350996|Galatheidae
Munida spilota|AY350997|Galatheidae
Munida armilla|AY350937|Galatheidae
Munida armilla|AY350938|Galatheidae
Munida distiza|AY350946|Galatheidae
Munida distiza|AY350947|Galatheidae
Munida distiza|AY350948|Galatheidae
Munida distiza|AY350949|Galatheidae
Munida distiza|AY350950|Galatheidae
Munida guttata|AY350961|Galatheidae
Munida guttata|AY350960|Galatheidae
Munida guttata|AY350959|Galatheidae
Munida guttata|AY350962|Galatheidae
Munida taenia|AY351006|Galatheidae
Munida taenia|AY351005|Galatheidae
Munida taenia|AY351002|Galatheidae
Munida taenia|AY351007|Galatheidae
Munida taenia|AY351004|Galatheidae

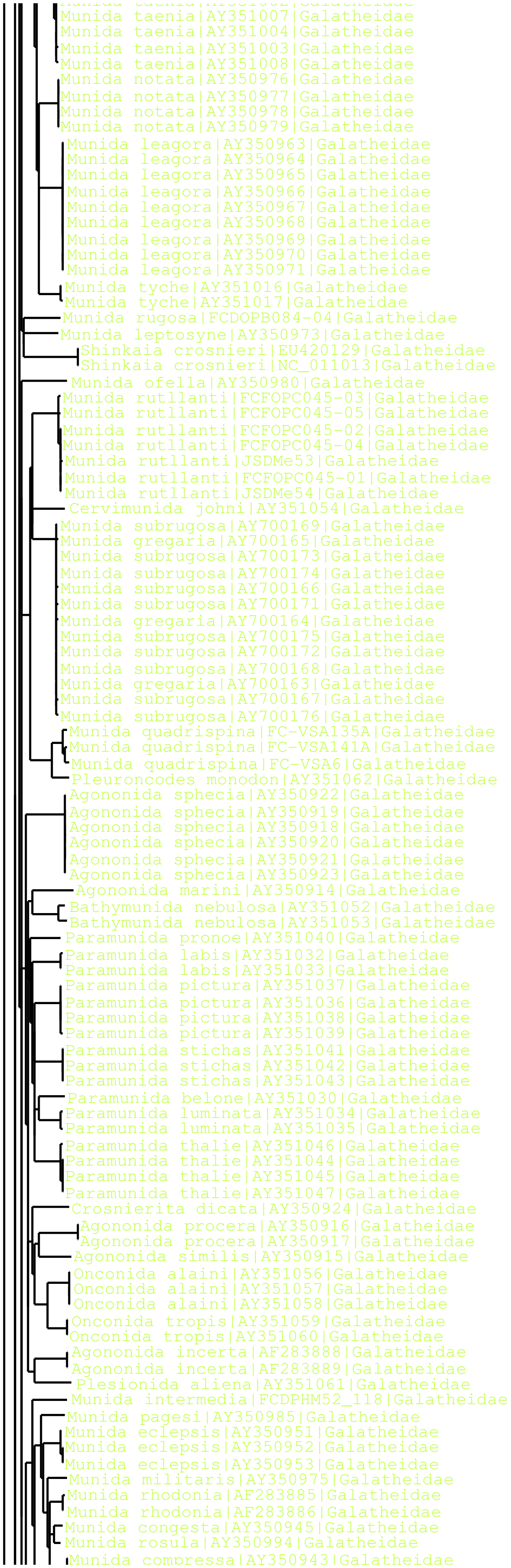
Munida taenia|AY351007|Galatheidae
Munida taenia|AY351004|Galatheidae
Munida taenia|AY351003|Galatheidae
Munida taenia|AY351008|Galatheidae
Munida notata|AY350976|Galatheidae
Munida notata|AY350977|Galatheidae
Munida notata|AY350978|Galatheidae
Munida notata|AY350979|Galatheidae
Munida leagora|AY350963|Galatheidae
Munida leagora|AY350964|Galatheidae
Munida leagora|AY350965|Galatheidae
Munida leagora|AY350966|Galatheidae
Munida leagora|AY350967|Galatheidae
Munida leagora|AY350968|Galatheidae
Munida leagora|AY350969|Galatheidae
Munida leagora|AY350970|Galatheidae
Munida leagora|AY350971|Galatheidae
Munida tyche|AY351016|Galatheidae
Munida tyche|AY351017|Galatheidae
Munida rugosa|FCDOPB084-04|Galatheidae
Munida leptosyne|AY350973|Galatheidae
Shinkaia crosnieri|EU420129|Galatheidae
Shinkaia crosnieri|NC_011013|Galatheidae
Munida ofella|AY350980|Galatheidae
Munida rutllanti|FCFOPC045-03|Galatheidae
Munida rutllanti|FCFOPC045-05|Galatheidae
Munida rutllanti|FCFOPC045-02|Galatheidae
Munida rutllanti|FCFOPC045-04|Galatheidae
Munida rutllanti|JSDMe53|Galatheidae
Munida rutllanti|FCFOPC045-01|Galatheidae
Munida rutllanti|JSDMe54|Galatheidae
Cervimunida johni|AY351054|Galatheidae
Munida subrugosa|AY700169|Galatheidae
Munida gregaria|AY700165|Galatheidae
Munida subrugosa|AY700173|Galatheidae
Munida subrugosa|AY700174|Galatheidae
Munida subrugosa|AY700166|Galatheidae
Munida subrugosa|AY700171|Galatheidae
Munida gregaria|AY700164|Galatheidae
Munida subrugosa|AY700175|Galatheidae
Munida subrugosa|AY700172|Galatheidae
Munida subrugosa|AY700168|Galatheidae
Munida gregaria|AY700163|Galatheidae
Munida subrugosa|AY700167|Galatheidae
Munida subrugosa|AY700176|Galatheidae
Munida quadrispina|FC-VSA135A|Galatheidae
Munida quadrispina|FC-VSA141A|Galatheidae
Munida quadrispina|FC-VSA6|Galatheidae
Pleuroncodes monodon|AY351062|Galatheidae
Agononida sphecia|AY350922|Galatheidae
Agononida sphecia|AY350919|Galatheidae
Agononida sphecia|AY350918|Galatheidae
Agononida sphecia|AY350920|Galatheidae
Agononida sphecia|AY350921|Galatheidae
Agononida sphecia|AY350923|Galatheidae
Agononida marini|AY350914|Galatheidae
Bathymunida nebulosa|AY351052|Galatheidae
Bathymunida nebulosa|AY351053|Galatheidae
Paramunida pronoe|AY351040|Galatheidae
Paramunida labis|AY351032|Galatheidae
Paramunida labis|AY351033|Galatheidae
Paramunida pictura|AY351037|Galatheidae
Paramunida pictura|AY351036|Galatheidae
Paramunida pictura|AY351038|Galatheidae
Paramunida pictura|AY351039|Galatheidae
Paramunida stichas|AY351041|Galatheidae
Paramunida stichas|AY351042|Galatheidae
Paramunida stichas|AY351043|Galatheidae
Paramunida belone|AY351030|Galatheidae
Paramunida luminata|AY351034|Galatheidae
Paramunida luminata|AY351035|Galatheidae
Paramunida thalie|AY351046|Galatheidae
Paramunida thalie|AY351044|Galatheidae
Paramunida thalie|AY351045|Galatheidae
Paramunida thalie|AY351047|Galatheidae
Crosnierita dicata|AY350924|Galatheidae
Agononida procera|AY350916|Galatheidae
Agononida procera|AY350917|Galatheidae
Agononida similis|AY350915|Galatheidae
Onconida alaini|AY351056|Galatheidae
Onconida alaini|AY351057|Galatheidae
Onconida alaini|AY351058|Galatheidae
Onconida tropis|AY351059|Galatheidae
Onconida tropis|AY351060|Galatheidae
Agononida incerta|AF283888|Galatheidae
Agononida incerta|AF283889|Galatheidae
Plesionida aliena|AY351061|Galatheidae
Munida intermedia|FCDPHM52_118|Galatheidae
Munida pagesi|AY350985|Galatheidae
Munida eclepsis|AY350951|Galatheidae
Munida eclepsis|AY350952|Galatheidae
Munida eclepsis|AY350953|Galatheidae
Munida militaris|AY350975|Galatheidae
Munida rhodonia|AF283885|Galatheidae
Munida rhodonia|AF283886|Galatheidae
Munida congesta|AY350945|Galatheidae
Munida rosula|AY350994|Galatheidae
Munida compressa|AY350943|Galatheidae

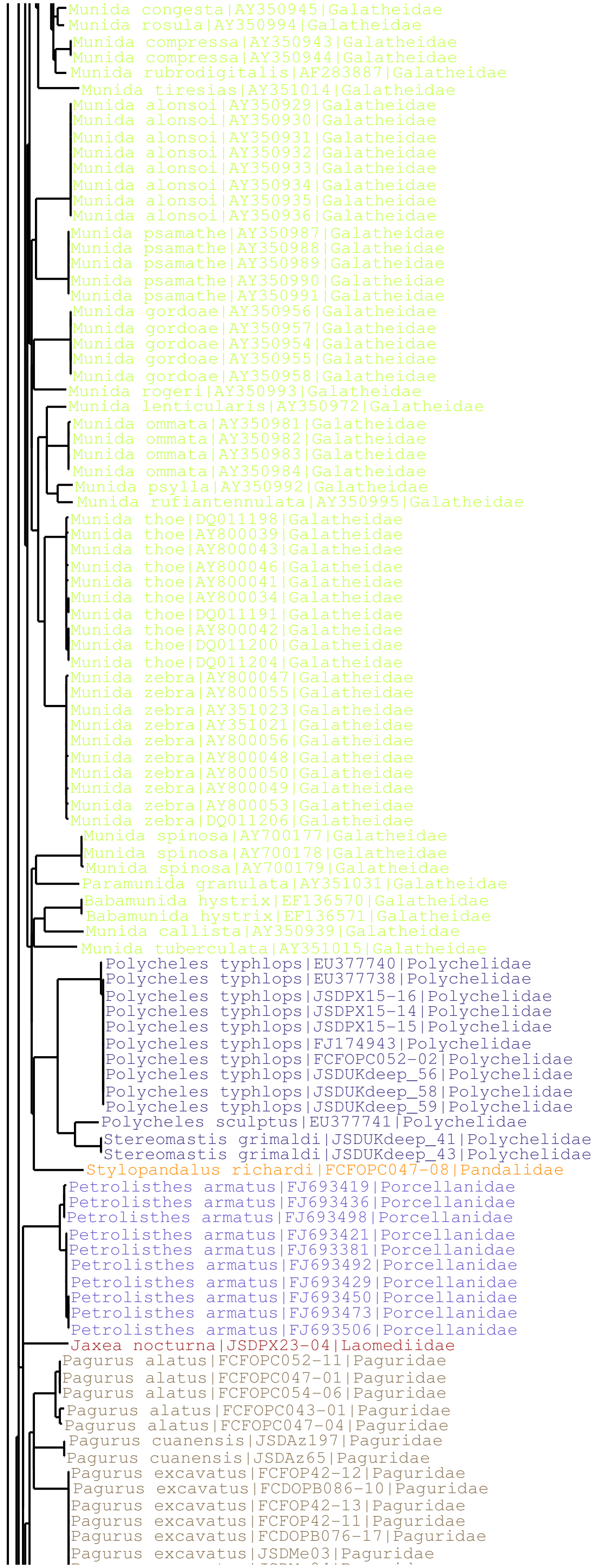
Munida congesta|AY350945|Galatheidae
Munida rosula|AY350994|Galatheidae
Munida compressa|AY350943|Galatheidae
Munida compressa|AY350944|Galatheidae
Munida rubrodigitalis|AF283887|Galatheidae
Munida tiresias|AY351014|Galatheidae
Munida alonsoi|AY350929|Galatheidae
Munida alonsoi|AY350930|Galatheidae
Munida alonsoi|AY350931|Galatheidae
Munida alonsoi|AY350932|Galatheidae
Munida alonsoi|AY350933|Galatheidae
Munida alonsoi|AY350934|Galatheidae
Munida alonsoi|AY350935|Galatheidae
Munida alonsoi|AY350936|Galatheidae
Munida psamathe|AY350987|Galatheidae
Munida psamathe|AY350988|Galatheidae
Munida psamathe|AY350989|Galatheidae
Munida psamathe|AY350990|Galatheidae
Munida psamathe|AY350991|Galatheidae
Munida gordoae|AY350956|Galatheidae
Munida gordoae|AY350957|Galatheidae
Munida gordoae|AY350954|Galatheidae
Munida gordoae|AY350955|Galatheidae
Munida gordoae|AY350958|Galatheidae
Munida rogeri|AY350993|Galatheidae
Munida lenticularis|AY350972|Galatheidae
Munida ommata|AY350981|Galatheidae
Munida ommata|AY350982|Galatheidae
Munida ommata|AY350983|Galatheidae
Munida ommata|AY350984|Galatheidae
Munida psylla|AY350992|Galatheidae
Munida rufiantennulata|AY350995|Galatheidae
Munida thoe|DQ011198|Galatheidae
Munida thoe|AY800039|Galatheidae
Munida thoe|AY800043|Galatheidae
Munida thoe|AY800046|Galatheidae
Munida thoe|AY800041|Galatheidae
Munida thoe|AY800034|Galatheidae
Munida thoe|DQ011191|Galatheidae
Munida thoe|AY800042|Galatheidae
Munida thoe|DQ011200|Galatheidae
Munida thoe|DQ011204|Galatheidae
Munida zebra|AY800047|Galatheidae
Munida zebra|AY800055|Galatheidae
Munida zebra|AY351023|Galatheidae
Munida zebra|AY351021|Galatheidae
Munida zebra|AY800056|Galatheidae
Munida zebra|AY800048|Galatheidae
Munida zebra|AY800050|Galatheidae
Munida zebra|AY800049|Galatheidae
Munida zebra|AY800053|Galatheidae
Munida zebra|DQ011206|Galatheidae
Munida spinosa|AY700177|Galatheidae
Munida spinosa|AY700178|Galatheidae
Munida spinosa|AY700179|Galatheidae
Paramunida granulata|AY351031|Galatheidae
Babamunida hystrix|EF136570|Galatheidae
Babamunida hystrix|EF136571|Galatheidae
Munida callista|AY350939|Galatheidae
Munida tuberculata|AY351015|Galatheidae
Polycheles typhlops|EU377740|Polychelidae
Polycheles typhlops|EU377738|Polychelidae
Polycheles typhlops|JSDPX15-16|Polychelidae
Polycheles typhlops|JSDPX15-14|Polychelidae
Polycheles typhlops|JSDPX15-15|Polychelidae
Polycheles typhlops|FJ174943|Polychelidae
Polycheles typhlops|FCFOPC052-02|Polychelidae
Polycheles typhlops|JSDUKdeep_56|Polychelidae
Polycheles typhlops|JSDUKdeep_58|Polychelidae
Polycheles typhlops|JSDUKdeep_59|Polychelidae
Polycheles sculptus|EU377741|Polychelidae
Stereomastis grimaldi|JSDUKdeep_41|Polychelidae
Stereomastis grimaldi|JSDUKdeep_43|Polychelidae
Stylopandalus richardi|FCFOPC047-08|Pandalidae
Petrolisthes armatus|FJ693419|Porcellanidae
Petrolisthes armatus|FJ693436|Porcellanidae
Petrolisthes armatus|FJ693498|Porcellanidae
Petrolisthes armatus|FJ693421|Porcellanidae
Petrolisthes armatus|FJ693381|Porcellanidae
Petrolisthes armatus|FJ693492|Porcellanidae
Petrolisthes armatus|FJ693429|Porcellanidae
Petrolisthes armatus|FJ693450|Porcellanidae
Petrolisthes armatus|FJ693473|Porcellanidae
Petrolisthes armatus|FJ693506|Porcellanidae
Jaxea nocturna|JSDPX23-04|Laomediidae
Pagurus alatus|FCFOPC052-11|Paguridae
Pagurus alatus|FCFOPC047-01|Paguridae
Pagurus alatus|FCFOPC054-06|Paguridae
Pagurus alatus|FCFOPC043-01|Paguridae
Pagurus alatus|FCFOPC047-04|Paguridae
Pagurus cuanensis|JSDAz197|Paguridae
Pagurus cuanensis|JSDAz65|Paguridae
Pagurus excavatus|FCFOP42-12|Paguridae
Pagurus excavatus|FCDOPB086-10|Paguridae
Pagurus excavatus|FCFOP42-13|Paguridae
Pagurus excavatus|FCFOP42-11|Paguridae
Pagurus excavatus|FCDOPB076-17|Paguridae
Pagurus excavatus|JSDMe03|Paguridae

Pagurus excavatus|FCFOP42-11|Paguridae
Pagurus excavatus|FCDOPB076-17|Paguridae
Pagurus excavatus|JSDMe03|Paguridae
Pagurus excavatus|JSDMe04|Paguridae
Pagurus excavatus|JSDMe05|Paguridae
Pagurus prideauxi|FCDOPB071-10|Paguridae
Pagurus prideauxi|FCFOP42-14|Paguridae
Pagurus prideauxi|JSDN09|Paguridae
Pagurus prideauxi|JSDUK147|Paguridae
Pagurus prideauxi|JSDN11|Paguridae
Pagurus prideauxi|FCFOP42-18|Paguridae
Pagurus prideauxi|FCDOPB074-06|Paguridae
Pagurus prideauxi|FCDOPB071-12|Paguridae
Pagurus prideauxi|FCDOPB071-8|Paguridae
Pagurus prideauxi|JSDUK159|Paguridae
Upogebia africana|DQ351382|Upogebiidae
Upogebia africana|DQ351380|Upogebiidae
Upogebia africana|DQ351379|Upogebiidae
Upogebia africana|DQ351381|Upogebiidae
Upogebia africana|DQ351383|Upogebiidae
Upogebia africana|DQ351384|Upogebiidae
Upogebia africana|DQ351385|Upogebiidae
Upogebia africana|DQ351386|Upogebiidae
Upogebia africana|DQ351387|Upogebiidae
Upogebia africana|DQ351388|Upogebiidae
Clibanarius erythropus|JSDAz115|Diogenidae
Clibanarius erythropus|JSDMe63|Diogenidae
Clibanarius erythropus|JSDMe66|Diogenidae
Clibanarius erythropus|JSDMe67|Diogenidae
Pagurus longicarpus|L209AR1-01|Paguridae
Pagurus longicarpus|L154AR1-08|Paguridae
Pagurus longicarpus|L190AR1-01|Paguridae
Pagurus longicarpus|L183AR1-03|Paguridae
Pagurus longicarpus|L190AR1-02|Paguridae
Pagurus longicarpus|L183AR1-02|Paguridae
Pagurus longicarpus|L210AR4-04|Paguridae
Pagurus longicarpus|L228AR1-01|Paguridae
Eumunida laevimana|EU243497|Chirostylidae
Eumunida laevimana|EU243508|Chirostylidae
Eumunida laevimana|EU243509|Chirostylidae
Eumunida laevimana|EU243510|Chirostylidae
Eumunida marginata|EU243543|Chirostylidae
Eumunida capillata|EU243341|Chirostylidae
Eumunida capillata|EU243342|Chirostylidae
Eumunida capillata|EU243343|Chirostylidae
Eumunida minor|EU243549|Chirostylidae
Eumunida minor|EU243547|Chirostylidae
Eumunida minor|EU243551|Chirostylidae
Eumunida minor|EU243550|Chirostylidae
Eumunida minor|EU243502|Chirostylidae
Eumunida minor|EU243552|Chirostylidae
Eumunida minor|EU243553|Chirostylidae
Eumunida minor|EU243548|Chirostylidae
Eumunida minor|EU243554|Chirostylidae
Eumunida annulosa|AY800019|Chirostylidae
Eumunida annulosa|EU243412|Chirostylidae
Eumunida annulosa|EU243461|Chirostylidae
Eumunida annulosa|DQ011186|Chirostylidae
Eumunida annulosa|DQ011185|Chirostylidae
Eumunida annulosa|EU243357|Chirostylidae
Eumunida annulosa|AY800021|Chirostylidae
Eumunida annulosa|EU243361|Chirostylidae
Eumunida annulosa|EU243470|Chirostylidae
Eumunida annulosa|EU243496|Chirostylidae
Eumunida multilineata|EU243546|Chirostylidae
Eumunida picta|EU243557|Chirostylidae
Eumunida picta|EU243558|Chirostylidae
Eumunida picta|EU243556|Chirostylidae
Eumunida similior|EU243498|Chirostylidae
Eumunida squamifera|EU243559|Chirostylidae
Eumunida squamifera|EU243560|Chirostylidae
Eumunida spinosa|EU243535|Chirostylidae
Eumunida spinosa|EU243534|Chirostylidae
Eumunida spinosa|EU243501|Chirostylidae
Eumunida spinosa|EU243537|Chirostylidae
Eumunida spinosa|EU243500|Chirostylidae
Eumunida spinosa|EU243540|Chirostylidae
Eumunida spinosa|EU243539|Chirostylidae
Eumunida spinosa|EU243536|Chirostylidae
Eumunida spinosa|EU243533|Chirostylidae
Eumunida spinosa|EU243542|Chirostylidae
Eumunida keijii|EU243337|Chirostylidae
Eumunida keijii|EU243338|Chirostylidae
Eumunida keijii|EU243340|Chirostylidae
Eumunida keijii|EU243514|Chirostylidae
Eumunida sternomaculata|EU243458|Chirostylidae
Eumunida sternomaculata|EU243476|Chirostylidae
Eumunida sternomaculata|EU243396|Chirostylidae
Eumunida sternomaculata|EU243450|Chirostylidae
Eumunida sternomaculata|AY800028|Chirostylidae
Eumunida sternomaculata|EU243426|Chirostylidae
Eumunida sternomaculata|AY800029|Chirostylidae
Eumunida sternomaculata|EU243433|Chirostylidae
Eumunida sternomaculata|EU243425|Chirostylidae
Eumunida sternomaculata|EU243483|Chirostylidae
Eumunida treguieri|EU243352|Chirostylidae
Eumunida treguieri|EU243358|Chirostylidae
Eumunida treguieri|EU243516|Chirostylidae
Eumunida treguieri|EU243359|Chirostylidae

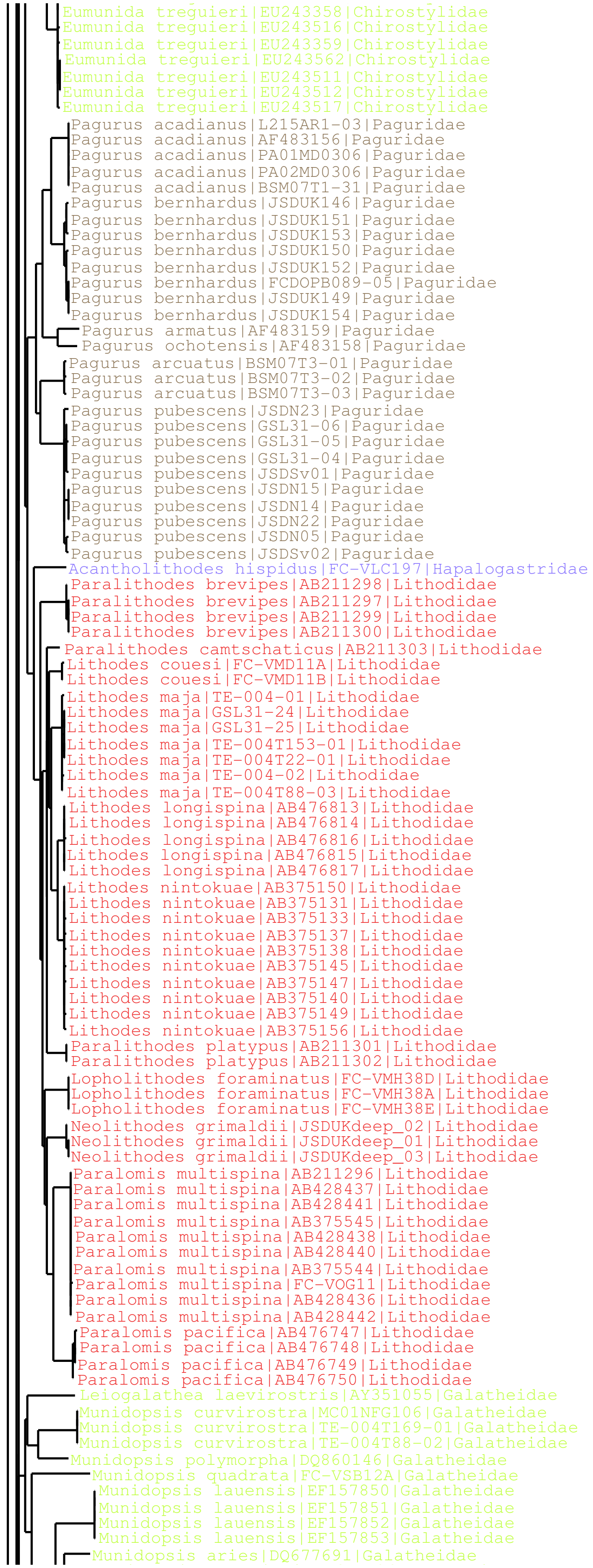
Eumunida treguieri|EU243358|Chirostylidae
Eumunida treguieri|EU243516|Chirostylidae
Eumunida treguieri|EU243359|Chirostylidae
Eumunida treguieri|EU243562|Chirostylidae
Eumunida treguieri|EU243511|Chirostylidae
Eumunida treguieri|EU243512|Chirostylidae
Eumunida treguieri|EU243517|Chirostylidae
Pagurus acadianus|L215AR1-03|Paguridae
Pagurus acadianus|AF483156|Paguridae
Pagurus acadianus|PA01MD0306|Paguridae
Pagurus acadianus|PA02MD0306|Paguridae
Pagurus acadianus|BSM07T1-31|Paguridae
Pagurus bernhardus|JSDUK146|Paguridae
Pagurus bernhardus|JSDUK151|Paguridae
Pagurus bernhardus|JSDUK153|Paguridae
Pagurus bernhardus|JSDUK150|Paguridae
Pagurus bernhardus|JSDUK152|Paguridae
Pagurus bernhardus|FCDOPB089-05|Paguridae
Pagurus bernhardus|JSDUK149|Paguridae
Pagurus bernhardus|JSDUK154|Paguridae
Pagurus armatus|AF483159|Paguridae
Pagurus ochotensis|AF483158|Paguridae
Pagurus arcuatus|BSM07T3-01|Paguridae
Pagurus arcuatus|BSM07T3-02|Paguridae
Pagurus arcuatus|BSM07T3-03|Paguridae
Pagurus pubescens|JSDN23|Paguridae
Pagurus pubescens|GSL31-06|Paguridae
Pagurus pubescens|GSL31-05|Paguridae
Pagurus pubescens|GSL31-04|Paguridae
Pagurus pubescens|JSDSv01|Paguridae
Pagurus pubescens|JSDN15|Paguridae
Pagurus pubescens|JSDN14|Paguridae
Pagurus pubescens|JSDN22|Paguridae
Pagurus pubescens|JSDN05|Paguridae
Pagurus pubescens|JSDSv02|Paguridae
Acantholithodes hispidus|FC-VLC197|Hapalogastridae
Paralithodes brevipes|AB211298|Lithodidae
Paralithodes brevipes|AB211297|Lithodidae
Paralithodes brevipes|AB211299|Lithodidae
Paralithodes brevipes|AB211300|Lithodidae
Paralithodes camtschaticus|AB211303|Lithodidae
Lithodes couesi|FC-VMD11A|Lithodidae
Lithodes couesi|FC-VMD11B|Lithodidae
Lithodes maja|TE-004-01|Lithodidae
Lithodes maja|GSL31-24|Lithodidae
Lithodes maja|GSL31-25|Lithodidae
Lithodes maja|TE-004T153-01|Lithodidae
Lithodes maja|TE-004T22-01|Lithodidae
Lithodes maja|TE-004-02|Lithodidae
Lithodes maja|TE-004T88-03|Lithodidae
Lithodes longispina|AB476813|Lithodidae
Lithodes longispina|AB476814|Lithodidae
Lithodes longispina|AB476816|Lithodidae
Lithodes longispina|AB476815|Lithodidae
Lithodes longispina|AB476817|Lithodidae
Lithodes nintokuae|AB375150|Lithodidae
Lithodes nintokuae|AB375131|Lithodidae
Lithodes nintokuae|AB375133|Lithodidae
Lithodes nintokuae|AB375137|Lithodidae
Lithodes nintokuae|AB375138|Lithodidae
Lithodes nintokuae|AB375145|Lithodidae
Lithodes nintokuae|AB375147|Lithodidae
Lithodes nintokuae|AB375140|Lithodidae
Lithodes nintokuae|AB375149|Lithodidae
Lithodes nintokuae|AB375156|Lithodidae
Paralithodes platypus|AB211301|Lithodidae
Paralithodes platypus|AB211302|Lithodidae
Lopholithodes foraminatus|FC-VMH38D|Lithodidae
Lopholithodes foraminatus|FC-VMH38A|Lithodidae
Lopholithodes foraminatus|FC-VMH38E|Lithodidae
Neolithodes grimaldii|JSDUKdeep_02|Lithodidae
Neolithodes grimaldii|JSDUKdeep_01|Lithodidae
Neolithodes grimaldii|JSDUKdeep_03|Lithodidae
Paralomis multispina|AB211296|Lithodidae
Paralomis multispina|AB428437|Lithodidae
Paralomis multispina|AB428441|Lithodidae
Paralomis multispina|AB375545|Lithodidae
Paralomis multispina|AB428438|Lithodidae
Paralomis multispina|AB428440|Lithodidae
Paralomis multispina|AB375544|Lithodidae
Paralomis multispina|FC-VOG11|Lithodidae
Paralomis multispina|AB428436|Lithodidae
Paralomis multispina|AB428442|Lithodidae
Paralomis pacifica|AB476747|Lithodidae
Paralomis pacifica|AB476748|Lithodidae
Paralomis pacifica|AB476749|Lithodidae
Paralomis pacifica|AB476750|Lithodidae
Leiogalathea laevirostris|AY351055|Galatheidae
Munidopsis curvirostra|MC01NFG106|Galatheidae
Munidopsis curvirostra|TE-004T169-01|Galatheidae
Munidopsis curvirostra|TE-004T88-02|Galatheidae
Munidopsis polymorpha|DQ860146|Galatheidae
Munidopsis quadrata|FC-VSB12A|Galatheidae
Munidopsis lauensis|EF157850|Galatheidae
Munidopsis lauensis|EF157851|Galatheidae
Munidopsis lauensis|EF157852|Galatheidae
Munidopsis lauensis|EF157853|Galatheidae
Munidopsis aries|DQ677691|Galatheidae

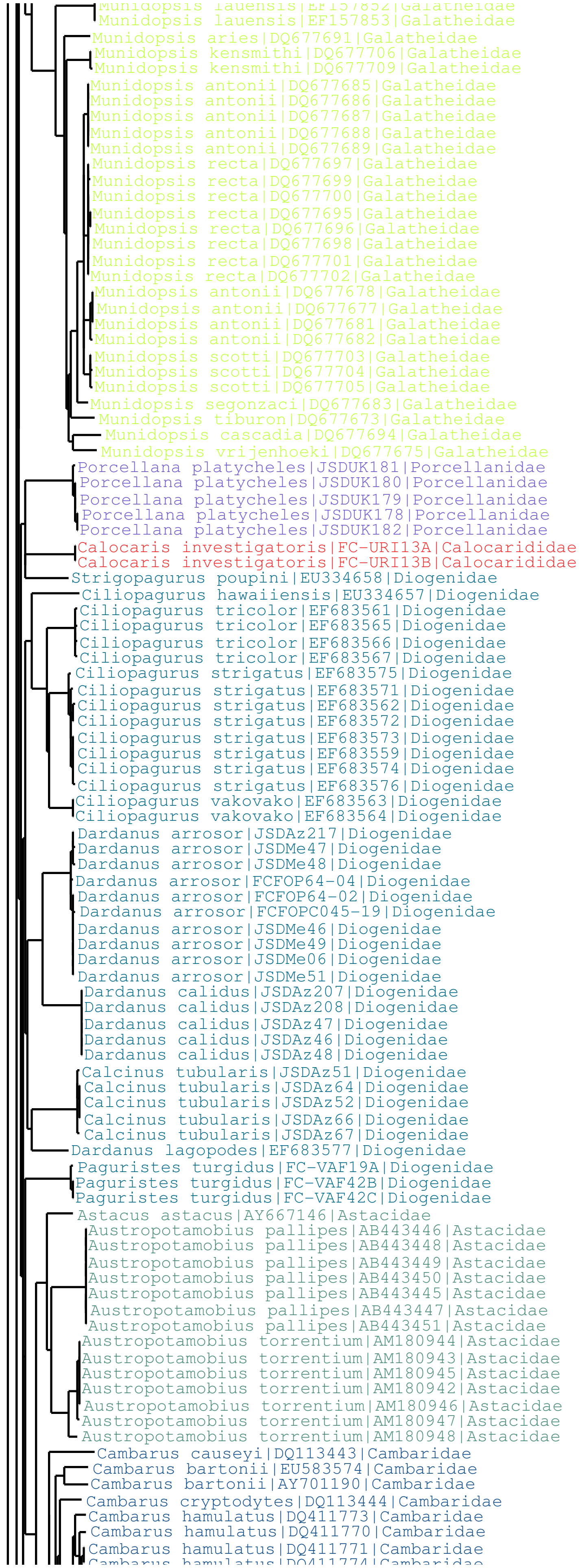
Munidopsis lauensis|EF157852|Galatheidae
Munidopsis lauensis|EF157853|Galatheidae
Munidopsis aries|DQ677691|Galatheidae
Munidopsis kensmithi|DQ677706|Galatheidae
Munidopsis kensmithi|DQ677709|Galatheidae
Munidopsis antonii|DQ677685|Galatheidae
Munidopsis antonii|DQ677686|Galatheidae
Munidopsis antonii|DQ677687|Galatheidae
Munidopsis antonii|DQ677688|Galatheidae
Munidopsis antonii|DQ677689|Galatheidae
Munidopsis recta|DQ677697|Galatheidae
Munidopsis recta|DQ677699|Galatheidae
Munidopsis recta|DQ677700|Galatheidae
Munidopsis recta|DQ677695|Galatheidae
Munidopsis recta|DQ677696|Galatheidae
Munidopsis recta|DQ677698|Galatheidae
Munidopsis recta|DQ677701|Galatheidae
Munidopsis recta|DQ677702|Galatheidae
Munidopsis antonii|DQ677678|Galatheidae
Munidopsis antonii|DQ677677|Galatheidae
Munidopsis antonii|DQ677681|Galatheidae
Munidopsis antonii|DQ677682|Galatheidae
Munidopsis scotti|DQ677703|Galatheidae
Munidopsis scotti|DQ677704|Galatheidae
Munidopsis scotti|DQ677705|Galatheidae
Munidopsis segonzaci|DQ677683|Galatheidae
Munidopsis tiburon|DQ677673|Galatheidae
Munidopsis cascadia|DQ677694|Galatheidae
Munidopsis vrijenhoeki|DQ677675|Galatheidae
Porcellana platycheles|JSDUK181|Porcellanidae
Porcellana platycheles|JSDUK180|Porcellanidae
Porcellana platycheles|JSDUK179|Porcellanidae
Porcellana platycheles|JSDUK178|Porcellanidae
Porcellana platycheles|JSDUK182|Porcellanidae
Calocaris investigatoris|FC-URI13A|Calocarididae
Calocaris investigatoris|FC-URI13B|Calocarididae
Strigopagurus poupini|EU334658|Diogenidae
Ciliopagurus hawaiiensis|EU334657|Diogenidae
Ciliopagurus tricolor|EF683561|Diogenidae
Ciliopagurus tricolor|EF683565|Diogenidae
Ciliopagurus tricolor|EF683566|Diogenidae
Ciliopagurus tricolor|EF683567|Diogenidae
Ciliopagurus strigatus|EF683575|Diogenidae
Ciliopagurus strigatus|EF683571|Diogenidae
Ciliopagurus strigatus|EF683562|Diogenidae
Ciliopagurus strigatus|EF683572|Diogenidae
Ciliopagurus strigatus|EF683573|Diogenidae
Ciliopagurus strigatus|EF683559|Diogenidae
Ciliopagurus strigatus|EF683574|Diogenidae
Ciliopagurus strigatus|EF683576|Diogenidae
Ciliopagurus vakovako|EF683563|Diogenidae
Ciliopagurus vakovako|EF683564|Diogenidae
Dardanus arrosor|JSDAz217|Diogenidae
Dardanus arrosor|JSDMe47|Diogenidae
Dardanus arrosor|JSDMe48|Diogenidae
Dardanus arrosor|FCFOP64-04|Diogenidae
Dardanus arrosor|FCFOP64-02|Diogenidae
Dardanus arrosor|FCFOPC045-19|Diogenidae
Dardanus arrosor|JSDMe46|Diogenidae
Dardanus arrosor|JSDMe49|Diogenidae
Dardanus arrosor|JSDMe06|Diogenidae
Dardanus arrosor|JSDMe51|Diogenidae
Dardanus calidus|JSDAz207|Diogenidae
Dardanus calidus|JSDAz208|Diogenidae
Dardanus calidus|JSDAz47|Diogenidae
Dardanus calidus|JSDAz46|Diogenidae
Dardanus calidus|JSDAz48|Diogenidae
Calcinus tubularis|JSDAz51|Diogenidae
Calcinus tubularis|JSDAz64|Diogenidae
Calcinus tubularis|JSDAz52|Diogenidae
Calcinus tubularis|JSDAz66|Diogenidae
Calcinus tubularis|JSDAz67|Diogenidae
Dardanus lagopodes|EF683577|Diogenidae
Paguristes turgidus|FC-VAF19A|Diogenidae
Paguristes turgidus|FC-VAF42B|Diogenidae
Paguristes turgidus|FC-VAF42C|Diogenidae
Astacus astacus|AY667146|Astacidae
Austropotamobius pallipes|AB443446|Astacidae
Austropotamobius pallipes|AB443448|Astacidae
Austropotamobius pallipes|AB443449|Astacidae
Austropotamobius pallipes|AB443450|Astacidae
Austropotamobius pallipes|AB443445|Astacidae
Austropotamobius pallipes|AB443447|Astacidae
Austropotamobius pallipes|AB443451|Astacidae
Austropotamobius torrentium|AM180944|Astacidae
Austropotamobius torrentium|AM180943|Astacidae
Austropotamobius torrentium|AM180945|Astacidae
Austropotamobius torrentium|AM180942|Astacidae
Austropotamobius torrentium|AM180946|Astacidae
Austropotamobius torrentium|AM180947|Astacidae
Austropotamobius torrentium|AM180948|Astacidae
Cambarus causeyi|DQ113443|Cambaridae
Cambarus bartonii|EU583574|Cambaridae
Cambarus bartonii|AY701190|Cambaridae
Cambarus cryptodytes|DQ113444|Cambaridae
Cambarus hamulatus|DQ411773|Cambaridae
Cambarus hamulatus|DQ411770|Cambaridae
Cambarus hamulatus|DQ411771|Cambaridae
Cambarus hamulatus|DQ411774|Cambaridae

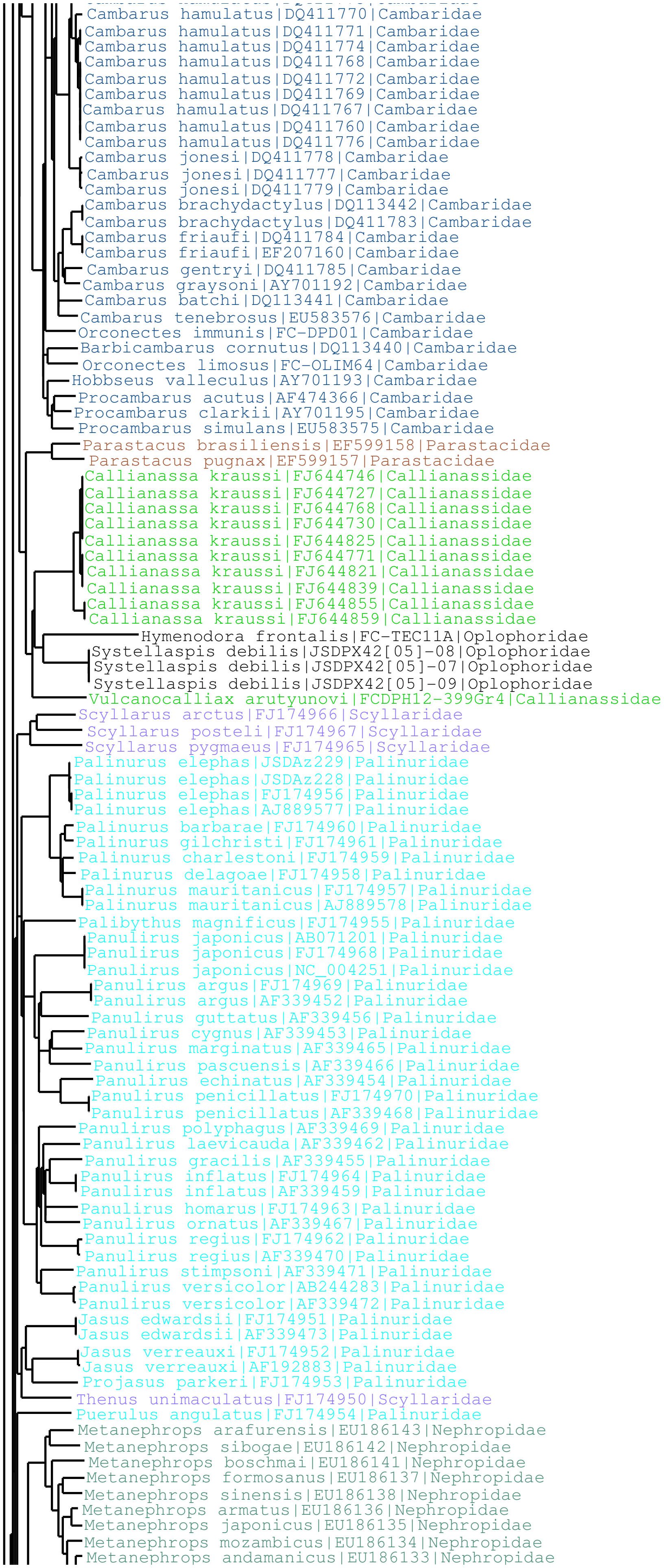
Cambarus hamulatus|DQ411770|Cambaridae
Cambarus hamulatus|DQ411771|Cambaridae
Cambarus hamulatus|DQ411774|Cambaridae
Cambarus hamulatus|DQ411768|Cambaridae
Cambarus hamulatus|DQ411772|Cambaridae
Cambarus hamulatus|DQ411769|Cambaridae
Cambarus hamulatus|DQ411767|Cambaridae
Cambarus hamulatus|DQ411760|Cambaridae
Cambarus hamulatus|DQ411776|Cambaridae
Cambarus jonesi|DQ411778|Cambaridae
Cambarus jonesi|DQ411777|Cambaridae
Cambarus jonesi|DQ411779|Cambaridae
Cambarus brachydactylus|DQ113442|Cambaridae
Cambarus brachydactylus|DQ411783|Cambaridae
Cambarus friaufi|DQ411784|Cambaridae
Cambarus friaufi|EF207160|Cambaridae
Cambarus gentryi|DQ411785|Cambaridae
Cambarus graysoni|AY701192|Cambaridae
Cambarus batchi|DQ113441|Cambaridae
Cambarus tenebrosus|EU583576|Cambaridae
Orconectes immunis|FC-DPD01|Cambaridae
Barbicambarus cornutus|DQ113440|Cambaridae
Orconectes limosus|FC-OLIM64|Cambaridae
Hobbseus valleculus|AY701193|Cambaridae
Procambarus acutus|AF474366|Cambaridae
Procambarus clarkii|AY701195|Cambaridae
Procambarus simulans|EU583575|Cambaridae
Parastacus brasiliensis|EF599158|Parastacidae
Parastacus pugnax|EF599157|Parastacidae
Callianassa kraussi|FJ644746|Callianassidae
Callianassa kraussi|FJ644727|Callianassidae
Callianassa kraussi|FJ644768|Callianassidae
Callianassa kraussi|FJ644730|Callianassidae
Callianassa kraussi|FJ644825|Callianassidae
Callianassa kraussi|FJ644771|Callianassidae
Callianassa kraussi|FJ644821|Callianassidae
Callianassa kraussi|FJ644839|Callianassidae
Callianassa kraussi|FJ644855|Callianassidae
Callianassa kraussi|FJ644859|Callianassidae
Hymenodora frontalis|FC-TEC11A|Oplophoridae
Systellaspis debilis|JSDPX42[05]-08|Oplophoridae
Systellaspis debilis|JSDPX42[05]-07|Oplophoridae
Systellaspis debilis|JSDPX42[05]-09|Oplophoridae
Vulcanocalliax arutyunovi|FCDPH12-399Gr4|Callianassidae
Scyllarus arctus|FJ174966|Scyllaridae
Scyllarus posteli|FJ174967|Scyllaridae
Scyllarus pygmaeus|FJ174965|Scyllaridae
Palinurus elephas|JSDAz229|Palinuridae
Palinurus elephas|JSDAz228|Palinuridae
Palinurus elephas|FJ174956|Palinuridae
Palinurus elephas|AJ889577|Palinuridae
Palinurus barbarae|FJ174960|Palinuridae
Palinurus gilchristi|FJ174961|Palinuridae
Palinurus charlestoni|FJ174959|Palinuridae
Palinurus delagoae|FJ174958|Palinuridae
Palinurus mauritanicus|FJ174957|Palinuridae
Palinurus mauritanicus|AJ889578|Palinuridae
Palibythus magnificus|FJ174955|Palinuridae
Panulirus japonicus|AB071201|Palinuridae
Panulirus japonicus|FJ174968|Palinuridae
Panulirus japonicus|NC_004251|Palinuridae
Panulirus argus|FJ174969|Palinuridae
Panulirus argus|AF339452|Palinuridae
Panulirus guttatus|AF339456|Palinuridae
Panulirus cygnus|AF339453|Palinuridae
Panulirus marginatus|AF339465|Palinuridae
Panulirus pascuensis|AF339466|Palinuridae
Panulirus echinatus|AF339454|Palinuridae
Panulirus penicillatus|FJ174970|Palinuridae
Panulirus penicillatus|AF339468|Palinuridae
Panulirus polyphagus|AF339469|Palinuridae
Panulirus laevicauda|AF339462|Palinuridae
Panulirus gracilis|AF339455|Palinuridae
Panulirus inflatus|FJ174964|Palinuridae
Panulirus inflatus|AF339459|Palinuridae
Panulirus homarus|FJ174963|Palinuridae
Panulirus ornatus|AF339467|Palinuridae
Panulirus regius|FJ174962|Palinuridae
Panulirus regius|AF339470|Palinuridae
Panulirus stimpsoni|AF339471|Palinuridae
Panulirus versicolor|AB244283|Palinuridae
Panulirus versicolor|AF339472|Palinuridae
Jasus edwardsii|FJ174951|Palinuridae
Jasus edwardsii|AF339473|Palinuridae
Jasus verreauxi|FJ174952|Palinuridae
Jasus verreauxi|AF192883|Palinuridae
Projasus parkeri|FJ174953|Palinuridae
Thenus unimaculatus|FJ174950|Scyllaridae
Puerulus angulatus|FJ174954|Palinuridae
Metanephrops arafurensis|EU186143|Nephropidae
Metanephrops sibogae|EU186142|Nephropidae
Metanephrops boschmai|EU186141|Nephropidae
Metanephrops formosanus|EU186137|Nephropidae
Metanephrops sinensis|EU186138|Nephropidae
Metanephrops armatus|EU186136|Nephropidae
Metanephrops japonicus|EU186135|Nephropidae
Metanephrops mozambicus|EU186134|Nephropidae
Metanephrops andamanicus|EU186133|Nephropidae

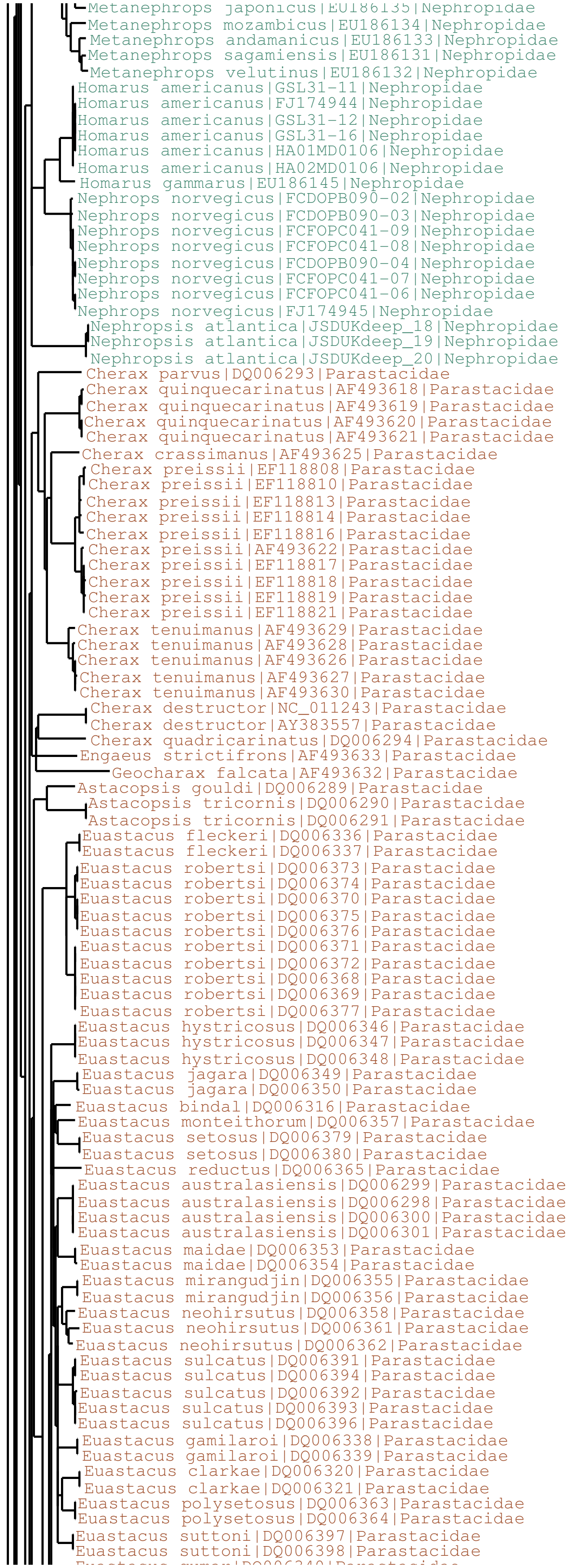
Metanephrops japonicus|EU186135|Nephropidae
Metanephrops mozambicus|EU186134|Nephropidae
Metanephrops andamanicus|EU186133|Nephropidae
Metanephrops sagamiensis|EU186131|Nephropidae
Metanephrops velutinus|EU186132|Nephropidae
Homarus americanus|GSL31-11|Nephropidae
Homarus americanus|FJ174944|Nephropidae
Homarus americanus|GSL31-12|Nephropidae
Homarus americanus|GSL31-16|Nephropidae
Homarus americanus|HA01MD0106|Nephropidae
Homarus americanus|HA02MD0106|Nephropidae
Homarus gammarus|EU186145|Nephropidae
Nephrops norvegicus|FCDOPB090-02|Nephropidae
Nephrops norvegicus|FCDOPB090-03|Nephropidae
Nephrops norvegicus|FCFOPC041-09|Nephropidae
Nephrops norvegicus|FCFOPC041-08|Nephropidae
Nephrops norvegicus|FCDOPB090-04|Nephropidae
Nephrops norvegicus|FCFOPC041-07|Nephropidae
Nephrops norvegicus|FCFOPC041-06|Nephropidae
Nephrops norvegicus|FJ174945|Nephropidae
Nephropsis atlantica|JSDUKdeep_18|Nephropidae
Nephropsis atlantica|JSDUKdeep_19|Nephropidae
Nephropsis atlantica|JSDUKdeep_20|Nephropidae
Cherax parvus|DQ006293|Parastacidae
Cherax quinquecarinatus|AF493618|Parastacidae
Cherax quinquecarinatus|AF493619|Parastacidae
Cherax quinquecarinatus|AF493620|Parastacidae
Cherax quinquecarinatus|AF493621|Parastacidae
Cherax crassimanus|AF493625|Parastacidae
Cherax preissii|EF118808|Parastacidae
Cherax preissii|EF118810|Parastacidae
Cherax preissii|EF118813|Parastacidae
Cherax preissii|EF118814|Parastacidae
Cherax preissii|EF118816|Parastacidae
Cherax preissii|AF493622|Parastacidae
Cherax preissii|EF118817|Parastacidae
Cherax preissii|EF118818|Parastacidae
Cherax preissii|EF118819|Parastacidae
Cherax preissii|EF118821|Parastacidae
Cherax tenuimanus|AF493629|Parastacidae
Cherax tenuimanus|AF493628|Parastacidae
Cherax tenuimanus|AF493626|Parastacidae
Cherax tenuimanus|AF493627|Parastacidae
Cherax tenuimanus|AF493630|Parastacidae
Cherax destructor|NC_011243|Parastacidae
Cherax destructor|AY383557|Parastacidae
Cherax quadricarinatus|DQ006294|Parastacidae
Engaeus strictifrons|AF493633|Parastacidae
Geocharax falcata|AF493632|Parastacidae
Astacopsis gouldi|DQ006289|Parastacidae
Astacopsis tricornis|DQ006290|Parastacidae
Astacopsis tricornis|DQ006291|Parastacidae
Euastacus fleckeri|DQ006336|Parastacidae
Euastacus fleckeri|DQ006337|Parastacidae
Euastacus robertsi|DQ006373|Parastacidae
Euastacus robertsi|DQ006374|Parastacidae
Euastacus robertsi|DQ006370|Parastacidae
Euastacus robertsi|DQ006375|Parastacidae
Euastacus robertsi|DQ006376|Parastacidae
Euastacus robertsi|DQ006371|Parastacidae
Euastacus robertsi|DQ006372|Parastacidae
Euastacus robertsi|DQ006368|Parastacidae
Euastacus robertsi|DQ006369|Parastacidae
Euastacus robertsi|DQ006377|Parastacidae
Euastacus hystricosus|DQ006346|Parastacidae
Euastacus hystricosus|DQ006347|Parastacidae
Euastacus hystricosus|DQ006348|Parastacidae
Euastacus jagara|DQ006349|Parastacidae
Euastacus jagara|DQ006350|Parastacidae
Euastacus bindal|DQ006316|Parastacidae
Euastacus monteithorum|DQ006357|Parastacidae
Euastacus setosus|DQ006379|Parastacidae
Euastacus setosus|DQ006380|Parastacidae
Euastacus reductus|DQ006365|Parastacidae
Euastacus australasiensis|DQ006299|Parastacidae
Euastacus australasiensis|DQ006298|Parastacidae
Euastacus australasiensis|DQ006300|Parastacidae
Euastacus australasiensis|DQ006301|Parastacidae
Euastacus maidae|DQ006353|Parastacidae
Euastacus maidae|DQ006354|Parastacidae
Euastacus mirangudjin|DQ006355|Parastacidae
Euastacus mirangudjin|DQ006356|Parastacidae
Euastacus neohirsutus|DQ006358|Parastacidae
Euastacus neohirsutus|DQ006361|Parastacidae
Euastacus neohirsutus|DQ006362|Parastacidae
Euastacus sulcatus|DQ006391|Parastacidae
Euastacus sulcatus|DQ006394|Parastacidae
Euastacus sulcatus|DQ006392|Parastacidae
Euastacus sulcatus|DQ006393|Parastacidae
Euastacus sulcatus|DQ006396|Parastacidae
Euastacus gamilaroi|DQ006338|Parastacidae
Euastacus gamilaroi|DQ006339|Parastacidae
Euastacus clarkae|DQ006320|Parastacidae
Euastacus clarkae|DQ006321|Parastacidae
Euastacus polysetosus|DQ006363|Parastacidae
Euastacus polysetosus|DQ006364|Parastacidae
Euastacus suttoni|DQ006397|Parastacidae
Euastacus suttoni|DQ006398|Parastacidae

Euastacus polysetosus|DQ00630[illegible]|Parastacidae
Euastacus suttoni|DQ006397|Parastacidae
Euastacus suttoni|DQ006398|Parastacidae
Euastacus gumar|DQ006340|Parastacidae
Euastacus gumar|DQ006341|Parastacidae
Euastacus dangadi|DQ006327|Parastacidae
Euastacus dangadi|DQ006328|Parastacidae
Euastacus valentulus|DQ006401|Parastacidae
Euastacus valentulus|DQ006402|Parastacidae
Euastacus diversus|DQ006331|Parastacidae
Euastacus diversus|DQ006332|Parastacidae
Euastacus crassus|DQ006324|Parastacidae
Euastacus crassus|DQ006326|Parastacidae
Euastacus rieki|DQ006366|Parastacidae
Euastacus rieki|DQ006367|Parastacidae
Euastacus kershawi|DQ006351|Parastacidae
Euastacus kershawi|DQ006352|Parastacidae
Euastacus woiwuru|DQ006403|Parastacidae
Euastacus woiwuru|DQ006404|Parastacidae
Euastacus dharawalus|AY380480|Parastacidae
Euastacus dharawalus|DQ006329|Parastacidae
Euastacus dharawalus|DQ006330|Parastacidae
Euastacus brachythorax|DQ006318|Parastacidae
Euastacus brachythorax|DQ006319|Parastacidae
Euastacus claytoni|AY380479|Parastacidae
Euastacus claytoni|DQ006322|Parastacidae
Euastacus claytoni|DQ006323|Parastacidae
Euastacus guwinus|DQ006343|Parastacidae
Euastacus guwinus|DQ006345|Parastacidae
Euastacus guwinus|DQ006342|Parastacidae
Euastacus guwinus|DQ006344|Parastacidae
Euastacus yanga|DQ006408|Parastacidae
Euastacus yanga|DQ006406|Parastacidae
Euastacus yanga|DQ006405|Parastacidae
Euastacus yanga|DQ006407|Parastacidae
Euastacus yanga|DQ006409|Parastacidae
Euastacus spinifer|DQ006386|Parastacidae
Euastacus spinifer|DQ006389|Parastacidae
Euastacus spinifer|DQ006385|Parastacidae
Euastacus spinifer|DQ006388|Parastacidae
Euastacus spinifer|DQ006387|Parastacidae
Euastacus spinifer|DQ006390|Parastacidae
Euastacus armatus|DQ006295|Parastacidae
Euastacus armatus|DQ006296|Parastacidae
Euastacus armatus|DQ006297|Parastacidae
Euastacus crassus|DQ006325|Parastacidae
Euastacus bispinosus|AF493634|Parastacidae
Euastacus bispinosus|DQ006317|Parastacidae
Euastacus yarraensis|DQ006410|Parastacidae
Euastacus yarraensis|DQ006411|Parastacidae
Euastacus yarraensis|DQ006412|Parastacidae
Euastacus balanensis|DQ006303|Parastacidae
Euastacus balanensis|DQ006302|Parastacidae
Euastacus balanensis|DQ006305|Parastacidae
Euastacus balanensis|DQ006309|Parastacidae
Euastacus balanensis|DQ006304|Parastacidae
Euastacus balanensis|DQ006306|Parastacidae
Euastacus balanensis|DQ006307|Parastacidae
Euastacus balanensis|DQ006308|Parastacidae
Euastacus balanensis|DQ006311|Parastacidae
Euastacus balanensis|DQ006312|Parastacidae
Euastacus yigara|DQ006413|Parastacidae
Euastacus yigara|DQ006414|Parastacidae
Paranephrops planifrons|DQ394241|Parastacidae
Paranephrops planifrons|DQ394184|Parastacidae
Paranephrops planifrons|DQ394235|Parastacidae
Paranephrops planifrons|DQ394219|Parastacidae
Paranephrops planifrons|EF680493|Parastacidae
Paranephrops planifrons|EF680490|Parastacidae
Paranephrops planifrons|EF680491|Parastacidae
Paranephrops planifrons|DQ394182|Parastacidae
Paranephrops planifrons|EF680488|Parastacidae
Paranephrops planifrons|EF680497|Parastacidae
Paranephrops zealandicus|DQ394250|Parastacidae
Paranephrops zealandicus|DQ394254|Parastacidae
Paranephrops zealandicus|DQ394268|Parastacidae
Paranephrops zealandicus|DQ394269|Parastacidae
Paranephrops zealandicus|DQ006417|Parastacidae
Paranephrops zealandicus|DQ394270|Parastacidae
Paranephrops zealandicus|DQ394273|Parastacidae
Paranephrops zealandicus|DQ394277|Parastacidae
Paranephrops zealandicus|DQ394223|Parastacidae
Paranephrops zealandicus|DQ394282|Parastacidae
Samastacus spinifrons|EF599160|Parastacidae
Virilastacus retamali|EF599154|Parastacidae
Virilastacus retamali|EF599155|Parastacidae
Virilastacus rucapihuelensis|EF599150|Parastacidae
Periclimenes soror|FJ386124|Palaemonidae
Periclimenes soror|FJ386233|Palaemonidae
Periclimenes soror|FJ386189|Palaemonidae
Periclimenes soror|FJ386153|Palaemonidae
Periclimenes soror|FJ386174|Palaemonidae
Periclimenes soror|FJ386141|Palaemonidae
Periclimenes soror|FJ386204|Palaemonidae
Periclimenes soror|FJ386217|Palaemonidae
Periclimenes soror|FJ386214|Palaemonidae
Periclimenes soror|FJ386264|Palaemonidae
Periclimenes lamellibrachiophilus|AB470722|Palaemonidae
Periclimenes lamellibrachiophilus|AB470720|Palaemonidae

Periclimenes soror|FJ386264|Palaemonidae
Periclimenes lamellibrachiophilus|AB470722|Palaemonidae
Periclimenes lamellibrachiophilus|AB470720|Palaemonidae
Periclimenes lamellibrachiophilus|AB470727|Palaemonidae
Periclimenes lamellibrachiophilus|AB470730|Palaemonidae
Periclimenes thermohydrophilus|AB298106|Palaemonidae
Periclimenes thermohydrophilus|AB298107|Palaemonidae
Periclimenes thermohydrophilus|AB298102|Palaemonidae
Periclimenes thermohydrophilus|AB298103|Palaemonidae
Periclimenes thermohydrophilus|AB298104|Palaemonidae
Periclimenes thermohydrophilus|AB298105|Palaemonidae
Scyllarides herklotsii|FJ174946|Scyllaridae
Scyllarides latus|JSDAz240|Scyllaridae
Scyllarides latus|FJ174947|Scyllaridae
Scyllarides latus|JSDAz236|Scyllaridae
Scyllarides latus|JSDAz235|Scyllaridae
Scyllarides latus|JSDAz36|Scyllaridae
Scyllarides latus|JSDAz37|Scyllaridae
Sergestes similis|FC-TPD17A|Sergestidae
Sergestes similis|FC-TPD17B|Sergestidae
Sergestes similis|FC-TPD17C|Sergestidae
Sergestes similis|FC-TPD17D|Sergestidae
Sergia robusta|JSDPX71-01|Sergestidae
Sergia robusta|JSDPX4-02|Sergestidae
Sergia robusta|JSDPX4-03|Sergestidae
Sergia robusta|FCFOPC069-03|Sergestidae
Sergia robusta|JSDPX79-05|Sergestidae
Sergia robusta|JSDUKdeep_21|Sergestidae
Sergia robusta|JSDUKdeep_22|Sergestidae
Sergia robusta|JSDUKdeep_23|Sergestidae
Funchalia villose|JSDPX42[05]-01|Penaeidae
Marsupenaeus japonicus|NC_007010|Penaeidae
Marsupenaeus japonicus|AP006346|Penaeidae
Marsupenaeus japonicus|AY787755|Penaeidae
Melicertus kerathurus|JSDPX23-01|Penaeidae
Melicertus kerathurus|EF219326|Penaeidae
Melicertus kerathurus|EF219324|Penaeidae
Melicertus kerathurus|JSDPX23-03|Penaeidae
Melicertus kerathurus|EF219290|Penaeidae
Melicertus kerathurus|EF219289|Penaeidae
Melicertus kerathurus|EF219291|Penaeidae
Melicertus kerathurus|EF219308|Penaeidae
Melicertus kerathurus|EF219309|Penaeidae
Melicertus kerathurus|EF219342|Penaeidae
Farfantepenaeus notialis|X84350 X84351 X84352 X84353 X84354 X84355 X84356 X84357|Penaeidae
Farfantepenaeus notialis|X84350 X84351 X84352 X84353 X84354 X84355 X84356 X84357|Penaeidae
Farfantepenaeus notialis|X84350|Penaeidae
Litopenaeus stylirostris|EU517503|Penaeidae
Litopenaeus stylirostris|NC_012060|Penaeidae
Litopenaeus vannamei|DQ534543|Penaeidae
Fenneropenaeus chinensis|NC_009679|Penaeidae
Fenneropenaeus chinensis|DQ518969|Penaeidae
Fenneropenaeus chinensis|DQ656600|Penaeidae
Penaeus monodon|NC_002184|Penaeidae
Penaeus monodon|AF217843|Penaeidae
Parapenaeus longirostris|FCFOPC042-06|Penaeidae
Parapenaeus longirostris|JSDMe37|Penaeidae
Parapenaeus longirostris|JSDMe38|Penaeidae
Penaeopsis serrata|FCFOPC050-01|Penaeidae
Hymenopenaeus debilis|JSDPX40-01|Solenoceridae
Hymenopenaeus debilis|JSDPX39-02|Solenoceridae
Hymenopenaeus debilis|JSDPX42[05]-04|Solenoceridae
Hymenopenaeus debilis|JSDPX42[05]-05|Solenoceridae
Hymenopenaeus debilis|JSDPX39-01|Solenoceridae
Hymenopenaeus debilis|JSDPX42[05]-06|Solenoceridae
Solenocera membranacea|JSDMe19|Solenoceridae
Solenocera membranacea|FCFOPC041-14|Solenoceridae
Solenocera membranacea|FCFOPC041-13|Solenoceridae
Solenocera membranacea|FCFOP66-11|Solenoceridae
Solenocera membranacea|FCDOPB089-03|Solenoceridae
Solenocera membranacea|FCDOPB089-02|Solenoceridae
Solenocera membranacea|FCDOPB083-07|Solenoceridae
Solenocera membranacea|FCFOPC041-12|Solenoceridae
Solenocera membranacea|JSDMe20|Solenoceridae
Solenocera membranacea|JSDMe21|Solenoceridae
Dichelopandalus bonnieri|JSDUKdeep_51|Pandalidae
Dichelopandalus bonnieri|JSDUKdeep_53|Pandalidae
Pontocaris lacazei|FCFOPC041-51|Crangonidae
Pontocaris lacazei|FCDPH15-575B51|Crangonidae
Pontocaris lacazei|FCFOPC044-07|Crangonidae
Pontocaris lacazei|FCFOPC044-08|Crangonidae
Pontocaris lacazei|FCFOPC044-09|Crangonidae
Crangon alaskensis|FC-SIC201A|Crangonidae
Crangon alaskensis|FC-SIC201B|Crangonidae
Crangon septemspinosa|L210AR4-05|Crangonidae
Crangon septemspinosa|L37AR1-01|Crangonidae
Crangon septemspinosa|L175AR1-02|Crangonidae
Crangon septemspinosa|L154AR1-04|Crangonidae
Crangon septemspinosa|L51AR1-01|Crangonidae
Crangon septemspinosa|L184AR1-07|Crangonidae
Crangon septemspinosa|L187AR1-05|Crangonidae
Crangon septemspinosa|L210AR4-06|Crangonidae
Crangon septemspinosa|L42AR1-01|Crangonidae
Crangon septemspinosa|CS02SL0106|Crangonidae
Argis lar|FC-SFF12A|Crangonidae
Argis lar|FC-SFF21A|Crangonidae
Crangon abyssorum|FC-SIB14A|Crangonidae
Crangon abyssorum|FC-SIB14B|Crangonidae

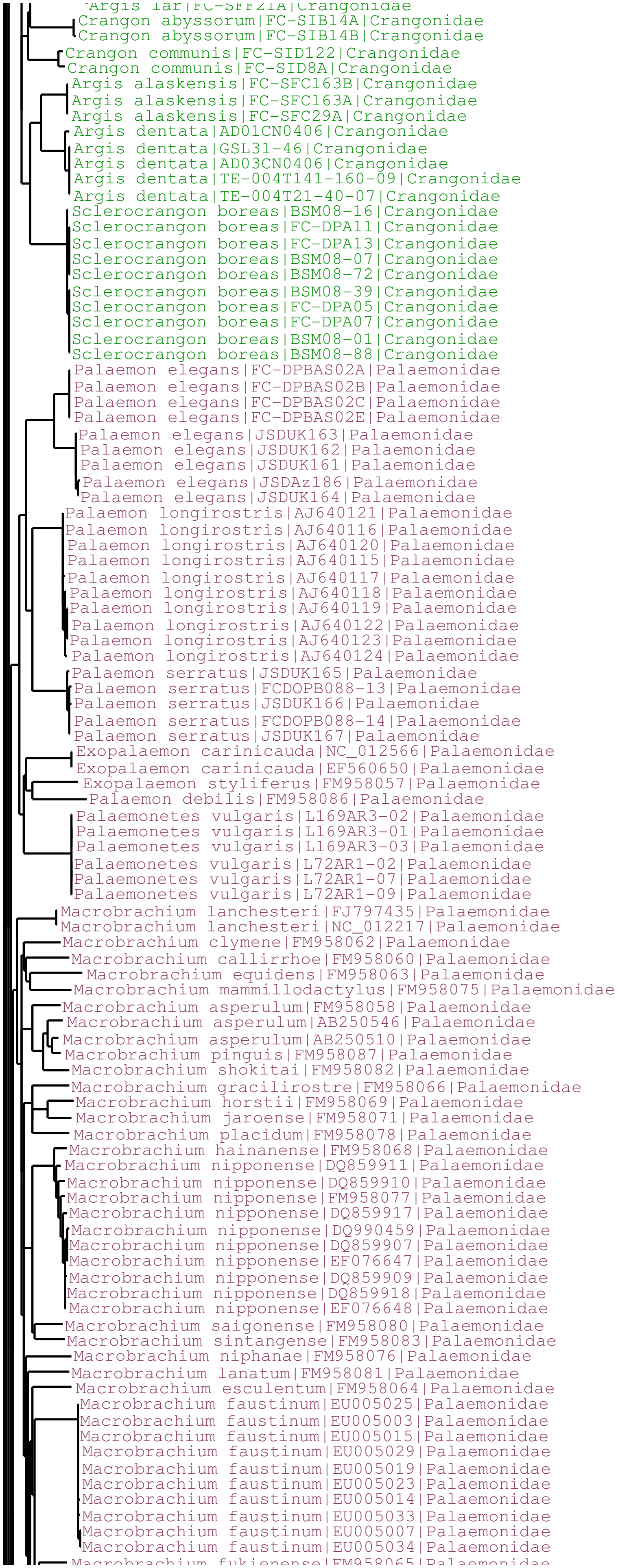
Argis lar|FC-SFF21A|Crangonidae
Crangon abyssorum|FC-SIB14A|Crangonidae
Crangon abyssorum|FC-SIB14B|Crangonidae
Crangon communis|FC-SID122|Crangonidae
Crangon communis|FC-SID8A|Crangonidae
Argis alaskensis|FC-SFC163B|Crangonidae
Argis alaskensis|FC-SFC163A|Crangonidae
Argis alaskensis|FC-SFC29A|Crangonidae
Argis dentata|AD01CN0406|Crangonidae
Argis dentata|GSL31-46|Crangonidae
Argis dentata|AD03CN0406|Crangonidae
Argis dentata|TE-004T141-160-09|Crangonidae
Argis dentata|TE-004T21-40-07|Crangonidae
Sclerocrangon boreas|BSM08-16|Crangonidae
Sclerocrangon boreas|FC-DPA11|Crangonidae
Sclerocrangon boreas|FC-DPA13|Crangonidae
Sclerocrangon boreas|BSM08-07|Crangonidae
Sclerocrangon boreas|BSM08-72|Crangonidae
Sclerocrangon boreas|BSM08-39|Crangonidae
Sclerocrangon boreas|FC-DPA05|Crangonidae
Sclerocrangon boreas|FC-DPA07|Crangonidae
Sclerocrangon boreas|BSM08-01|Crangonidae
Sclerocrangon boreas|BSM08-88|Crangonidae
Palaemon elegans|FC-DPBAS02A|Palaemonidae
Palaemon elegans|FC-DPBAS02B|Palaemonidae
Palaemon elegans|FC-DPBAS02C|Palaemonidae
Palaemon elegans|FC-DPBAS02E|Palaemonidae
Palaemon elegans|JSDUK163|Palaemonidae
Palaemon elegans|JSDUK162|Palaemonidae
Palaemon elegans|JSDUK161|Palaemonidae
Palaemon elegans|JSDAz186|Palaemonidae
Palaemon elegans|JSDUK164|Palaemonidae
Palaemon longirostris|AJ640121|Palaemonidae
Palaemon longirostris|AJ640116|Palaemonidae
Palaemon longirostris|AJ640120|Palaemonidae
Palaemon longirostris|AJ640115|Palaemonidae
Palaemon longirostris|AJ640117|Palaemonidae
Palaemon longirostris|AJ640118|Palaemonidae
Palaemon longirostris|AJ640119|Palaemonidae
Palaemon longirostris|AJ640122|Palaemonidae
Palaemon longirostris|AJ640123|Palaemonidae
Palaemon longirostris|AJ640124|Palaemonidae
Palaemon serratus|JSDUK165|Palaemonidae
Palaemon serratus|FCDOPB088-13|Palaemonidae
Palaemon serratus|JSDUK166|Palaemonidae
Palaemon serratus|FCDOPB088-14|Palaemonidae
Palaemon serratus|JSDUK167|Palaemonidae
Exopalaemon carinicauda|NC_012566|Palaemonidae
Exopalaemon carinicauda|EF560650|Palaemonidae
Exopalaemon styliferus|FM958057|Palaemonidae
Palaemon debilis|FM958086|Palaemonidae
Palaemonetes vulgaris|L169AR3-02|Palaemonidae
Palaemonetes vulgaris|L169AR3-01|Palaemonidae
Palaemonetes vulgaris|L169AR3-03|Palaemonidae
Palaemonetes vulgaris|L72AR1-02|Palaemonidae
Palaemonetes vulgaris|L72AR1-07|Palaemonidae
Palaemonetes vulgaris|L72AR1-09|Palaemonidae
Macrobrachium lanchesteri|FJ797435|Palaemonidae
Macrobrachium lanchesteri|NC_012217|Palaemonidae
Macrobrachium clymene|FM958062|Palaemonidae
Macrobrachium callirrhoe|FM958060|Palaemonidae
Macrobrachium equidens|FM958063|Palaemonidae
Macrobrachium mammillodactylus|FM958075|Palaemonidae
Macrobrachium asperulum|FM958058|Palaemonidae
Macrobrachium asperulum|AB250546|Palaemonidae
Macrobrachium asperulum|AB250510|Palaemonidae
Macrobrachium pinguis|FM958087|Palaemonidae
Macrobrachium shokitai|FM958082|Palaemonidae
Macrobrachium gracilirostre|FM958066|Palaemonidae
Macrobrachium horstii|FM958069|Palaemonidae
Macrobrachium jaroense|FM958071|Palaemonidae
Macrobrachium placidum|FM958078|Palaemonidae
Macrobrachium hainanense|FM958068|Palaemonidae
Macrobrachium nipponense|DQ859911|Palaemonidae
Macrobrachium nipponense|DQ859910|Palaemonidae
Macrobrachium nipponense|FM958077|Palaemonidae
Macrobrachium nipponense|DQ859917|Palaemonidae
Macrobrachium nipponense|DQ990459|Palaemonidae
Macrobrachium nipponense|DQ859907|Palaemonidae
Macrobrachium nipponense|EF076647|Palaemonidae
Macrobrachium nipponense|DQ859909|Palaemonidae
Macrobrachium nipponense|DQ859918|Palaemonidae
Macrobrachium nipponense|EF076648|Palaemonidae
Macrobrachium saigonense|FM958080|Palaemonidae
Macrobrachium sintangense|FM958083|Palaemonidae
Macrobrachium niphanae|FM958076|Palaemonidae
Macrobrachium lanatum|FM958081|Palaemonidae
Macrobrachium esculentum|FM958064|Palaemonidae
Macrobrachium faustinum|EU005025|Palaemonidae
Macrobrachium faustinum|EU005003|Palaemonidae
Macrobrachium faustinum|EU005015|Palaemonidae
Macrobrachium faustinum|EU005029|Palaemonidae
Macrobrachium faustinum|EU005019|Palaemonidae
Macrobrachium faustinum|EU005023|Palaemonidae
Macrobrachium faustinum|EU005014|Palaemonidae
Macrobrachium faustinum|EU005033|Palaemonidae
Macrobrachium faustinum|EU005007|Palaemonidae
Macrobrachium faustinum|EU005034|Palaemonidae
Macrobrachium fukienense|FM958065|Palaemonidae

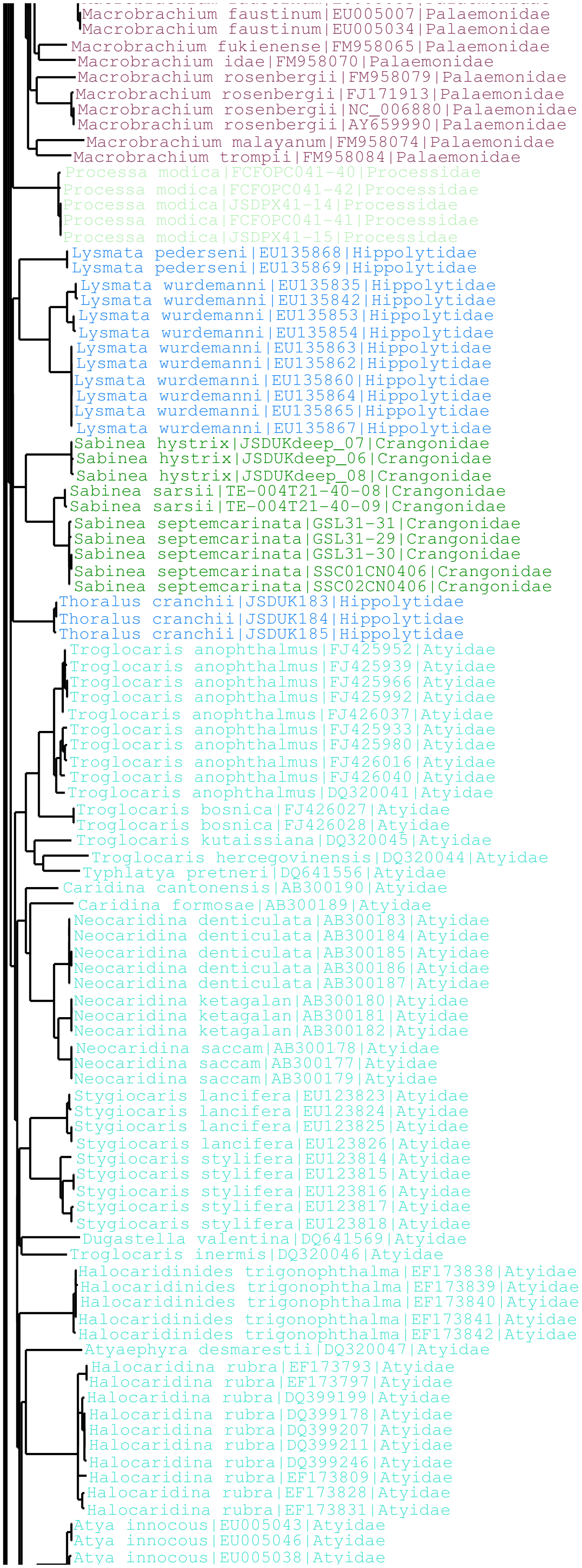
Macrobrachium faustinum|EU005007|Palaemonidae
Macrobrachium faustinum|EU005034|Palaemonidae
Macrobrachium fukienense|FM958065|Palaemonidae
Macrobrachium idae|FM958070|Palaemonidae
Macrobrachium rosenbergii|FM958079|Palaemonidae
Macrobrachium rosenbergii|FJ171913|Palaemonidae
Macrobrachium rosenbergii|NC_006880|Palaemonidae
Macrobrachium rosenbergii|AY659990|Palaemonidae
Macrobrachium malayanum|FM958074|Palaemonidae
Macrobrachium trompii|FM958084|Palaemonidae
Processa modica|FCFOPC041-40|Processidae
Processa modica|FCFOPC041-42|Processidae
Processa modica|JSDPX41-14|Processidae
Processa modica|FCFOPC041-41|Processidae
Processa modica|JSDPX41-15|Processidae
Lysmata pederseni|EU135868|Hippolytidae
Lysmata pederseni|EU135869|Hippolytidae
Lysmata wurdemanni|EU135835|Hippolytidae
Lysmata wurdemanni|EU135842|Hippolytidae
Lysmata wurdemanni|EU135853|Hippolytidae
Lysmata wurdemanni|EU135854|Hippolytidae
Lysmata wurdemanni|EU135863|Hippolytidae
Lysmata wurdemanni|EU135862|Hippolytidae
Lysmata wurdemanni|EU135860|Hippolytidae
Lysmata wurdemanni|EU135864|Hippolytidae
Lysmata wurdemanni|EU135865|Hippolytidae
Lysmata wurdemanni|EU135867|Hippolytidae
Sabinea hystrix|JSDUKdeep_07|Crangonidae
Sabinea hystrix|JSDUKdeep_06|Crangonidae
Sabinea hystrix|JSDUKdeep_08|Crangonidae
Sabinea sarsii|TE-004T21-40-08|Crangonidae
Sabinea sarsii|TE-004T21-40-09|Crangonidae
Sabinea septemcarinata|GSL31-31|Crangonidae
Sabinea septemcarinata|GSL31-29|Crangonidae
Sabinea septemcarinata|GSL31-30|Crangonidae
Sabinea septemcarinata|SSC01CN0406|Crangonidae
Sabinea septemcarinata|SSC02CN0406|Crangonidae
Thoralus cranchii|JSDUK183|Hippolytidae
Thoralus cranchii|JSDUK184|Hippolytidae
Thoralus cranchii|JSDUK185|Hippolytidae
Troglocaris anophthalmus|FJ425952|Atyidae
Troglocaris anophthalmus|FJ425939|Atyidae
Troglocaris anophthalmus|FJ425966|Atyidae
Troglocaris anophthalmus|FJ425992|Atyidae
Troglocaris anophthalmus|FJ426037|Atyidae
Troglocaris anophthalmus|FJ425933|Atyidae
Troglocaris anophthalmus|FJ425980|Atyidae
Troglocaris anophthalmus|FJ426016|Atyidae
Troglocaris anophthalmus|FJ426040|Atyidae
Troglocaris anophthalmus|DQ320041|Atyidae
Troglocaris bosnica|FJ426027|Atyidae
Troglocaris bosnica|FJ426028|Atyidae
Troglocaris kutaissiana|DQ320045|Atyidae
Troglocaris hercegovinensis|DQ320044|Atyidae
Typhlatya pretneri|DQ641556|Atyidae
Caridina cantonensis|AB300190|Atyidae
Caridina formosae|AB300189|Atyidae
Neocaridina denticulata|AB300183|Atyidae
Neocaridina denticulata|AB300184|Atyidae
Neocaridina denticulata|AB300185|Atyidae
Neocaridina denticulata|AB300186|Atyidae
Neocaridina denticulata|AB300187|Atyidae
Neocaridina ketagalan|AB300180|Atyidae
Neocaridina ketagalan|AB300181|Atyidae
Neocaridina ketagalan|AB300182|Atyidae
Neocaridina saccam|AB300178|Atyidae
Neocaridina saccam|AB300177|Atyidae
Neocaridina saccam|AB300179|Atyidae
Stygiocaris lancifera|EU123823|Atyidae
Stygiocaris lancifera|EU123824|Atyidae
Stygiocaris lancifera|EU123825|Atyidae
Stygiocaris lancifera|EU123826|Atyidae
Stygiocaris stylifera|EU123814|Atyidae
Stygiocaris stylifera|EU123815|Atyidae
Stygiocaris stylifera|EU123816|Atyidae
Stygiocaris stylifera|EU123817|Atyidae
Stygiocaris stylifera|EU123818|Atyidae
Dugastella valentina|DQ641569|Atyidae
Troglocaris inermis|DQ320046|Atyidae
Halocaridinides trigonophthalma|EF173838|Atyidae
Halocaridinides trigonophthalma|EF173839|Atyidae
Halocaridinides trigonophthalma|EF173840|Atyidae
Halocaridinides trigonophthalma|EF173841|Atyidae
Halocaridinides trigonophthalma|EF173842|Atyidae
Atyaephyra desmarestii|DQ320047|Atyidae
Halocaridina rubra|EF173793|Atyidae
Halocaridina rubra|EF173797|Atyidae
Halocaridina rubra|DQ399199|Atyidae
Halocaridina rubra|DQ399178|Atyidae
Halocaridina rubra|DQ399207|Atyidae
Halocaridina rubra|DQ399211|Atyidae
Halocaridina rubra|DQ399246|Atyidae
Halocaridina rubra|EF173809|Atyidae
Halocaridina rubra|EF173828|Atyidae
Halocaridina rubra|EF173831|Atyidae
Atya innocous|EU005043|Atyidae
Atya innocous|EU005046|Atyidae
Atya innocous|EU005038|Atyidae

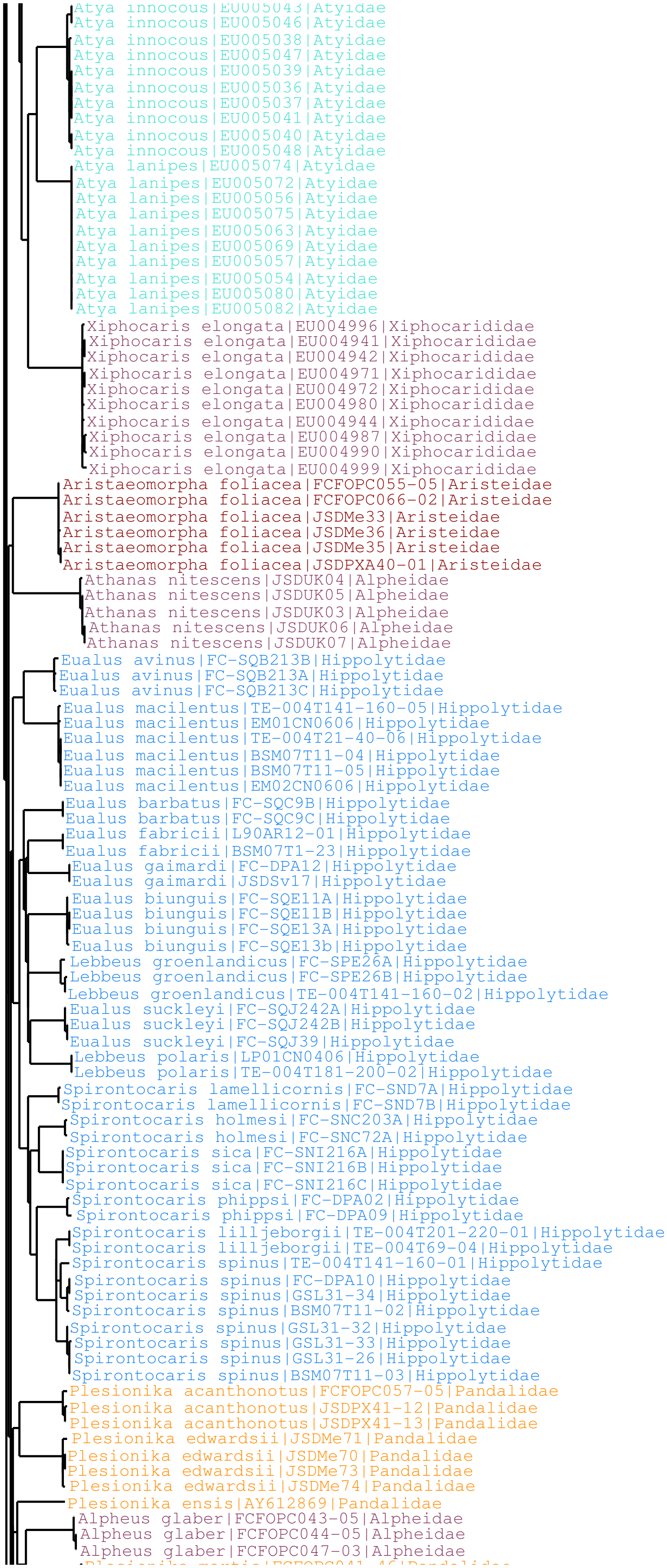
Atya innocous|EU005043|Atyidae
Atya innocous|EU005046|Atyidae
Atya innocous|EU005038|Atyidae
Atya innocous|EU005047|Atyidae
Atya innocous|EU005039|Atyidae
Atya innocous|EU005036|Atyidae
Atya innocous|EU005037|Atyidae
Atya innocous|EU005041|Atyidae
Atya innocous|EU005040|Atyidae
Atya innocous|EU005048|Atyidae
Atya lanipes|EU005074|Atyidae
Atya lanipes|EU005072|Atyidae
Atya lanipes|EU005056|Atyidae
Atya lanipes|EU005075|Atyidae
Atya lanipes|EU005063|Atyidae
Atya lanipes|EU005069|Atyidae
Atya lanipes|EU005057|Atyidae
Atya lanipes|EU005054|Atyidae
Atya lanipes|EU005080|Atyidae
Atya lanipes|EU005082|Atyidae
Xiphocaris elongata|EU004996|Xiphocarididae
Xiphocaris elongata|EU004941|Xiphocarididae
Xiphocaris elongata|EU004942|Xiphocarididae
Xiphocaris elongata|EU004971|Xiphocarididae
Xiphocaris elongata|EU004972|Xiphocarididae
Xiphocaris elongata|EU004980|Xiphocarididae
Xiphocaris elongata|EU004944|Xiphocarididae
Xiphocaris elongata|EU004987|Xiphocarididae
Xiphocaris elongata|EU004990|Xiphocarididae
Xiphocaris elongata|EU004999|Xiphocarididae
Aristaeomorpha foliacea|FCFOPC055-05|Aristeidae
Aristaeomorpha foliacea|FCFOPC066-02|Aristeidae
Aristaeomorpha foliacea|JSDMe33|Aristeidae
Aristaeomorpha foliacea|JSDMe36|Aristeidae
Aristaeomorpha foliacea|JSDMe35|Aristeidae
Aristaeomorpha foliacea|JSDPXA40-01|Aristeidae
Athanas nitescens|JSDUK04|Alpheidae
Athanas nitescens|JSDUK05|Alpheidae
Athanas nitescens|JSDUK03|Alpheidae
Athanas nitescens|JSDUK06|Alpheidae
Athanas nitescens|JSDUK07|Alpheidae
Eualus avinus|FC-SQB213B|Hippolytidae
Eualus avinus|FC-SQB213A|Hippolytidae
Eualus avinus|FC-SQB213C|Hippolytidae
Eualus macilentus|TE-004T141-160-05|Hippolytidae
Eualus macilentus|EM01CN0606|Hippolytidae
Eualus macilentus|TE-004T21-40-06|Hippolytidae
Eualus macilentus|BSM07T11-04|Hippolytidae
Eualus macilentus|BSM07T11-05|Hippolytidae
Eualus macilentus|EM02CN0606|Hippolytidae
Eualus barbatus|FC-SQC9B|Hippolytidae
Eualus barbatus|FC-SQC9C|Hippolytidae
Eualus fabricii|L90AR12-01|Hippolytidae
Eualus fabricii|BSM07T1-23|Hippolytidae
Eualus gaimardi|FC-DPA12|Hippolytidae
Eualus gaimardi|JSDSv17|Hippolytidae
Eualus biunguis|FC-SQE11A|Hippolytidae
Eualus biunguis|FC-SQE11B|Hippolytidae
Eualus biunguis|FC-SQE13A|Hippolytidae
Eualus biunguis|FC-SQE13b|Hippolytidae
Lebbeus groenlandicus|FC-SPE26A|Hippolytidae
Lebbeus groenlandicus|FC-SPE26B|Hippolytidae
Lebbeus groenlandicus|TE-004T141-160-02|Hippolytidae
Eualus suckleyi|FC-SQJ242A|Hippolytidae
Eualus suckleyi|FC-SQJ242B|Hippolytidae
Eualus suckleyi|FC-SQJ39|Hippolytidae
Lebbeus polaris|LP01CN0406|Hippolytidae
Lebbeus polaris|TE-004T181-200-02|Hippolytidae
Spirontocaris lamellicornis|FC-SND7A|Hippolytidae
Spirontocaris lamellicornis|FC-SND7B|Hippolytidae
Spirontocaris holmesi|FC-SNC203A|Hippolytidae
Spirontocaris holmesi|FC-SNC72A|Hippolytidae
Spirontocaris sica|FC-SNI216A|Hippolytidae
Spirontocaris sica|FC-SNI216B|Hippolytidae
Spirontocaris sica|FC-SNI216C|Hippolytidae
Spirontocaris phippsi|FC-DPA02|Hippolytidae
Spirontocaris phippsi|FC-DPA09|Hippolytidae
Spirontocaris lilljeborgii|TE-004T201-220-01|Hippolytidae
Spirontocaris lilljeborgii|TE-004T69-04|Hippolytidae
Spirontocaris spinus|TE-004T141-160-01|Hippolytidae
Spirontocaris spinus|FC-DPA10|Hippolytidae
Spirontocaris spinus|GSL31-34|Hippolytidae
Spirontocaris spinus|BSM07T11-02|Hippolytidae
Spirontocaris spinus|GSL31-32|Hippolytidae
Spirontocaris spinus|GSL31-33|Hippolytidae
Spirontocaris spinus|GSL31-26|Hippolytidae
Spirontocaris spinus|BSM07T11-03|Hippolytidae
Plesionika acanthonotus|FCFOPC057-05|Pandalidae
Plesionika acanthonotus|JSDPX41-12|Pandalidae
Plesionika acanthonotus|JSDPX41-13|Pandalidae
Plesionika edwardsii|JSDMe71|Pandalidae
Plesionika edwardsii|JSDMe70|Pandalidae
Plesionika edwardsii|JSDMe73|Pandalidae
Plesionika edwardsii|JSDMe74|Pandalidae
Plesionika ensis|AY612869|Pandalidae
Alpheus glaber|FCFOPC043-05|Alpheidae
Alpheus glaber|FCFOPC044-05|Alpheidae
Alpheus glaber|FCFOPC047-03|Alpheidae

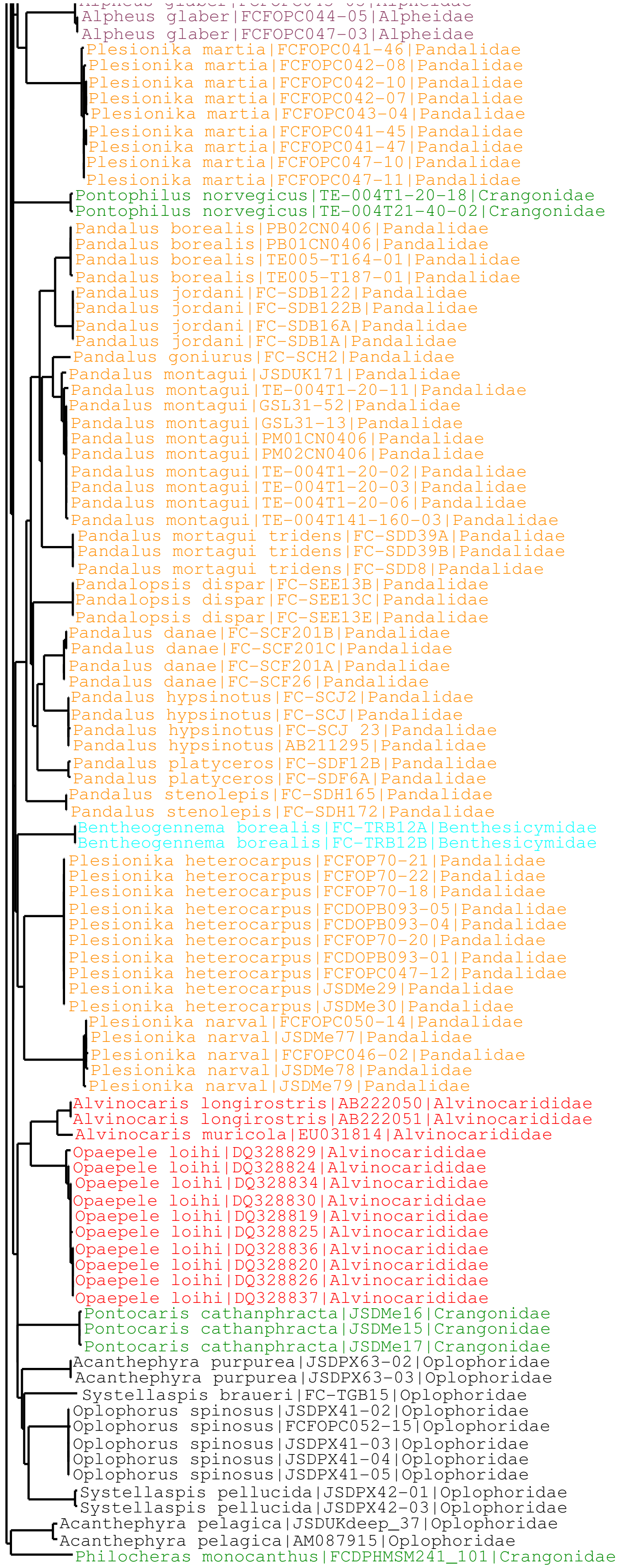
Alpheus glaber|FCFOPC044-05|Alpheidae
Alpheus glaber|FCFOPC047-03|Alpheidae
Plesionika martia|FCFOPC041-46|Pandalidae
Plesionika martia|FCFOPC042-08|Pandalidae
Plesionika martia|FCFOPC042-10|Pandalidae
Plesionika martia|FCFOPC042-07|Pandalidae
Plesionika martia|FCFOPC043-04|Pandalidae
Plesionika martia|FCFOPC041-45|Pandalidae
Plesionika martia|FCFOPC041-47|Pandalidae
Plesionika martia|FCFOPC047-10|Pandalidae
Plesionika martia|FCFOPC047-11|Pandalidae
Pontophilus norvegicus|TE-004T1-20-18|Crangonidae
Pontophilus norvegicus|TE-004T21-40-02|Crangonidae
Pandalus borealis|PB02CN0406|Pandalidae
Pandalus borealis|PB01CN0406|Pandalidae
Pandalus borealis|TE005-T164-01|Pandalidae
Pandalus borealis|TE005-T187-01|Pandalidae
Pandalus jordani|FC-SDB122|Pandalidae
Pandalus jordani|FC-SDB122B|Pandalidae
Pandalus jordani|FC-SDB16A|Pandalidae
Pandalus jordani|FC-SDB1A|Pandalidae
Pandalus goniurus|FC-SCH2|Pandalidae
Pandalus montagui|JSDUK171|Pandalidae
Pandalus montagui|TE-004T1-20-11|Pandalidae
Pandalus montagui|GSL31-52|Pandalidae
Pandalus montagui|GSL31-13|Pandalidae
Pandalus montagui|PM01CN0406|Pandalidae
Pandalus montagui|PM02CN0406|Pandalidae
Pandalus montagui|TE-004T1-20-02|Pandalidae
Pandalus montagui|TE-004T1-20-03|Pandalidae
Pandalus montagui|TE-004T1-20-06|Pandalidae
Pandalus montagui|TE-004T141-160-03|Pandalidae
Pandalus mortagui tridens|FC-SDD39A|Pandalidae
Pandalus mortagui tridens|FC-SDD39B|Pandalidae
Pandalus mortagui tridens|FC-SDD8|Pandalidae
Pandalopsis dispar|FC-SEE13B|Pandalidae
Pandalopsis dispar|FC-SEE13C|Pandalidae
Pandalopsis dispar|FC-SEE13E|Pandalidae
Pandalus danae|FC-SCF201B|Pandalidae
Pandalus danae|FC-SCF201C|Pandalidae
Pandalus danae|FC-SCF201A|Pandalidae
Pandalus danae|FC-SCF26|Pandalidae
Pandalus hypsinotus|FC-SCJ2|Pandalidae
Pandalus hypsinotus|FC-SCJ|Pandalidae
Pandalus hypsinotus|FC-SCJ 23|Pandalidae
Pandalus hypsinotus|AB211295|Pandalidae
Pandalus platyceros|FC-SDF12B|Pandalidae
Pandalus platyceros|FC-SDF6A|Pandalidae
Pandalus stenolepis|FC-SDH165|Pandalidae
Pandalus stenolepis|FC-SDH172|Pandalidae
Bentheogennema borealis|FC-TRB12A|Benthesicymidae
Bentheogennema borealis|FC-TRB12B|Benthesicymidae
Plesionika heterocarpus|FCFOP70-21|Pandalidae
Plesionika heterocarpus|FCFOP70-22|Pandalidae
Plesionika heterocarpus|FCFOP70-18|Pandalidae
Plesionika heterocarpus|FCDOPB093-05|Pandalidae
Plesionika heterocarpus|FCDOPB093-04|Pandalidae
Plesionika heterocarpus|FCFOP70-20|Pandalidae
Plesionika heterocarpus|FCDOPB093-01|Pandalidae
Plesionika heterocarpus|FCFOPC047-12|Pandalidae
Plesionika heterocarpus|JSDMe29|Pandalidae
Plesionika heterocarpus|JSDMe30|Pandalidae
Plesionika narval|FCFOPC050-14|Pandalidae
Plesionika narval|JSDMe77|Pandalidae
Plesionika narval|FCFOPC046-02|Pandalidae
Plesionika narval|JSDMe78|Pandalidae
Plesionika narval|JSDMe79|Pandalidae
Alvinocaris longirostris|AB222050|Alvinocarididae
Alvinocaris longirostris|AB222051|Alvinocarididae
Alvinocaris muricola|EU031814|Alvinocarididae
Opaepele loihi|DQ328829|Alvinocarididae
Opaepele loihi|DQ328824|Alvinocarididae
Opaepele loihi|DQ328834|Alvinocarididae
Opaepele loihi|DQ328830|Alvinocarididae
Opaepele loihi|DQ328819|Alvinocarididae
Opaepele loihi|DQ328825|Alvinocarididae
Opaepele loihi|DQ328836|Alvinocarididae
Opaepele loihi|DQ328820|Alvinocarididae
Opaepele loihi|DQ328826|Alvinocarididae
Opaepele loihi|DQ328837|Alvinocarididae
Pontocaris cathanphracta|JSDMe16|Crangonidae
Pontocaris cathanphracta|JSDMe15|Crangonidae
Pontocaris cathanphracta|JSDMe17|Crangonidae
Acanthephyra purpurea|JSDPX63-02|Oplophoridae
Acanthephyra purpurea|JSDPX63-03|Oplophoridae
Systellaspis braueri|FC-TGB15|Oplophoridae
Oplophorus spinosus|JSDPX41-02|Oplophoridae
Oplophorus spinosus|FCFOPC052-15|Oplophoridae
Oplophorus spinosus|JSDPX41-03|Oplophoridae
Oplophorus spinosus|JSDPX41-04|Oplophoridae
Oplophorus spinosus|JSDPX41-05|Oplophoridae
Systellaspis pellucida|JSDPX42-01|Oplophoridae
Systellaspis pellucida|JSDPX42-03|Oplophoridae
Acanthephyra pelagica|JSDUKdeep_37|Oplophoridae
Acanthephyra pelagica|AM087915|Oplophoridae
Philocheras monocanthus|FCDPHMSM241_101|Crangonidae
